# Algorithm-Driven Robotic Discovery of Polyoxometalate-Scaffolding Metal–Organic Frameworks

**DOI:** 10.1021/jacs.4c09553

**Published:** 2024-10-09

**Authors:** Donglin He, Yibin Jiang, Melanie Guillén-Soler, Zack Geary, Lucia Vizcaíno-Anaya, Daniel Salley, Maria Del Carmen Gimenez-Lopez, De-Liang Long, Leroy Cronin

**Affiliations:** † School of Chemistry, University of Glasgow, University Avenue, Glasgow G12 8QQ, United Kingdom; ‡ Centro Singular de Investigación en Química Biolóxica e Materiais Moleculares (CiQUS), Universidade de Santiago de Compostela, Santiago de Compostela 15782, Spain

## Abstract

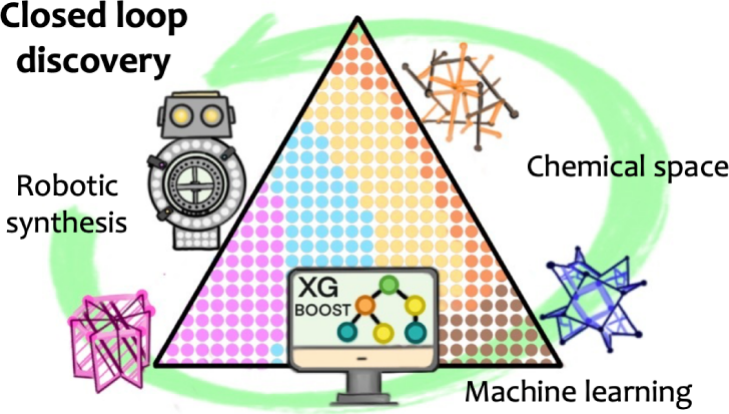

The experimental exploration of the chemical space of crystalline materials, especially metal–organic frameworks (MOFs), requires multiparameter control of a large set of reactions, which is unavoidably time-consuming and labor-intensive when performed manually. To accelerate the rate of material discovery while maintaining high reproducibility, we developed a machine learning algorithm integrated with a robotic synthesis platform for closed-loop exploration of the chemical space for polyoxometalate-scaffolding metal–organic frameworks (POMOFs). The eXtreme Gradient Boosting (XGBoost) model was optimized by using updating data obtained from the uncertainty feedback experiments and a multiclass classification extension based on the POMOF classification from their chemical constitution. The digital signatures for the robotic synthesis of POMOFs were represented by the universal chemical description language (χDL) to precisely record the synthetic steps and enhance the reproducibility. Nine novel POMOFs including one with mixed ligands derived from individual ligands through the imidization reaction of POM amine derivatives with various aldehydes have been discovered with a good repeatability. In addition, chemical space maps were plotted based on the XGBoost models whose F1 scores are above 0.8. Furthermore, the electrochemical properties of the synthesized POMOFs indicate superior electron transfer compared to the molecular POMs and the direct effect of the ratio of Zn, the type of ligands used, and the topology structures in POMOFs for modulating electron transfer abilities.

## Introduction

Polyoxometalates (POMs) are anionic metal–oxygen clusters with diverse structures that consist of early transition metal ions (V, Mo, W, etc.) in high oxidation states (+5 and + 6) that are bridged by μ_*x*_ -oxygen atoms. They have been widely studied for their electrochemical,^1^ catalytic,^2^ bioactive,^3^ optical,^4^ and magnetic^5^ properties. Recently, the incorporation of POMs into metal organic frameworks (MOFs) has been investigated for discovering novel topologies and multifunctional POM-based MOFs by associating both the properties of the MOFs and POMs in the same system.^6−8^ In POM-based MOFs, POMs can be guest molecules encapsulated in the cavity of MOFs (POM@MOFs)^9^ and act as secondary building units (SBUs)^10^ or as part of the framework linker bound with metal centers and organic linkers,^11^ thus forming polyoxometalate-scaffolding MOFs (POMOFs). The main focus of this work is POMOF systems in the final category, where POMs are part of the framework and bound with metal centers and organic linkers.

Organic functionalization of POMs is an important strategy for integrating them into the structure of POMOFs via covalent or coordinate bonds. In recent years, Tris-functionalized Anderson–Evans (AE)-type POMs have helped achieve significant advances in the construction of POMOFs. In 2007, we reported a polymeric framework in the solid state in which the repeating unit of the chain is built from ditopic amino-functionalized Tris-AE cluster units connected via a bridging {Ag_2_(DMSO)_4_}^2+^ unit. The polymeric chain is propagated by a single Ag(I) ion, which connects the nitrogen atom of the Tris-AE ligands, while a further {Ag(DMSO)_3_}^+^ unit decorates the AE POM cluster.^12^ The amino-functionalized Tris-AE POM has also been used by Xu et al. for constructing a three-dimensional MOF via imine condensation with 4-connected tetrahedral tetrakis(4-formylphenyl)methane.^10^ Carboxylate-functionalized Tris-AE POMs have recently been reported to form three-dimensional POMOFs with lanthanide ions as nodes via the different crystallization processes including a stirring and layering method.^13^ A series of POMOFs has been reported that were built from different pyridine-functionalized Tris-AE hybrids acting as linkers coordinating with Zn^2+^ and Cu^2+^.^11^ Despite the presence of pyridyl sites, it was possible for the terminal oxo units of the Tris-AE POM to coordinate with Zn^2+^ ions, making it difficult to predict the resultant structure of the POMOF.^11^ Therefore, considering the large numbers of variables associated with the synthesis, such as the cation content, coligands, competing ligands, and heating time, the discovery of POMOFs based on the pyridine-functionalized Tris-AE POMs can be both unpredictable and labor-intensive.

In order to explore the chemical space for the systems involving the multiparameter control of complicated processes and requiring a large set of reactions, automated systems with customized machine learning (ML) algorithms recently have been used to reduce labor and accelerate materials discovery.^14,15^ Various machine learning models such as support vector machines (SVM),^16^ random forest (RF) regression,^17^ neural networks,^18^ and gradient boosting algorithms^19^ have been applied for guiding chemical synthesis to more efficiently explore chemical space. Among them, eXtreme Gradient Boosting (XGBoost), a boosting algorithm based on a regularized ML boosting tree model, can be used effectively and reliably in the prediction and mining of classification and regression tasks.^19,20^ The performance of XGBoost has been compared with other machine learning algorithms for predicting the crystallization propensity of metal–organic nanocapsules (MONCs) by a set of training data including both successful and failed experiments.^19^ Here, the XGBoost model, with a prediction accuracy of >91%, helped by increasing the speed at which the optimal reaction parameters were defined from a large set of variables, resulted in the successful discovery of a new set of crystalline MONCs.^19^ POMOF synthesis also has a wide range of experimental variables in high dimensionality and therefore can also be guided by XGBoost for investigating the crystallization propensity in a cost-effective sampling strategy.

In this work, we performed a closed-loop exploration of the crystallization boundaries of δ-Tris-AE POM-based POMOFs. This work was facilitated and enriched by an approach that used two technologically advanced methods synergistically. The XGBoost algorithm was trained to suggest reaction conditions and predict the probability of successful crystallizations, while a robotic system executed the suggested reactions and generated further data, which the XGBoost algorithm could learn from and use to suggest further reactions. The assembly of the database, which is the crucial element of this approach for the discovery of new POMOFs, was carried out in a feedback loop based on the experimental result of reactions that had the most uncertainty associated with their predicted outcome, as determined by the XGBoost algorithm. Both algorithm design principles and chemistry knowledge were utilized in the sampling process to enhance the model’s knowledge of the chemical space. The chemical space map of a variety of POMOFs was established during the process, which led to the discovery of new POMOFs including one mixed-ligand POMOF. Structural, compositional, stability, gas sorption, and electrochemical characterizations of the novel POMOFs were achieved alongside chemical space map generation, demonstrating the level of efficiency that is made possible through the combination of ML and synthetic automation.

## Materials and Methods

### Synthesis Design

**Figure 1:**
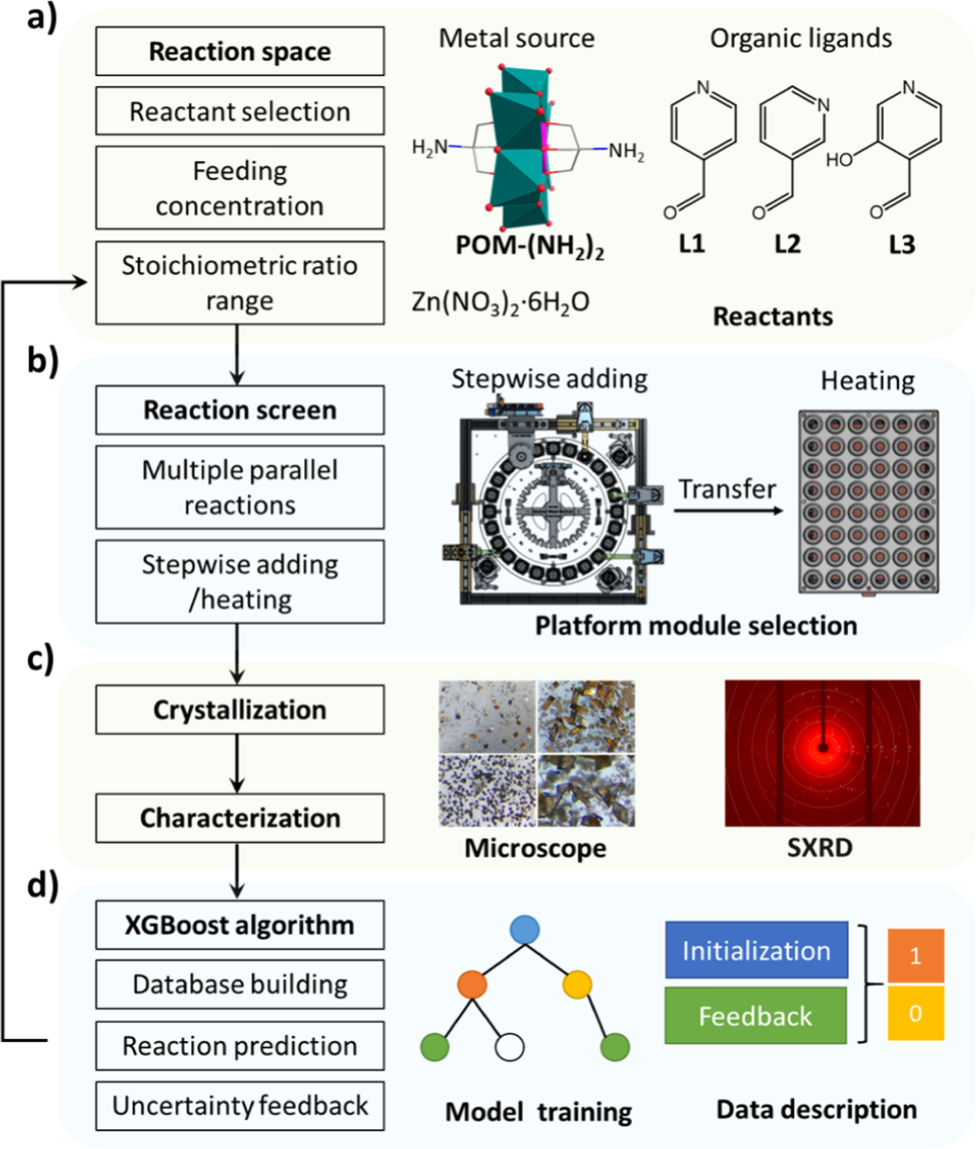
Schematic representation of the robotic discovery of POMOFs: (a) reaction space for the synthesis of POMOFs in one-pot, (b) robotic platform used for carrying out reactions, (c) the confirmation and record of reaction results, and (d) uncertainty feedback for optimizing the machine learning model.

As shown in Figure 1a, the δ-Tris-based Mn–Anderson–Evans (POM–(NH_2_)_2_, [N(C_4_H_9_)_4_]_3_[MnMo_6_O_18_{(OCH_2_)_3_CNH_2_}_2_]) were selected as both the metal sources and the linkers that can connect with the selected aldehyde by forming an imine bond, which was synthesized as described previously.^21^ 4-pyridinecarboxaldehyde (L1), 3-pyridinecarboxaldehyde (L2), and 3-hydroxypyridine-4-carboxaldehyde (L3) were selected as organic ligands. Two pyridine ligand-functionalized AE POMs were previously obtained by the imidization reaction of POM–(NH_2_)_2_ with L1 and L2, which can form coordination polymers with [Cu(PPh_3_)_2_(CH_3_CN)_2_]ClO_4_.^22^ L3 with hydroxyl, aldehyde substituents, and similar pyridine structure to L1 has been reported to exhibit green fluorescence as the lowest molecular weight of all dyes.^23^ Inspired by the coordination-driven self-assembled capsules formed by the one-pot reaction with pyridine aldehydes, amine, and metal ions,^24,25^ the discovery of POMOFs in this work followed a similar one-pot synthetic procedure with Zn(NO_3_)_2_·6H_2_O as the second metal node. The reaction involves the formation of dynamic covalent (N=C) and coordinative (N→M and O→M) bonds under thermodynamic control, in which the reactant concentration and ratio can be controlled for the formation of POMOFs in different topologies. Therefore, the reactant concentrations, single ligands, and competing ligands were focused on to explore the chemical space and crystallization boundaries of the POMOFs in a robotic system with the customized XGBoost algorithm.

#### Robotic Platform

The core robotic hardware shown in Figures 1b and S1 consists of a chemical reaction/mixing module capable of performing parallel synthesis in up to 24 reactors and a larger heating mantle unit that can hold up to 48 reactions (14 mL vials).^26^ By using the rotation of the Geneva wheel and high-precision syringe pumps, the chemical reaction module performs liquid handling to achieve highly accurate control of the volume of reagents dispensed into reactant solutions. POM–(NH_2_)_2_, L1, L2, L3, and Zn(NO_3_)_2_·6H_2_O were dissolved in *N*,*N*-dimethylformamide (DMF) separately as stock solutions. To finely control the concentration of reactants dispensed, neat DMF was also used as a stock solution, allowing adjustments to reactive reagent stock solutions in real time. The volume dispensed from the reagent stock solutions ranged from 0 to 2 mL. After reagents were added, the vials were sealed and transferred manually to the heating mantle unit set at 80 °C (actual temperature in the solution was observed to be 65 ± 3 °C). All vials were left to stand for 48 h without stirring, after which images of each vial were obtained by microscope photography for initially confirming if there are any single crystals (Figure 1c). Crystal structures were confirmed by single crystal X-ray diffraction (SC-XRD).

#### Model Optimization Loop

Figure 1d indicates the feedback loop for training XGBoost models to describe crystallization boundaries of POMOFs with the assistance of the robotic system. Normally, input and output data settings for machine learning models are collected from a large database based on random or manual design experiments.^18−20^ Thus far, most attention has been paid toward choosing a suitable algorithm rather than a suitable strategy for obtaining high-quality and relevant data. However, an efficient and reliable strategy for collecting data not only enhances the quality of data but also helps limit the number of unnecessary experiments, significantly reducing the project’s cost while improving the reliability of the model. Therefore, we opted to collect data in a step-by-step approach, in each instance, performing the reaction with the greatest uncertainty as predicted by the XGBost algorithm. The type, concentration, and dispensing sequence of the stock solutions were decided by carrying out a synthetic screen in the robotic platform (details shown in Supporting Information Section 2.1). The added volume of POM–(NH_2_)_2_ (0.03 mmol/mL), L1 (0.12 mmol/L), L2 (0.12 mmol/mL), L3 (0.12 mmol/L), and Zn(NO_3_)_2_·6H_2_O (0.045 mmol/mL) solutions and DMF were identified as the 6 most significant variables in the formation of POMOF single crystals. In order to promote imine bond formation, the POM–(NH_2_)_2_, organic ligands, and DMF were added in sequence, and then the Zn(NO_3_)_2_·6H_2_O was added after 1 h. After that, the vials were sealed, transferred manually to the heating mantle, and heated at 80 °C for 48 h.

The initial experiments (42) were carried out by the robotic platform based on either random (20) or manual design (22). Once complete, data used to describe the result of reactions were collected by microscope photography and SC-XRD. The qualitative descriptions of the reaction results were categorized into two classes: class “0” indicates the reaction result in which single crystals for the target POMOFs were not formed, while class “1” indicates the reaction result in which single crystals of the target POMOF were formed. A sample is further checked by SC-XRD to ensure that it is correctly assigned to class “1” after the initial check by microscope photography. The extra confirmation step by SC-XRD is necessary because not all single crystal products are the desired POMOFs. For example, it was found that the POM–(NH_2_)_2_ can directly coordinate with zinc ions to form linear coordination polymer (POM–Zn) crystals, and the aldehyde can be oxidized into the corresponding acid crystals in DMF (Table S39). These results would therefore be classified in class “0″ as despite the samples producing single crystals, they are not single crystals of the desired POMOF. The results with soluble, amorphous products or microcrystalline products whose structures cannot be confirmed by SC-XRD would also be classified in class “0.”

It is impossible for these POMOFs to form if POM–(NH_2_)_2_ or Zn(NO_3_)_2_·6H_2_O is not present in a reaction system. Therefore, the results of 65 reactions were set as class “0” as a part of the initialization data set because even if they were performed, there would be a 0% chance that the reactions would yield a result of class 1 (see Supporting Information Section 2.3). In the initialization data set, a total of 107 reactions were classified into class “0” and class “1” with a ratio of 81:26, respectively. The initialization data sets were further shuffled and split into training (80%) and test data sets (20%) for training the first XGBoost machine learning model (the details for the XGBoost algorithm are in Section 1.4 of Supporting Information).

**Figure 2:**
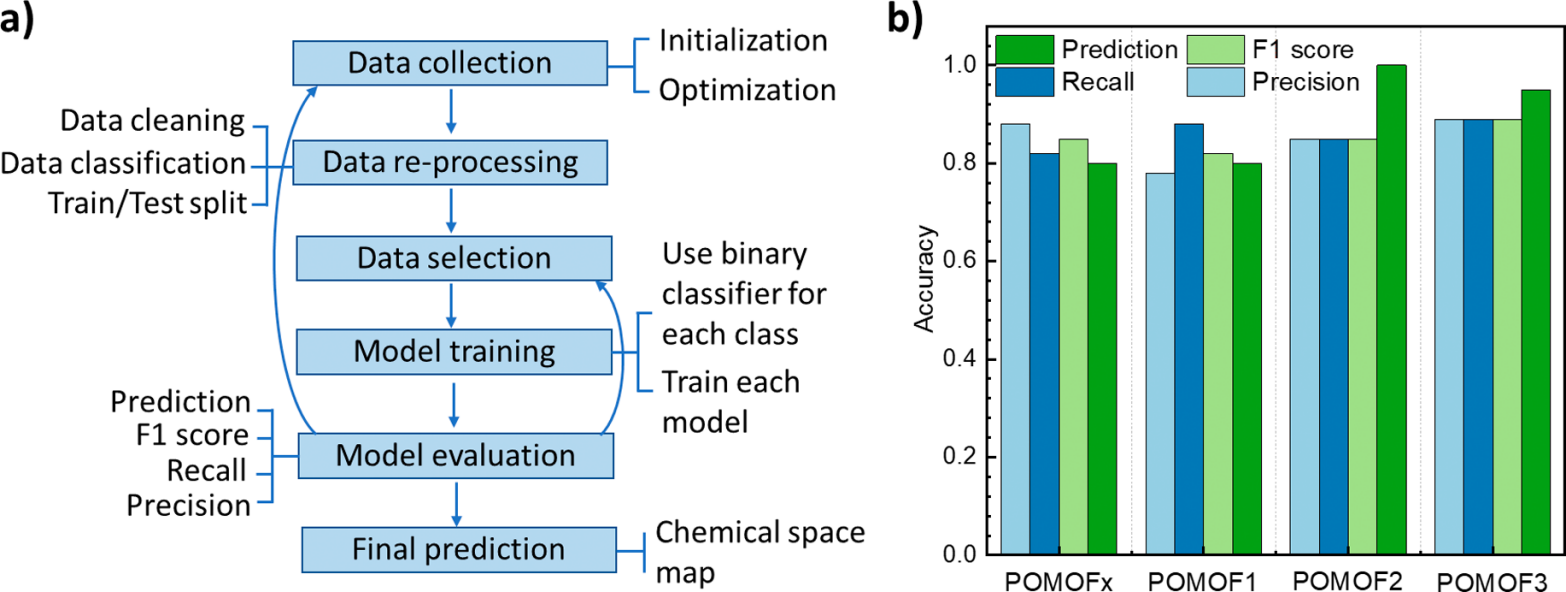
(a) Schematic representation of working flow for extending the binary classification into the multiclass classification by transforming the multiclass problem into multiple independent binary classification problems. (b) Accuracy of the final model for each independent binary classification.

After being trained on the initialization data set, the uncertainty of the experimental results from different synthesis conditions were evaluated based on the model. In this context, uncertainty is associated with the classification probability from the model for the given experimental conditions. Additionally, the sampling process of the potential experimental conditions considered the sampling diversity in the chemical space (see Supporting Information section 1.4). Following the initialization of the algorithm, the chemical space was explored in parallel with model refinement by an iterative method: the 10 reactions with the highest degree of prediction uncertainty, as given by the algorithm, were performed, analyzed, and classified as previously described. Once the reaction conditions were completed and the corresponding results were added to the model’s total data set, the model is retrained and used to generate a new set of reaction conditions. This process was iterated until the exploration was finished. The whole optimization loop for the machine learning model is shown in Figures 1d and 2.

## Results and Discussion

### Evaluation of the Model

We recorded the accuracy of the model based on the updated data for each optimization cycle. Precision (*P*) is the ratio of the correctly predicted positive observations to the total predicted positives, assessing the accuracy of the positive predictions. Recall (*R*) is the ratio of the correctly predicted positive observations to all of the actual positives, measuring the ability of the model to capture all of the relevant cases. F1 score (F1) is the harmonic mean of precision and recall, which provides a balance between *P* and *R*, making it a useful metric when there is an uneven class distribution. Prediction (PD) is the ratio of the number of correctly predicted results in a set compared with the total number of experiments in the said set (Table S54). All of the calculation details are shown in Supporting Information Section 1.4.

**Figure 3:**
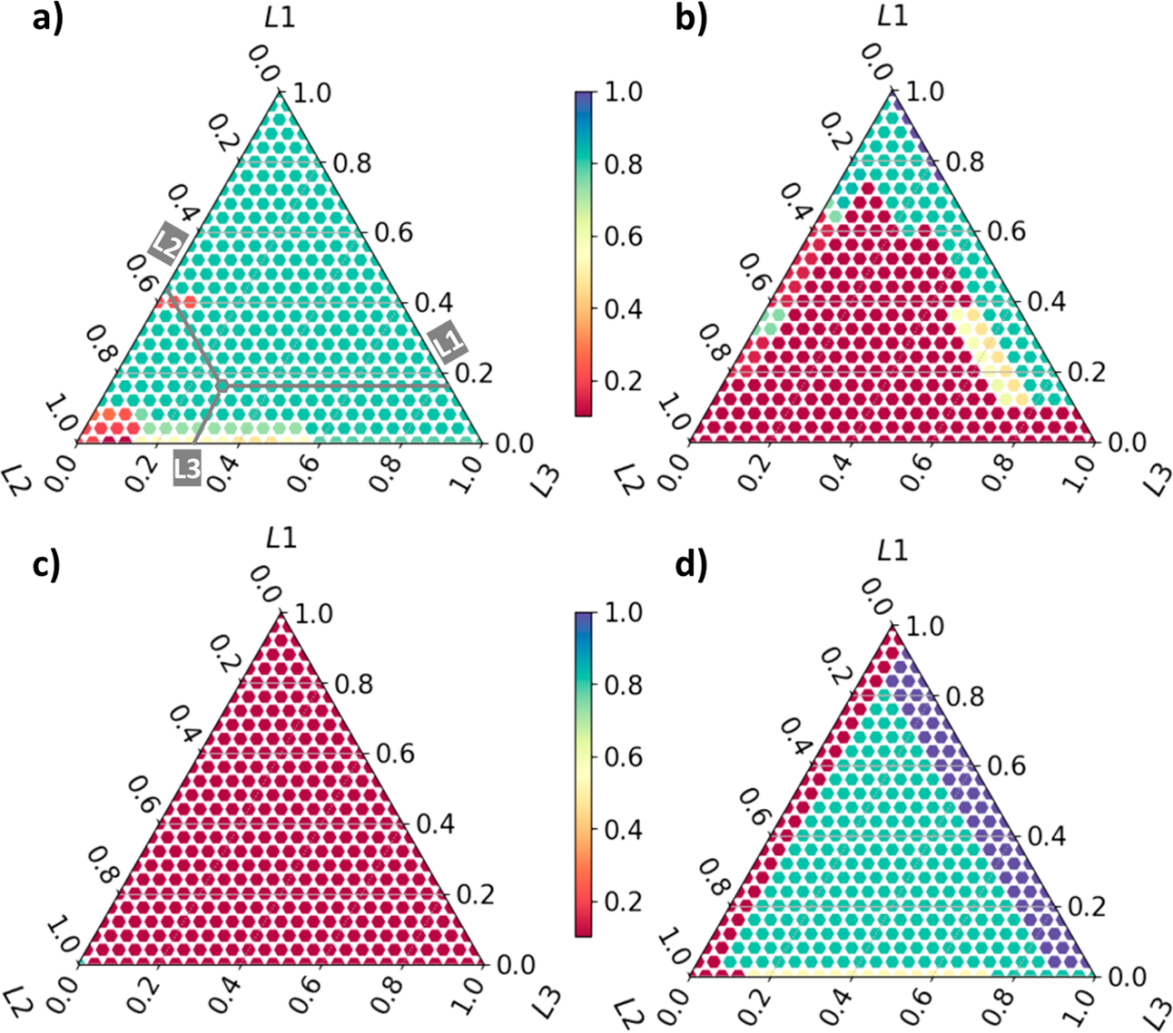
Chemical space map predicted by the XGBoost for (a) POMOFx, (b) POMOF1, (c) POMOF2, and (d) POMOF3. Adding volume for POM–(NH_2_)_2_ is fixed in 1 mL; color bar: the possibility for obtaining the target POMOF single crystals.

As shown in Figure S35, the F1 score of the model trained from the initialization data is 67%. The F1 score was improved significantly to 92% after the second run. To increase the training sample size and to discover more POMOF structures, 8 optimization cycles were run in the first stage, in which the F1 score fluctuated around 80%. In this stage, 7 new POMOFs had been discovered (POMOF1–1, POMOF1–2, POMOF2–1, POMOF2–3, POMOF3–1, POMOF3–2, and POMOF1 + 3 are all shown in Figure 3) by mapping the chemical space through model uncertainty. The nomenclature is given as follows: for general POMOFx–y, x represents the ligand types L1, L2 and L3, while *y* represents the number of phases from the same ligand. According to their composition, the resultant POMOFs can be categorized into three classes: (i) POMOF1, (ii) POMOF2, and (iii) POMOF3. A notable exception is POMOF1+3, which contains L1 and L3 as mixed ligands and therefore belongs to both classes i and iii.

Applying the target POMOF classification strategy, for POMOF1 systems, a reaction would be put into class “1″ if it produced single crystals that contained a POMOF with the L1 ligand. Any other outcome would result in the reaction being classified as “0.” This strategy was extended to the POMOF2 and POMOF3 systems. After the first 8 optimization cycles, the POMOF1 system had 63 reactions in class “1.” In regard to the POMOF2 and POMOF3 systems, there were 3 and 78 class “1” reactions, respectively.

To further explore the crystallization boundaries of the POMOF2 system, which was predicted to have a low probability of a successful synthesis according to the previous data, we performed 10 reactions. In these reactions, only L2 was added as the ligand, while L1 and L3 were completely omitted. These reactions were predicted to be class “1” by the all-inclusive POMOF model (POMOFx, the model that is trained on a combined data set of all POMOF–ligand systems). However, the experiment results show that only 5 reactions formed single crystals of POMOF2 (Table S33, the data were also used as the ninth feedback optimization data). This means that the model trained from the first 8 cycles cannot predict the POMOF2 system very well, despite having a high F1 score. Then, 20 reactions involving all ligands were designed to compare the prediction results to actual reaction outcomes. For the POMOFx system, these two results match very well (16/20 attack rating in Table S54 and Figure S36), which implies that the low accuracy of the POMOF2 system model can be attributed to the very small number of class “1” results for POMOF2 in the training data set.

### Extend the Binary Classification into the Multiclass Classification

With the aim of improving the accuracy of the model for all POMOF systems, especially for POMOF2, the independent binary classification model for POMOF2 systems was optimized by running more experiments through the feedback loop process. In parallel, the accuracy of the model, which continuously changed in response to receiving updated data for POMOFx, POMOF1, and POMOF3 systems, was calculated (Figures S36–S39). Therefore, multiclass classification was achieved by transforming the multiclass problem into multiple independent binary classification problems (Figure 2a). The accuracy shown in Figure 2b was recorded for each class to describe the chemical space.

As shown in Figure S38, the F1 for the POMOF2 system was recorded from the 11th cycle. This is because there are too few class “1” results to train the model and calculating the F1 score is inappropriate. Based on the large number of negative results, the classification model aims to select the conditions with high uncertainty, which also tend to form POMOF2 crystals. The selected reaction conditions with high uncertainty are mostly those without L1 and L3 added, which indicates that adding L2 as the only organic ligands would improve the possibility of forming POMOF2 crystals (Table S37). It should be noted that data cleaning, which included removing duplicate data and checking uncertain data, was carried out during the process. After 18 cycles, the F1 for the POMOF2 system reached 0.85, and the PD, *R*, and *P* were 1.00, 0.85, and 0.85, respectively, which is the end of the collection of data. Selecting the most appropriate data set based on it having an F1 of >0.8 and the relative highest PD, the final models for POMOFx, POMOF1, POMOF2, and POMOF3 were trained based on the data of 12, 12, 18, and 14 cycles, respectively (the details are in Supporting Information Section 3).

**Figure 4:**
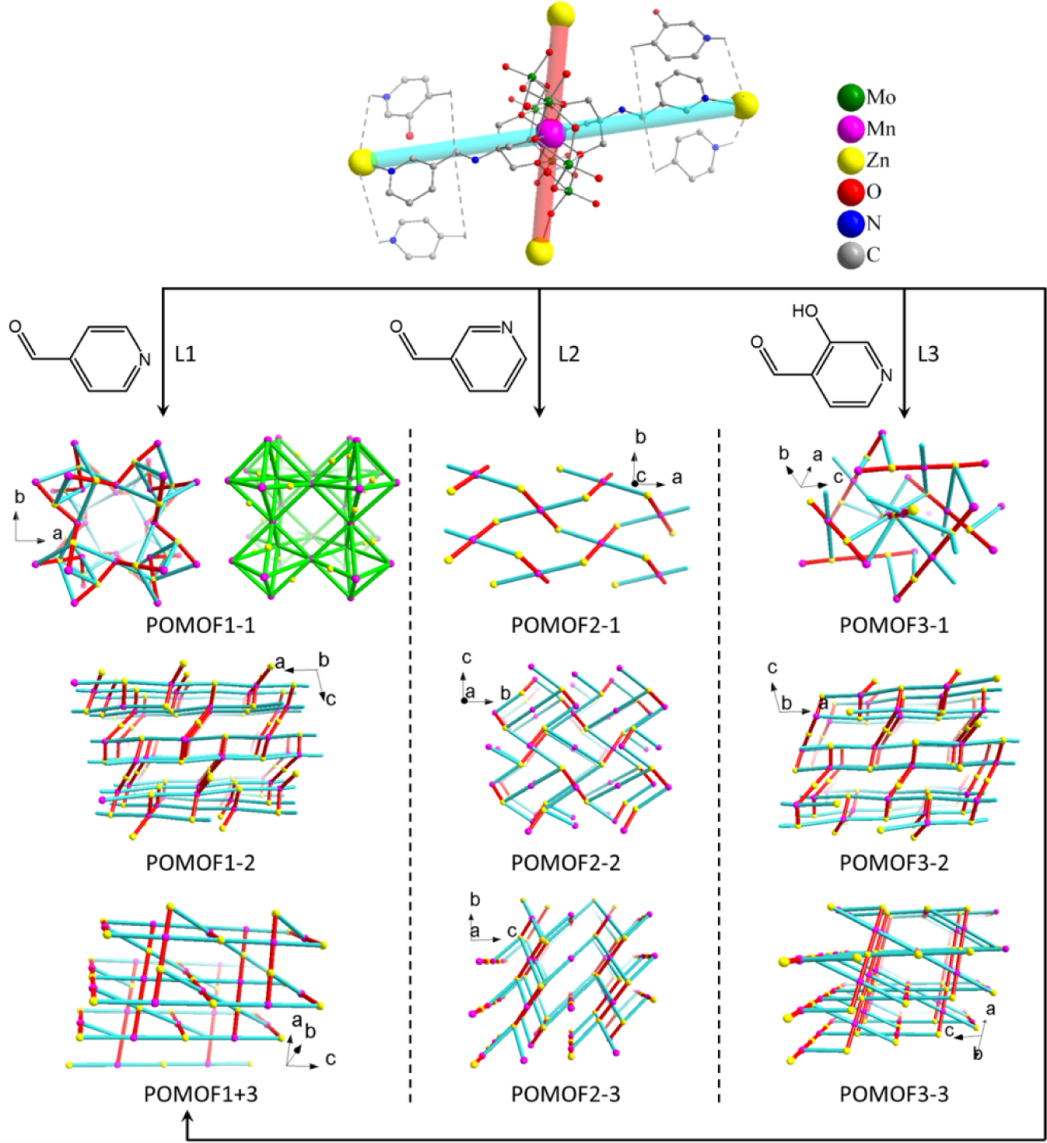
Topology structures of POMOFs based on single crystal structures. The node formed by the AE cluster is simplified as pink Mn atoms with connection via pyridyl coordination in cyan color and connection via oxo coordination in red color. For POMOF1–1, the bright green colored framework beside shows the zeolite A-type structure formed by Mn sites (pink) as octahedral apexes with the Zn site (yellow) located on some edge centers. POMOF1–1, POMOF2–1, and POMOF3–1 have 3-D cubic, 2-D layer, and 3-D 4-connected topology, respectively. POMOF1–2 and POMOF3–2 have the same 3D (4,3) connected topology. POMOF2–2 and POMOF2–3 have the same another 3D (4,3) connected topology. POMOF1+3 and POMOF3–3 have the same 3-D 4-connected topology.

As shown in Figure 2b, the F1 scores for the final models for POMOFx, POMOF1, POMOF2, and POMOF3 systems are 0.85, 0.82, 0.85, and 0.89, respectively. In addition, their respective PD value is 0.80, 0.80, 1.00, and 0.95. Therefore, they not only exhibit high theoretical accuracy but also demonstrate the ability to accurately predict reaction outcomes when compared to actual experimental results. Furthermore, the optimization loops based on the POMOF2 system discovered two new phases, POMOF2–2 and POMOF3–3, as shown in Figure 4.

### Chemical Space of POMOFs

To create visual representations of data with 6 dimensions, chemical space maps based on the models for POMOFx, POMOF1, POMOF2, and POMOF3 were plotted by dimensionality reduction. The resulting triangular plots, as shown in Figure 3, illustrate the distribution of data points. Each edge of the triangle corresponds to the ratio of one of the three ligands, with points within the triangle representing various combinations of these ligand ratios. A detailed explanation of the dimensionality reduction process is available in Section 1.5 of the Supporting Information. In Figure 3, the volume of POM–(NH_2_)_2_ is fixed at 1 mL, while the dispensed volumes for L1, L2, and L3 can range from 0 to 2 mL. In addition, chemical space maps with the dispensed volume of POM–(NH_2_)_2_ ranging from 0 to 2 mL are shown in Figures S2–S5. The color bar, which ranges from 0 to 1, signifies the probability of obtaining a single crystal of the target POMOF. As shown in Figure 3a, the probability of obtaining POMOF single crystals exceeds 0.7 across the majority of the three ligand ratio combinations. This high crystallization tendency can be attributed to the carefully chosen concentrations of reactants that were screened during the initial stages of the experiments. As for the possibility of obtaining the POMOF1 single crystals, Figure 3b indicates that the low L2 ratio, <0.2, would not have a negative effect on POMOF1 crystallization. Furthermore, we observed via microscope photography (Figure S14) that adding L1 with a small amount of L2 can actually help obtain single crystals of POMOF1 with uniform size and morphology on the micrometer scale. Similar phenomena were observed when obtaining POMOF3 single crystals. The addition of a small quantity of L1 or L2 during the POMOF3 synthesis can affect the size and shape of POMOF3 crystals (Figures S18, S19, and S21). By contrast, mixing L1 or L3 with L2 has a very low possibility, below 0.1, for obtaining POMOF2 single crystals (Figure 3c). A crucial observation arising from the chemical space maps of POMOF1 and POMOF3 is that they show overlap in the area, (Figure 3b,d) which indicates a high possibility of obtaining both POMOF1 and POMOF3 single crystals. In reality, experimental results indicate that POMOF1, POMOF3, and the mixed ligand POMOF1+3 all form in this area (Table S21). The topological structures of all POMOFs are listed in Figure 4.

### Robotic Synthesis of POMOFs Driven by _Χ_DL

Hardware-independent synthetic procedures in the standard format represented by the universal chemical description language (χDL) have been used to describe the reliable synthesis of organic molecules and nanoparticles.^15,27^ In this work, the unique digital signatures for POMOF synthesis were created in χDL format (Figure S33). The χDL for POMOFs synthesis recorded the name and concentration of reagents, the order and time of reagent addition, and the temperature and time of heating. See Supporting Information Section 2.5 for a complete description of the implementation. As shown in Table S56, the SC-XRD results and yields with the sample standard deviation ranging from 0.15 to 3.77 mg match well with the reactions, which were repeated three times, indicating the high reproducibility of the synthesis of POMOFs by this robotic platform. The products of the repeat reactions were used to collect additional characterization data.

### Crystal Structure

During the first stage of optimization, POMOF1–1, POMOF1–2, POMOF2–1, POMOF2–3, POMOF3–1, POMOF3–2, and POMOF1+3 were discovered. In the second stage, POMOFs with L2 ligands were the focus, leading to the discovery of POMOF2–2 and POMOF3–3. In total, there were 9 POMOFs discovered by this robotic platform with the XGboost algorithm.

As shown in Figure 4, a Tris-Mn-AE cluster functionalized with pyridyl groups as ligands can coordinate to Zn^2+^ nodes in two ways: one by pyridyl groups indicated by thick cyan sticks and the other by POM-based bridging oxygen atoms indicated by thick red-colored sticks. This scheme simplifies POMOF networks and is consistently used in all following structure descriptions.

Among these topological structures, the network of POMOF1–1 has a cubic structure similar to zeolite A type (the green-colored image on the right-hand side for the POMOF1–1 in Figure 4) forming large cavities and holding the solvated Zn–DMF complexes. The structure appears to have high porosity, but current N_2_ adsorption isotherm experiments have not determined the porosity of activated POMOF1–1 (Figure S60). This may be attributed to the high stability of the isolated solvated Zn–DMF complex, which is formed by additional Zn^2+^ ions and DMF ligands in the cavities.

This solvated Zn–DMF complex balances the charge for the 3-dimensional network of POMOF1–1 with a Mn:Zn molar ratio of 1 (Mn representing the AE cluster, see Table S61). In the 3-dimensional network, each AE cluster acts as a 4-connection node to link Zn ions through coordination of two nitrogen atoms from the two pyridyl groups and two oxygen atoms from the cluster. Each Zn also acts as a 4-connection node, binding two pyridyl groups and two oxygen atoms from two different clusters. A 4-connection node of the same type can also be found in POMOF3–1. POMOF3–1 has a Mn:Zn molar ratio of 1:2 other than 2:3 with some extra Zn centers coordinating to the imine N atom and the deprotonated phenol group (see Table S61). This Zn does not form nodes in the POMOF network and, therefore, is omitted in Figure 4 for POMOF3–1.

POMOF1–2 and POMOF3–2 have the same three-dimensional topological structure. POMOF1–2 has a Mn:Zn molar ratio of 2:3 and forms a 3D network of Mn as 4-connection nodes. There are two types of Zn centers in the structure. One type of Zn center forms 2-connected nodes, only coordinating two oxygen atoms from a cluster without pyridyl ligand coordination. The other type forms 3-connected nodes with one link to oxygen and two links from pyridyl coordination. POMOF3–2 has a Mn:Zn molar ratio of 2:3 with a tiny Zn excess coordinating to imine and deprotonated phenol groups. It should be noted that mixed ligands POMOF1+3 and POMOF3–3 have the same 3-D topological structure with Mn and Zn forming 4-connection nodes. They both have a Mn:Zn molar ratio of 1:1 with charge balanced by a TBA cation. The similar geometry of L1 and L3 not only leads to similar topologies for POMOF1–2 and POMOF3–2 but also allows the formation of POMOF1+3, which contains a mix of ligands L1 and L3.

The POMOF2 structures that were discovered have different topologies compared to the POMOF1 and POMO3 structures. POMOF2–1 has a Mn:Zn molar ratio of 1:2 with the charge balanced by an OH– ligand from H_2_O on Zn centers. The OH– ligand is disordered, with DMF molecules acting as ligands. Evidences show that the DMF ligands have lower occupancies of the dimethyl amine parts than usual. Mn forms 4-connection nodes, while Zn forms 2-connected nodes, and therefore, a two-dimensional layer structure is observed for POMOF2–1. POMOF2–2 has a Mn:Znmolar ratio of 1:1 with the charge balanced by a TBA cation and forms a unique 3D network. There is only one type of Zn center forming a 3-connected node, while there are two types of Mn nodes. One type is 2-connected, purely using the two pyridyl groups coordinating to Zn. The other type is a normal 4-connection node consisting of two pyridyl groups and two oxygen atoms coordinating to Zn. POMOF2–3 has a Mn:Zn molar ratio of 2:3 with the charge balanced. All Zn ions are within the formal POMOF network. There are two types of Zn nodes. One is 3-connected with 3-fold coordination consisting of two pyridyl ligands and one oxygen atom from a cluster; the other is 2-connected with two oxygens coordinating from clusters. The latter has a terminal ligand of free pyridine-3-carbaldehyde. All of the remaining terminal ligands on both Zn ions are DMF.

### Electrochemical Properties

To examine the electrochemical properties of the synthesized POMOFs, cyclic voltammetry (CV) measurements were conducted in the solid state using a three-electrode setup in an aqueous solution (pH = 2.9) saturated with N_2_. The results obtained from the selected POMOFs were then compared with those of POM–(NH_2_)_2_ and POM–Zn chains that only connected by Zn^2+^ under identical conditions. The shapes of the CV curves of all of the obtained POMOF electrodes with Faradaic capacitive characteristics show reversible multiple pairs of redox peaks. As illustrated in Figure S63, all of the POMOF samples exhibited similar redox features: one broad peak at positive potential in the range of 0.30 and 0.41 V (vs Ag/AgCl) paired with its corresponding cathodic peak at negative potential at around −0.2 V (vs Ag/AgCl). This is indicative that the POMOF materials display favorable reversibility for redox processes, involving oxidation of Mn species (from Mn^II^ to Mn^III^).^28^ Overall, the anodic–cathodic peak separation (Δ*E*_P_) is much smaller for the POMOFs than the one observed for bare POM–(NH_2_)_2_ and slightly smaller than the POM–Zn chain, suggesting the more efficient electron transfer and better redox reversibility in the POMOFs.^29^ This Δ*E*_P_ of each material is established by the position of the broadened anodic peak (*E*_a_), as shown in Figure S63. The values of the Δ*E*_P_ and *E*_a_ for all the POMOF materials are shown in Table 1. Additionally, a significant correlation exists between Δ*E*_P_ (or *E*_a_ position) and the ligand in the structure of a POMOF. POMOFs with L3 as their ligands exhibit superior electron transfer compared with those with L1 and much more than L2, implying that this could have an effect on the capacitance of the system. To further support this hypothesis, electrochemical impedance spectroscopy (EIS) measurements (Figure S64) confirm this trend, showing that the solution resistance (Rs) varies consistently with the ligand present on the POMOF. Meanwhile, the pristine POM and the POM–Zn complexes exhibit significantly higher resistance than POMOFs.^1^ In Figure S65, variation of the current at different scan rates is depicted. It can be observed that there are minimal changes in peak separation with the variation of the scan rate for the POMOFs. There is a significant increase in current intensity with increasing scan rates. This behavior indicates high-rate capability and suggests good electron transfer kinetics.

**Table 1:** Summary of Electrochemical Properties of POMOFs, POM–Zn, and POM–(NH_2_)_2_^a^

Sample	wt % POM (ICP)	wt % Zn (ICP)	Mn:Zn total molar ratio	Δ*E*_p_ Mn^II/III^ (V vs Ag/AgCl)	*E*_a_ Mn^II/III^ (V vs Ag/AgCl)	Capacitance (F/g_POM_) 10 cycles
POMOF1–1	43.83	4.37	1:1	0.55	0.40	58.45
POMOF2–1	29.31	3.97	1:2	0.49	0.43	40.80
POMOF2–3	48.09	5.42	2:3	0.57	0.41	36.39
**POMOF3–1**	**42.88**	**5.56**	**1:1**	**0.53**	**0.29**	**142.84**
POMOF3–2	45.02	3.43	2:3	0.49	0.32	97.17
POMOF1+3	47.89	3.33	1:1	0.52	0.34	87.43
POM–Zn	43.44	4.72	2:3	0.58	0.41	55.54
POM–(NH_2_)_2_	50.82	-	-	0.75	0.50	140.20

a POM: MnMo_6_O_18_.

The capacitance of the materials was investigated in order to compare the ability of energy storage between POMOF structures, which is indicative of the degree of electron transfer and electronic connectivity in the frameworks. To do that, galvanostatic charge–discharge was performed at an applied current density of 1 A/g of the sample for 10 cycles. Figure S66 shows the 10th cycle charge–discharge curves of the samples, and the capacitance values are included in Table 1, which were obtained from the discharge curve and normalized to the mass of an Anderson–Evans POM (MnMo_6_O_18_) in each POMOF. The weight ratio of POM in samples was calculated based on the relative Mo weight ratio measured by inductively coupled plasma (ICP) analysis. The weight ratio of Zn shown in Table 1 was also measured by ICP. The Mn:Zn total molar ratio shown in Figure 1 was calculated based on SC-XRD data.

As can be observed in Table 1, the capacitance of POMOF3–1 is much higher than the POM–Zn and all other POMOF materials; however, it is only slightly higher than the capacitance of a pure POM–(NH_2_)_2_. The capacitance obtained after 10 cycles for POMOF3 systems was consistent with the increasing weight ratio of Zn ions in POMOFs, following the trend POMOF3–1 (142.84 F/g_POM_) > POMOF3–2 (97.17 F/g_POM_) > POMOF1+3 (87.43 F/g_POM_). Moreover, the capacitance of POMOF3–1 is around 2.6 times higher than that of the POM–Zn chain, whose loading of Zn is 85% of that of the POMOF3–1.

Consequently, POMOFs with L3 ligands were found to exhibit both superior electron transfer and capacitance, which could be attributed to the presence of deprotonated phenol groups coordinating Zn ions characteristic of the molecular structure of such ligands. This insight opens up new avenues for future research where the systematic modification of ligands could be explored as a strategy to optimize electron transfer properties and capacitance in these materials.

## Conclusion

We have developed a hybrid algorithm-driven robotic system that uses the XGBoost algorithm for the closed-loop exploration of the crystallization boundaries of POMOFs, within the synthesis platform. After building the initial model based on the initial data, the XGBoost model was optimized using iteratively updated data obtained by uncertainty feedback experiments. To effectively improve the accuracy of the model, based on the POMOF classification from their chemical constitution, the binary classification was extended into a multiclass classification. The final models achieve F1 scores of 0.85 (all), 0.82 (POMOF1), 0.85 (POMOF2), and 0.89 (POMOF3) for each system. The predicted results from these models match well with the actual experimental results with a high accuracy around 0.8 and above. In order to visualize the crystallization propensity of POMOFs with factors in 6 dimensions and guide further synthesis, chemical space maps in triangular plots were plotted based on the XGBoost model with dimensionality reduction. In addition, the POMOF syntheses, driven by the unique digital signatures represented by χDL, have achieved reproducible crystallization and yields. Among the 9 newly discovered POMOFs in 2D and 3D structures, POMOF1–1 has an interesting cubic structure similar to the zeolite A type, and POMOF1+3 is formed with mixed ligands of L1 and L3. Furthermore, the electrochemical properties of the synthesized POMOFs were investigated by CV measurements to compare their electron transfer and energy storage abilities. POMOF3–1 with the highest Zn loading exhibited superior electron transfer compared to pure POM–(NH_2_)_2_ and achieved the highest capacitance after 10 cycles compared to not only the POMOFs formed by L1 and L2 but also other POMOF3s. These results indicated that the ratio of Zn, the type of ligands used, and the topological structures of POMOFs directly affect their electrochemical properties, which will be further explored in future work.

## Supporting Information Available

Experimental details and synthetic procedures; Figures S1–S66: photographs of the platform, additional chemical space maps, the χDL snippet, the accuracy of models, microscope photos, PXRD patterns, FT-IR spectra, TGA plots, gas sorption analysis data, and electrochemical properties; Tables S1–S62: experimental conditions and phases for the products, SC-XRD refinement details, ICP and elemental analysis data (PDF)
