# Supplementary material for: Algorithm-Driven Robotic Discovery of Polyoxometalate-Scaffolding Metal–Organic Frameworks
Source: J Am Chem Soc. 2024 Oct 9;146(42):28952–60. doi: 10.1021/jacs.4c09553 (PMC11503775; doi:10.1021/jacs.4c09553)
Supplement: Supplementary file 1 — ja4c09553_si_001.pdf [file ja4c09553_si_001.pdf]

Supplementary Information for:

Algorithm-Driven Robotic Discovery of Polyoxometalate-Scaffolding Metal-Organic Frameworks

Donglin He,[†] Yibin Jiang,[†] Melanie Guillén-Soler,[†] Zack Geary,[†] Lucia Vizcaíno-Anaya,[†,‡] Daniel Salley,[†] Maria del Carmen Gimenez-Lopez, [‡] De-Liang Long,[*,†] Leroy Cronin[*,†]

[†]School of Chemistry, University of Glasgow, University Avenue, Glasgow G12 8QQ, United Kingdom. Email: deliang.long@glasgow.ac.uk; lee.cronin@glasgow.ac.uk.

[‡]Centro Singular de Investigación en Química Biolóxica e Materiais Moleculares (CiQUS), Universidade de Santiago de Compostela, Santiago de Compostela, 15782, Spain.

Contents

1. Experiment details 3
1.1 Materials and characterizations 3
1.2 Electrochemical measurements 4
1.3 Robotic platform 5
1.4 XGBoost 6
1.5 Dimensionality reduction for the chemical space plot 8
2. Synthetic procedures 13
2.1 Screen the proper feeding sequence and concentration 13
2.2 Generate initial experiment design randomly 25
2.3 Initial experiment design by chemists 26
2.4 Feedback optimization experiment 28
2.5 The unique digital signature for the synthesis of POMOFs 51
3. XGBoost accuracy 55
4. Characterization 61
4.1 SC-XRD data 61
4.2 ICP and elemental analysis data 66
4.3 PXRD 67
4.4 IR spectra 78
4.5 TGA data 82
4.6 Gas sorption 83
4.7 Electrochemical properties 86
References 90

1. Experiment details

1.1 Materials and characterizations

The δ-Tris-based Mn-Anderson (POM-$(NH_2)_2$, $[N(C_4H_9)_4]_3[MnMo_6O_{18}\{(OCH_2)_3CNH_2\}_2]$) was synthesized as described previously.[1] 4-pyridinecarboxaldehyde (L1), 3-pyridinecarboxaldehyde (L2) and 3-hydroxypyridine-4-carboxaldehyde (L3) were purchased from Fluorochem and used as received. All the solvent were purchased from Honeywell.

Elemental analysis

The contents of carbon, hydrogen and nitrogen were determined using an EA 1110 CHNS CE440 Elemental Analyzer, which belongs to the chemistry microanalysis services in the University of Glasgow.

Inductivity-coupled plasma analysis

The contents of manganese, molybdenum and zinc was analyzed by inductivity-coupled plasma (ICP) performed on Agilent 5100 SVDV ICP-OES. Samples around 5-7 mg were digested in a mixture of 2 mL deionized water, 3 mL 10wt% HNO_3 and 1 mL H_2O_2 by heating, then cooled down to the room temperature and diluted to 50 ml.

IR Spectroscopy

The FT-IR spectrum was collected using a JASCO FT-IR 4100 spectrometer in the range of 400 - 4000 cm^{-1}. Wavenumbers are given in cm^{-1}.

Thermogravimetric analysis

The instrument for the thermogravimetric analysis (TGA) is a Discovery TGA 5500, manufactured by TA Instruments. Samples were run from room temperature to 800 °C at 10 °C min^{-1} in 25 mL min^{-1} nitrogen.

Gas sorption

N_2 isotherms of POMOF1-1, POMOF2-3 and POMOF3-1 were collected at 77 K using a Micromeritics 3flex surface characterization analyzer. Samples were desolvated by solvent exchange with methanol, and dried in vacuum at 120 °C for 12 h. Samples were degassed at 100 °C for 15 hours under dynamic vacuum prior to gas analysis.

Powder X-ray diffraction

Samples were loaded into borosilicate glass capillaries, and powder X-ray diffraction (PXRD) patterns were recorded from 3.5 - 30° in transmission mode on a PANalytical X'Pert PRO diffractometer at room temperature, equipped with a sample spinner, X-ray focusing mirror and PIXcel^{3D} detector, using Cu-Kα (λ = 1.54 Å) radiation.

Single crystal X-ray diffraction

The single crystal was quickly picked up from the solution of the test tube, wrapped with Paraba oil and mounted onto a MiTeGen rubber loop. Some of the single-crystal datasets and unit cells for the compounds were collected at 150(2) K on a Rigaku XtaLAB Synergy R HyPix-Arc 150 diffractometer equipped with a graphite monochromator (Mo-Kα radiation, λ = 0.71073 Å) on a micro-focus sealed X-ray source (50 kV, 24 mA). Data collection and reduction were performed using the CrysAlisPro software package. The remaining datasets were collected at Beamline I19 of Diamond Light Source UK using silicon double crystal monochromatic synchrotron radiation (λ = 0.6889 Å, Dectris PILATUS 2M detector) with ω and ψ scans at 100(2) K. Data integration and reduction were undertaken with Xia2. Structures were solved by direct methods and refined against F^2 by full matrix least squares. Most non-hydrogen atoms, except disordered atoms, were refined anisotropically. Hydrogen atoms were generated geometrically. All calculations were performed using the SHELX-2019 program package with WinGX suite. The X-ray crystallographic data in this manuscript have been deposited at the Crystallographic Data Centres with CCDC numbers 2353739-2353749. The data can be obtained free of charge from Cambridge Crystallographic Data Centre service www.ccdc.cam.ac.uk/structures with deposition number.

1.2 Electrochemical measurements

Working electrode preparation

To prepare the working electrode, a dispersion of 2 mg each of the samples after dried in vacuum at RT for 12 h and 2 mg of carbon black (Vulcan X-72) in 2 mL of water was used as an ink. Thereafter, 10 μL of the obtained highly dispersed catalyst ink was dropped onto a mirror polished glassy carbon electrode (GCE) of 0.07 cm^2 of geometric surface area. Subsequently, 10 uL of Nafion solution (5 wt%) was added and the electrode was dried under ambient conditions.

Electrochemical characterization set-up

An Autolab potentiostat PGSTAT204 was used to measure the electrochemical characterization for all the POMOFs. All the electrochemical measurements were conducted in a three-electrode set-up, with a GCE, Ag/AgCl (3M KCl) and a platinum wire as working, reference and counter electrode, respectively. The measurements were carried out under N_2 atmosphere in an aqueous electrolyte solution of 0.5 mM H_2SO_4 + 2.5 mM Na_2SO_4 at constant pH = 2.9.

Electrochemical measurements

Cyclic Voltammetry (CV) measurements under N_2-saturated 0.5 mM H_2SO_4 + 2.5 mM Na_2SO_4 aqueous solution were performed to characterize the materials. Current densities were normalized to the electrode geometrical area and the mass of POM ($MnMo_6O_{18}$).

Galvanostatic charge-discharge measurements (CD)

To obtain the capacitance value (C) of the materials, galvanostatic charge-discharge was performed for 10 cycles applying constant current (i) of 1 (and -1) A/g_{sample} in the charge (and discharge) processes in a fixed potential window (ΔV) of 0.8 and -0.8 V vs Ag/AgCl. The capacitances are calculated based on the charge or discharge time (t) as follows:

$$C\ [F/g_{POM}] = \frac{i * t}{\Delta V * g_{POM}} = \frac{1\ [A/g_{sample}] * t\ [s]}{1.6\ [V] * wt\%POM}$$

1.3 Robotic platform

The core robotic hardware shown in Figure 1b consists of a chemical reaction module capable of performing parallel synthesis up to 24 reactors and a heating mantle for 48 reactions (14 mL vials).[2] By using the rotation of the Geneva wheel and high-precision syringe pumps, the chemical reaction module performs liquid handling to achieve highly accurate control of the adding volume of reactant solutions. POM-$(NH_2)_2$, L1, L2, L3 and $Zn(NO_3)_2 \cdot 6H_2O$ were dissolved in *N*,*N*-dimethylformamide (DMF) separately as stock solutions. To control the concentration of reactants finely, the DMF was also used as the stock solution. The adding volumes of these 6 stock solutions are both from 0 to 2 mL. After reagents adding, the vials were sealed and transferred manually to the heating mantle, and heat temperature was set up in 80 °C (The real temperature in the solution is 65 ± 3°C). All the vials were left to stand for 48

h without stirring, and then the images of each vial were obtained by the camera installed on a microscope.

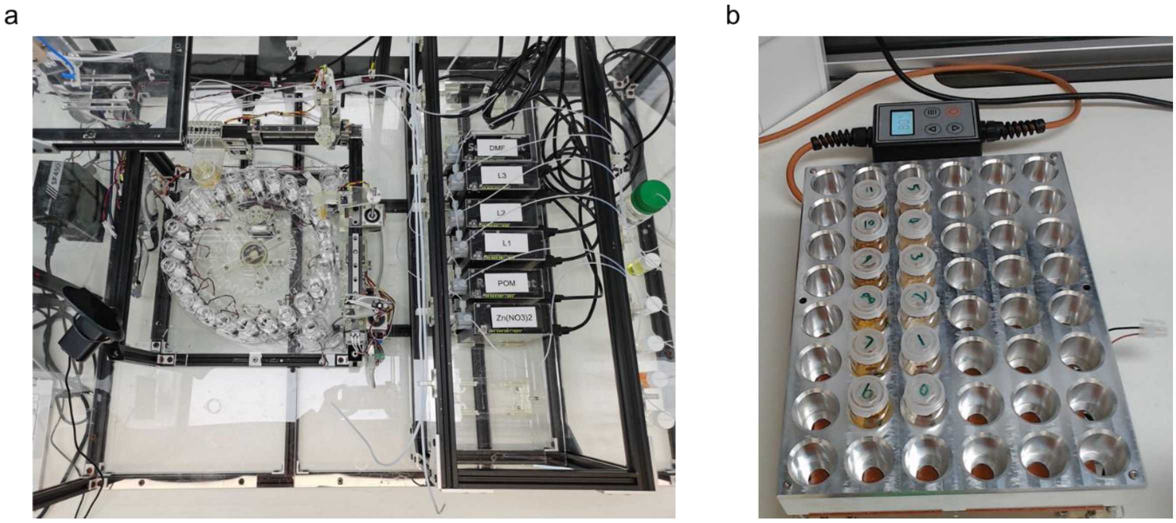

Figure S1: Real image of the platform used for this work (a) The chemical reaction module, (b) The vial heater.

1.4 XGBoost

After the initial experiments, further exploration of the chemical space was guided by model uncertainty to enhance the understanding of the crystallization boundaries. Here, we trained a binary classifier using the extreme gradient boosting (XGBoost) method using the existing data. If the experimental condition can lead to target single crystals, it is labelled as 1, otherwise 0. During the training, 20% of the data was used as the validation set and 80% as the training set. Before the training process, the input variables are normalized to the range between 0 and 1. To optimize the hyperparameters, k-fold cross-validation with recall as the score was used, where we set k as 4 if not mentioned otherwise.

After the selection of hyperparameters and training, for any input $\boldsymbol{x}$, the model can predict the probability of crystallization P_x under this experimental condition. Hence, the uncertainty of the prediction can be estimated as $f = -|P_x - 0.5|$ for the binary classifier. When the model is more uncertain, P_x will approximate 0.5.

During the sampling process, we created grids from 0 to 1 with an interval of 0.1 in each dimension, which are the potential experimental conditions. For N-dimensional input, the number of potential experimental conditions is 11^N. Their uncertainty can be evaluated using

the model. However, directly sampling points with high f values is not ideal because these points tend to cluster in certain regions. Besides the uncertainty, we also need to consider the sampling diversity in the chemical space. Thus, each dimension of the input variable is segmented into subregions of $[0, 0.25)$, $[0.25, 0.5)$, $[0.5, 0.75)$, $[0.75, 1]$, which segments the N-dimensional hypercube into 4^N sub-hypercubes. For each sub-hypercube, the uncertainty of the experimental conditions within it will be evaluated, and the condition with the highest f value will be selected. By iterating through all the sub-hypercubes, 4^N experimental conditions representing the local highest uncertainty will be generated. Then from this point set, we selected 10 points with the highest uncertainty as the experiments to be performed. Once the experiments are finished, the new conditions as well as the corresponding results will be added to the previous data set to update the model and re-generate new conditions. This process iterated until the exploration is finished.

The accuracy of the model including precision (P), recall (R), F1 score (F1) and prediction (PD) are calculated to evaluate the performance of machine learning models and shown in Equations 1 – 4.

$$P = \frac{TP}{TP + FP} \qquad (1)$$

$$R = \frac{TP}{TP + FN} \qquad (2)$$

$$F1 = 2 \times \frac{P \times R}{P + R} \qquad (3)$$

As for the validation set (20% of the data in this work for training the model). TP, FP, TN, and FN represent the number of true positives, the number of false positives, the number of true negatives, and the number of false negatives. F1 score represents the harmonic mean of precision and recall.

$$PD = \frac{PR}{TE} \qquad (4)$$

where, prediction (PD) is represents is the ratio of the number of correctly predicted results (PR) in a set compared with the total number of experiments (TE) in said set shown in Table S54. The PD is calculated for the comparison of out-of-sample prediction results made by the XGBoost model with the actual reaction outcomes.

1.5 Dimensionality reduction for the chemical space plot

In order to create visual representations of data with 6 dimensions, chemical space maps based on the model for POMOFx, POMOF1, POMOF2 and POMOF3 were plotted by dimensionality reduction.

The adding volumes of reagents are both from 0 to 2 ml. Grids were created from 0 to 1 with an interval of 0.1 in each dimension, whose double value is the potential experimental conditions. As for 6-dimensional input, the number of potential experimental conditions is 11^6. We calculated the predicted results of the potential experimental conditions based on the XGBoost models. We fixed the adding volume of POM–$(NH_2)_2$ into 0, 0.2, 0.4, 0.6, 0.8, 1.0, 1.2, 1.4, 1.6, 1.8 and 2.0, respectively. Then the adding volumes of L1, L2, and L3 were represented by the ratio from 0 to 1. Each corner of the triangle corresponds to the ratio of three ligands, with points within the triangle representing various combinations of these ligand ratios. After that, we calculated the ratio of the "1" result for the reaction conditions with the same adding volumes of L1, L2, and L3, which can be the possibility for obtaining the target POMOF single crystals, and was represented by the color bar. Figure 3 shows the fixed volume of POM–$(NH_2)_2$ of 1 ml, while the adding volumes for L1, L2, and L3 can range from 0 to 2 ml. The other chemical space maps with the adding volume of POM-$(NH_2)_2$ from 0 to 2 mL are shown in Figure S2-S5.

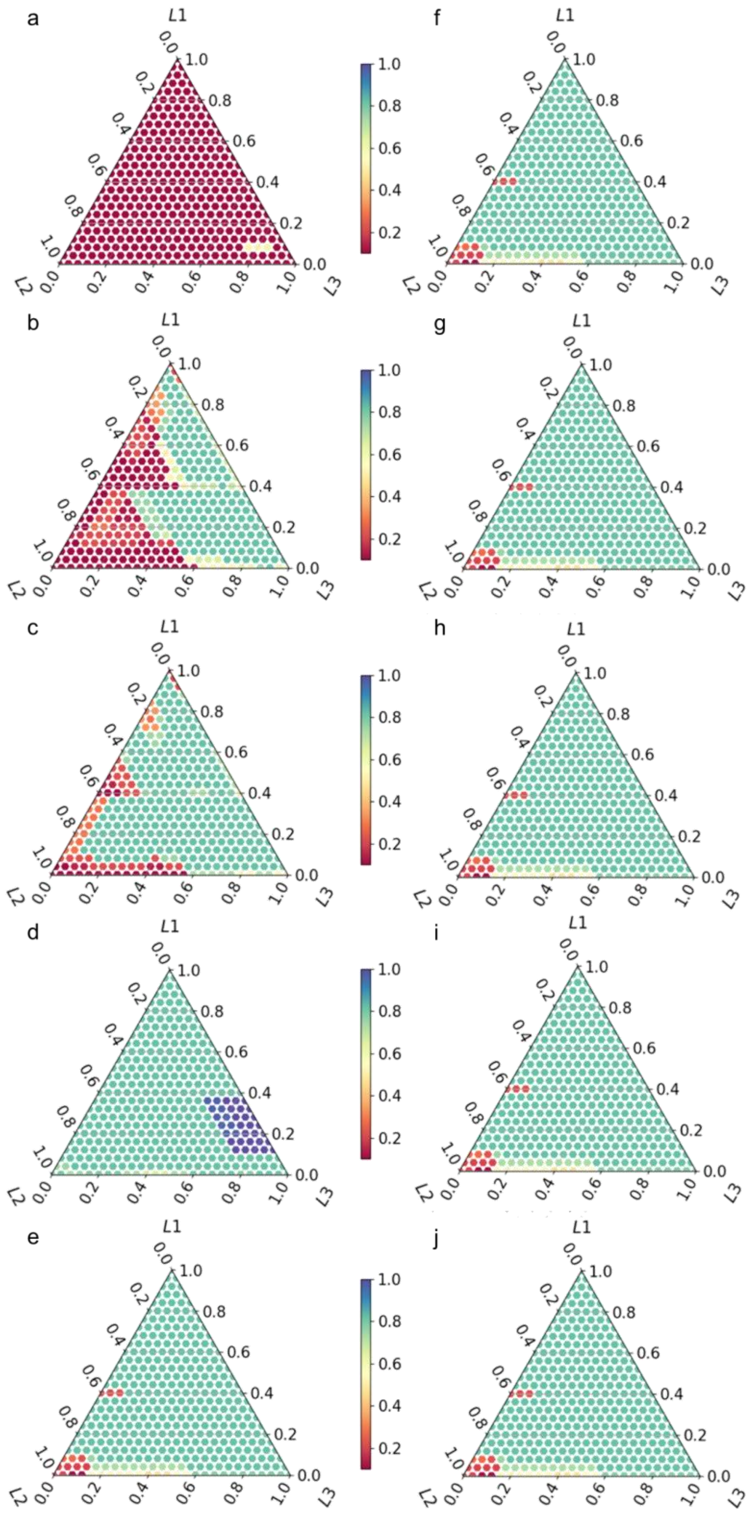

Figure S2: Chemical space maps predicted by the XGBoost for POMOFx whose adding volume for POM-$(NH_2)_2$ are (a) 0 mL, (b) 0.2 mL, (c) 0.4 mL, (d) 0.6 mL, (e) 0.8 mL, (f) 1.2 mL, (g) 1.4 mL, (h) 1.6 mL, (i) 1.8 mL and (j) 2.0 mL.

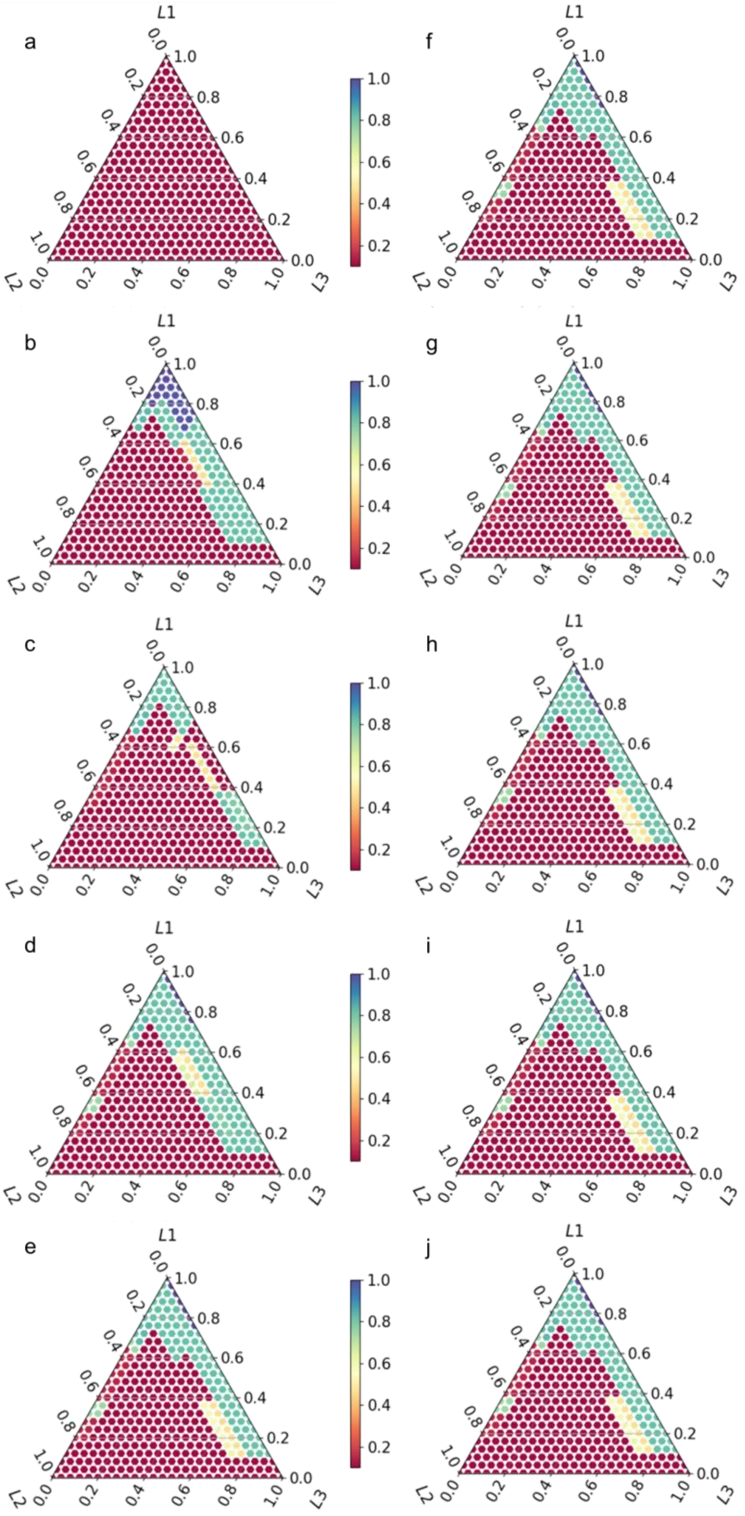

Figure S3: Chemical space maps predicted by the XGBoost for POMOF1 whose adding volume for POM-$(NH_2)_2$ are (a) 0 mL, (b) 0.2 mL, (c) 0.4 mL, (d) 0.6 mL, (e) 0.8 mL, (f) 1.2 mL, (g) 1.4 mL, (h) 1.6 mL, (i) 1.8 mL and (j) 2.0 mL.

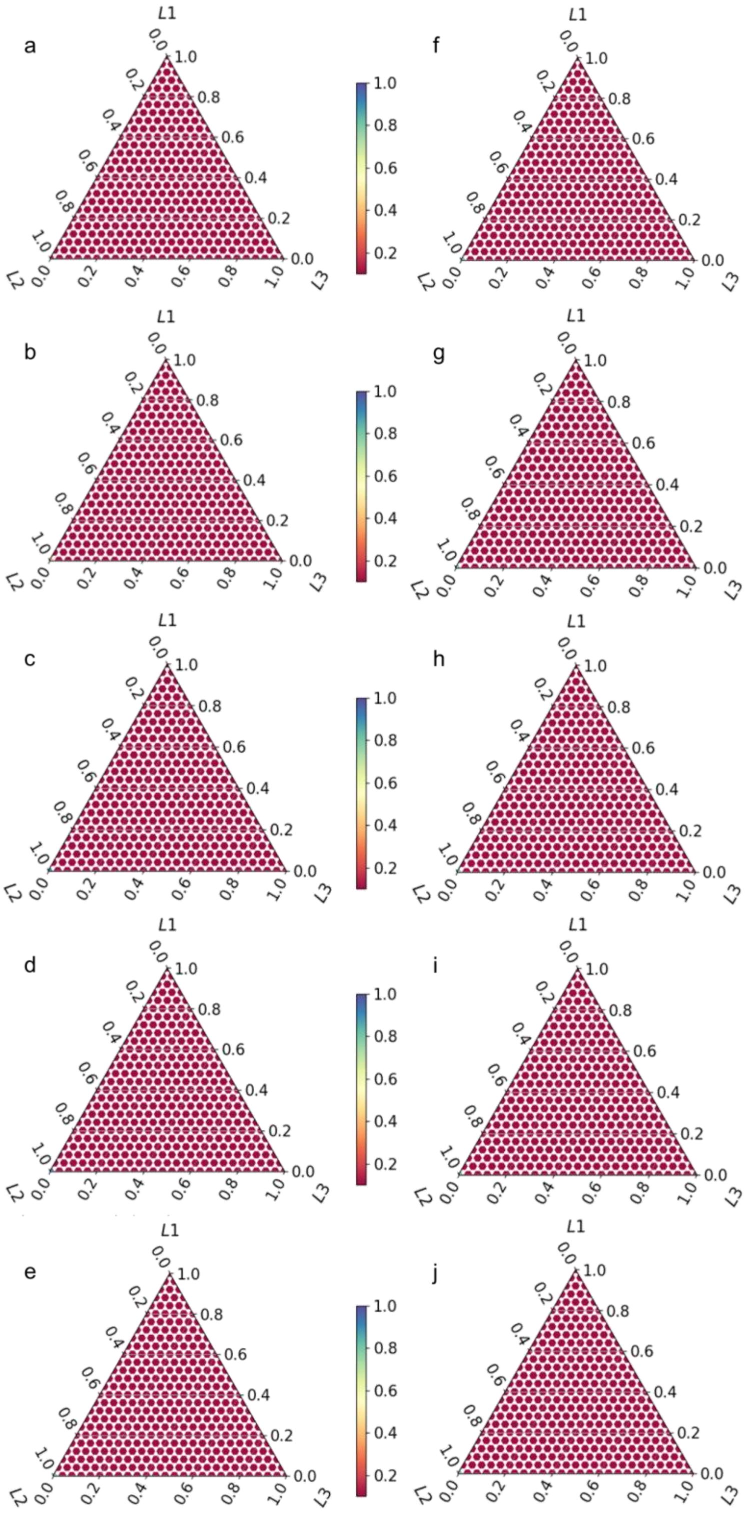

Figure S4: Chemical space maps predicted by the XGBoost for POMOF2 whose adding volume for POM-$(NH_2)_2$ are (a) 0 mL, (b) 0.2 mL, (c) 0.4 mL, (d) 0.6 mL, (e) 0.8 mL, (f) 1.2 mL, (g) 1.4 mL, (h) 1.6 mL, (i) 1.8 mL and (j) 2.0 mL.

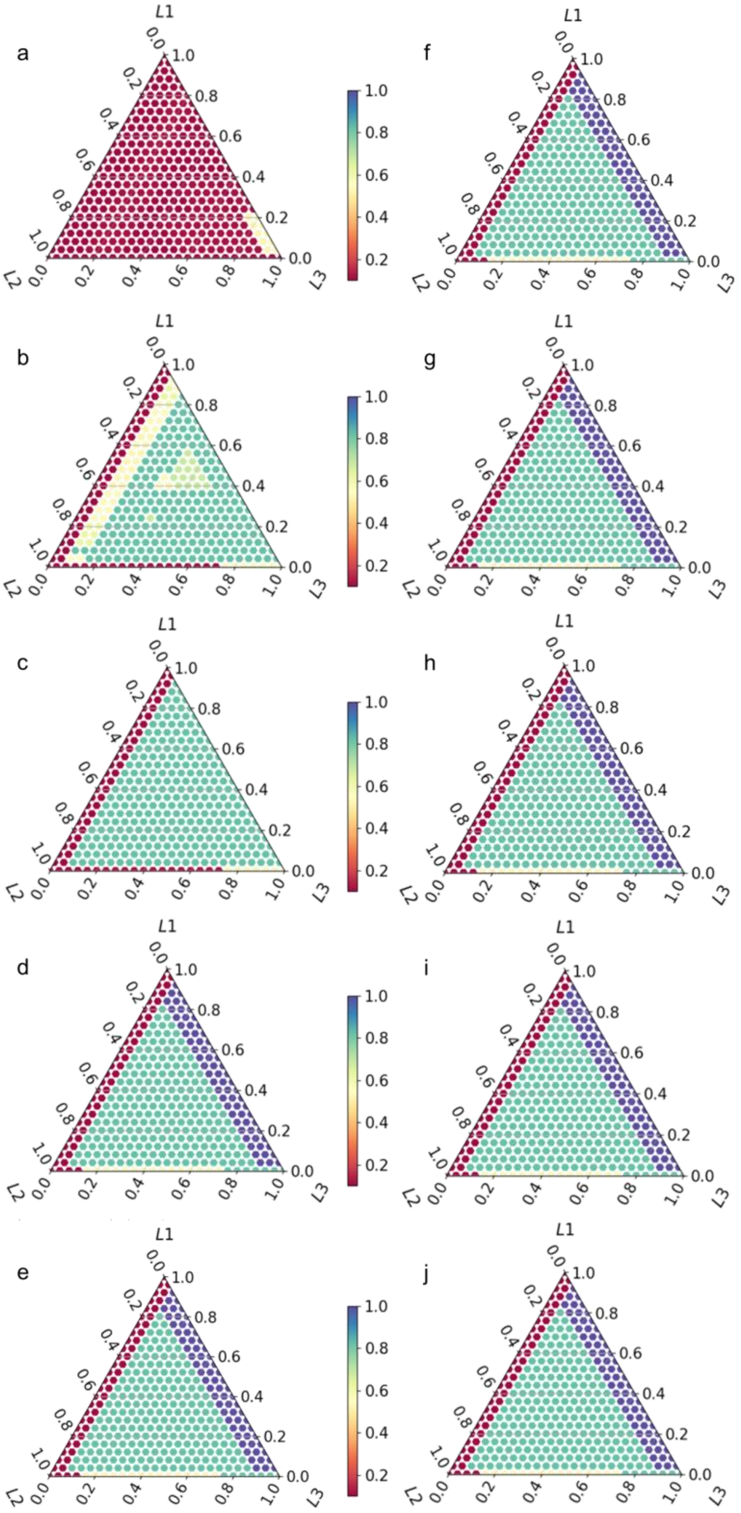

Figure S5: Chemical space maps predicted by the XGBoost for POMOF3 whose adding volume for POM-$(NH_2)_2$ are (a) 0 mL, (b) 0.2 mL, (c) 0.4 mL, (d) 0.6 mL, (e) 0.8 mL, (f) 1.2 mL, (g) 1.4 mL, (h) 1.6 mL, (i) 1.8 mL and (j) 2.0 mL.

2. Synthetic procedures

2.1 Screen the proper feeding sequence and concentration

Table S1: Summary of stock solutions in DMF prepared for the 1st and 2nd synthetic screen.

Reactant	MW (g/mol)	Stock solution concentration (mg/mL)	Stock solution concentration (mmol/ mL)	Total volume of stock solution made (mL)	Mass of reactant required for stock solution (mg)
POM-$(NH_2)_2$	1882.26	3.76	0.002	50	188
L1	107.11	0.43	0.004	50	21.5
L2	107.11	0.43	0.004	50	21.5
L3	123.11	0.49	0.004	50	24.5
$Zn(NO_3)_2·6H_2O$	297.49	0.89	0.003	100	89

Table S2: Summary of reactant solution volumes used for the 1st synthetic screen.

Reaction number	POM-$(NH_2)_2$ volume (mL)	L1 volume (mL)	L2 volume (mL)	L3 volume (mL)	$Zn(NO_3)_2·6H_2O$ volume (mL)	Molar ratio/ POM:L1:L2:L3:Zn	Single crystal? (Y/N)	Phase
1	2	2	0	0	2	1:2:0:0:1.5	Y	POM-Zn
2	1	2	0	0	1	1:4:0:0:1.5	N	-
3	2	1	0	0	2	1:1:0:0:1.5	Y	POM-Zn
4	2	0	2	0	2	1:0:2:0:1.5	Y	POM-Zn
5	1	0	2	0	1	1:0:4:0:1.5	Y	POM-Zn
6	2	0	1	0	2	1:0:1:0:1.5	Y	POM-Zn, POM-Zn'
7	2	0	0	2	2	1:0:0:2:1.5	N	-
8	1	0	0	2	1	1:0:0:4:1.5	N	-
9	2	0	0	1	2	1:0:0:1:1.5	N	-
10	2	1	1	0	2	1:1:1:0:1.5	Y	POM-Zn
11	2	1	0	1	2	1:1:0:1:1.5	N	-
12	2	0	1	1	2	1:0:1:1:1.5	N	-
13	2	0	0	2	3.3	1:0:0:2:2.5	N	-

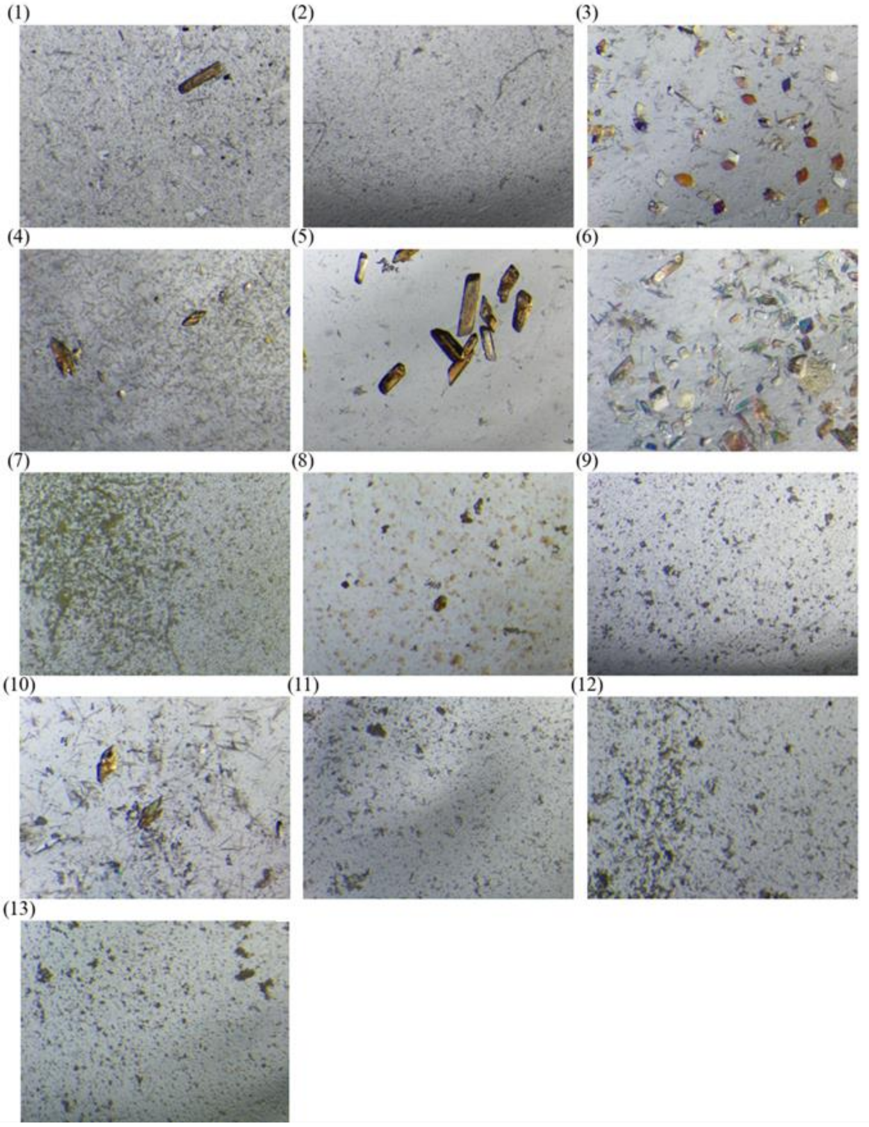

Figure S6: Microscope photos for the 1st synthetic screen.

Table S3: Summary of reactant solution volumes used for the 2nd synthetic screen.

**Reaction number**	**POM-$(NH_2)_2$ volume (mL)**	**L1 volume (mL)**	**L2 volume (mL)**	**L3 volume (mL)**	**$Zn(NO_3)_2 \cdot 6H_2O$ volume (mL)**	**Molar ratio/ POM:L1:L2:L3:Zn**	**Single crystal? (Y/N)**	**Phase**
1	2	2	0	0	2	1:2:0:0:1.5	N	-
2	1	2	0	0	1	1:4:0:0:1.5	N	-
3	2	1	0	0	2	1:1:0:0:1.5	Y	POM-Zn
4	2	0	2	0	2	1:0:2:0:1.5	Y	POM-Zn, POM-Zn’
5	1	0	2	0	1	1:0:4:0:1.5	N	-
6	2	0	1	0	2	1:0:1:0:1.5	Y	POM-Zn, POM-Zn’
7	2	0	0	2	2	1:0:0:2:1.5	N	-
8	1	0	0	2	1	1:0:0:4:1.5	N	-
9	2	0	0	1	2	1:0:0:1:1.5	N	-
10	2	1	1	0	2	1:1:1:0:1.5	Y	POM-Zn
11	2	1	0	1	2	1:1:0:1:1.5	N	-
12	2	0	1	1	2	1:0:1:1:1.5	N	-
13	2	0	0	2	3.3	1:0:0:2:2.5	N	-

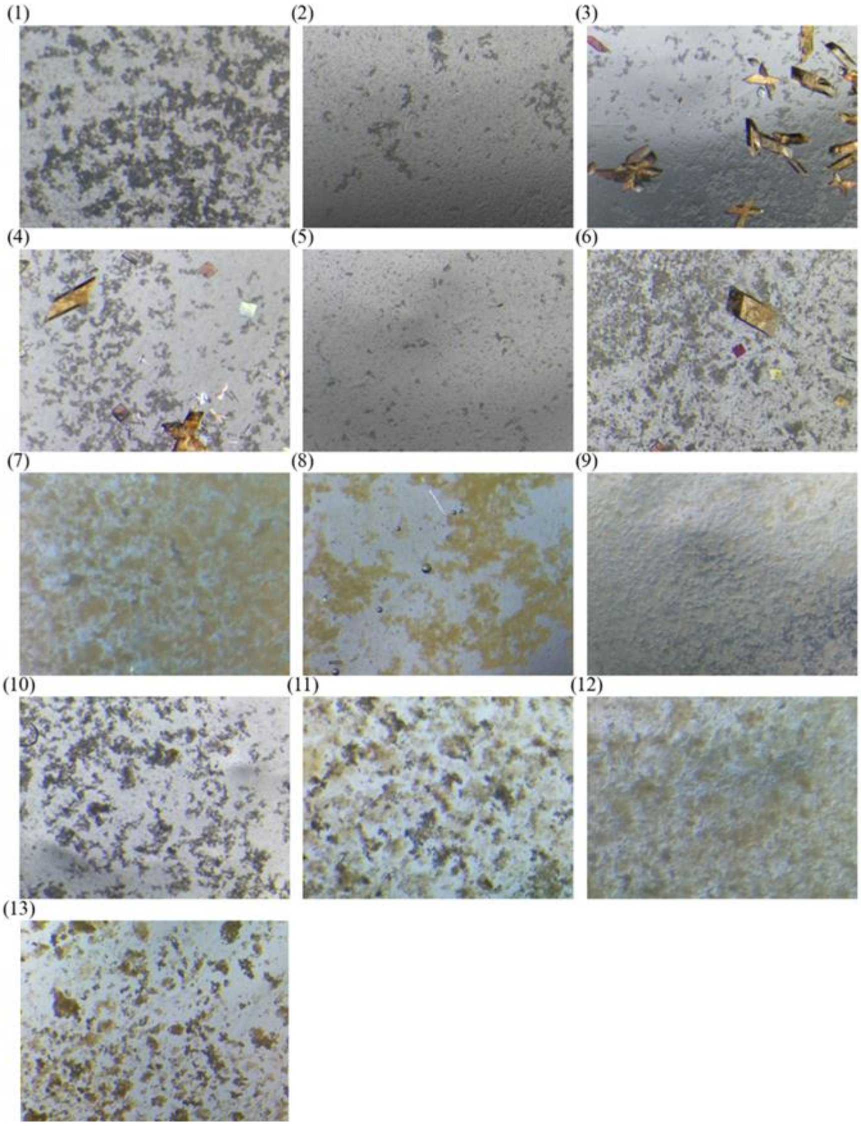

Figure S7: Microscope photos for the 2nd synthetic screen.

Table S4: Summary of stock solutions in DMF prepared for the 3rd synthetic screen.

Reactant	MW (g/mol)	Stock solution concentration (mg/mL)	Stock solution concentration (mmol/ mL)	Total volume of stock solution made (mL)	Mass of reactant required for stock solution (mg)
POM-$(NH_2)_2$	1882.26	11.29	0.006	50	564.5
L1	107.11	1.29	0.012	50	64.5
L2	107.11	1.29	0.012	50	64.5
L3	123.11	1.48	0.012	50	74
$Zn(NO_3)_2 \cdot 6H_2O$	297.49	1.79	0.006	100	179

Table S5: Summary of reactant solution volumes used for the 3rd synthetic screen.

Reaction number	POM-$(NH_2)_2$ volume (mL)	L1 volume (mL)	L2 volume (mL)	L3 volume (mL)	$Zn(NO_3)_2 \cdot 6H_2O$ volume (mL)	Molar ratio/ POM:L1:L2:L3:Zn	Single crystal? (Y/N)	Phase
1	2	0	0	0	3	1:0:0:0:1.5	Y	POM-Zn, POM-Zn’
2	0	2	0	0	3	0:1:0:0:0.75	N	-
3	0.5	2	0	0	3	1:8:0:0:6	Y	POMOF1-1, POMOF2-1
4	2	0.5	0	0	3	1:0.5:0:0:1.5	Y	POM-Zn, POM-Zn’
5	1	2	0	0	3	1:4:0:0:3	Y	POMOF1-1, POMOF2-1
6	2	2	0	0	3	1:2:0:0:1.5	Y	POM-Zn
7	2	1	0	0	3	1:1:0:0:1.5	Y	POM-Zn, POM-Zn’
8	0	0	2	0	3	0:0:1:0:0.75	N	-
9	0.5	0	2	0	3	1:0:8:0:6	Y	POM-Zn
10	2	0	0.5	0	3	1:0:0.5:0:1.5	Y	POM-Zn, POM-Zn’
11	1	0	2	0	3	1:0:4:0:3	Y	POM-Zn
12	2	0	2	0	3	1:0:2:0:1.5	Y	POM-Zn
13	2	0	1	0	3	1:0:1:0:1.5	Y	POM-Zn’
14	0	0	0	2	3	0:0:0:1:0.75	N	-
15	0.5	0	0	2	3	1:0:0:8:6	N	-
16	2	0	0	0.5	3	1:0:0:0.5:1.5	Y	POM-Zn’
17	1	0	0	2	3	1:0:0:4:3	Y	POMOF3-1
18	2	0	0	2	3	1:0:0:2:1.5	N	-
19	2	0	0	1	3	1:0:0:1:1.5	N	-

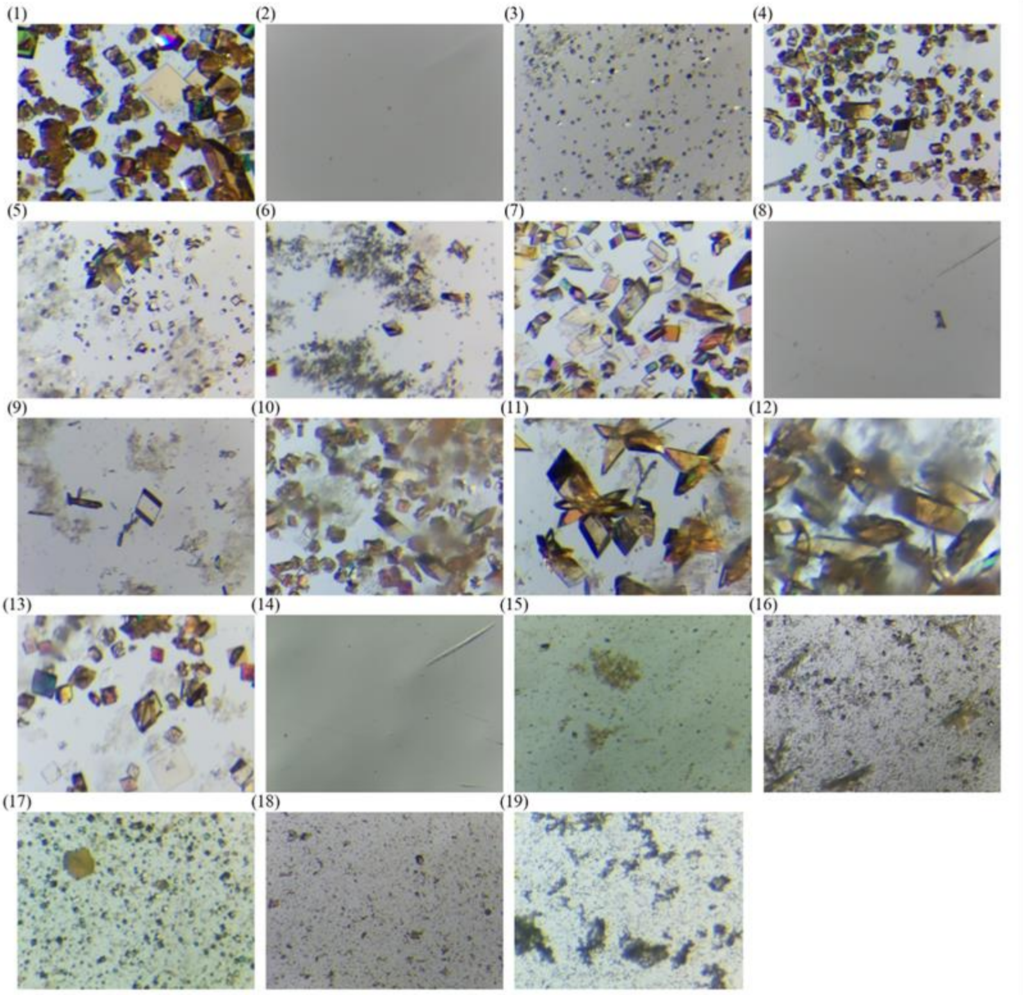

Figure S8: Microscope photos for the 3rd synthetic screen.

Table S6: Summary of stock solutions in DMF prepared for the 4th synthetic screen.

Reactant	MW (g/mol)	Stock solution concentration (mg/mL)	Stock solution concentration (mmol/ mL)	Total volume of stock solution made (mL)	Mass of reactant required for stock solution (mg)
POM-$(NH_2)_2$	1882.26	56.47	0.03	50	2823.5
L1	107.11	12.85	0.12	50	642.5
L2	107.11	12.85	0.12	50	642.5
L3	123.11	14.77	0.12	50	738.5
$Zn(NO_3)_2·6H_2O$	297.49	13.39	0.045	100	1339

Table S7: Summary of reactant solution volumes used for the 4th synthetic screen.

Reaction number	POM-$(NH_2)_2$ volume (mL)	L1 volume (mL)	L2 volume (mL)	L3 volume (mL)	$Zn(NO_3)_2·6H_2O$ volume (mL)	Molar ratio/ POM:L1:L2:L3:Zn	Single crystal? (Y/N)	Phase
1	1	0.5	0	0	1	1:2:0:0:1.5	Y	POMOF1-1
2	0.5	1	0	0	1	1:8:0:0:3	Y	POMOF1-1
3	1	0.2	0	0	1	1:0.8:0:0:1.5	N	-
4	0.5	1.5	0	0	1	1:12:0:0:3	N	-
5	1	0	0.5	0	1	1:0:2:0:1.5	N	-
6	0.5	0	1	0	1	1:0:8:0:3	Y	POMOF2-1
7	1	0	0.2	0	1	1:0:0.8:0:1.5	Y	POM-Zn
8	0.5	0	1.5	0	1	1:0:12:0:3	Y	POMOF2-1
9	1	0	0	0.5	1	1:0:0:2:1.5	N	-
10	0.5	0	0	1	1	1:0:0:8:3	Y	POMOF3-2
11	1	0	0	0.2	1	1:0:0:0.8:1.5	Y	POM-Zn
12	0.5	0	0	1.5	1	1:0:0:12:3	Y	POMOF3-2

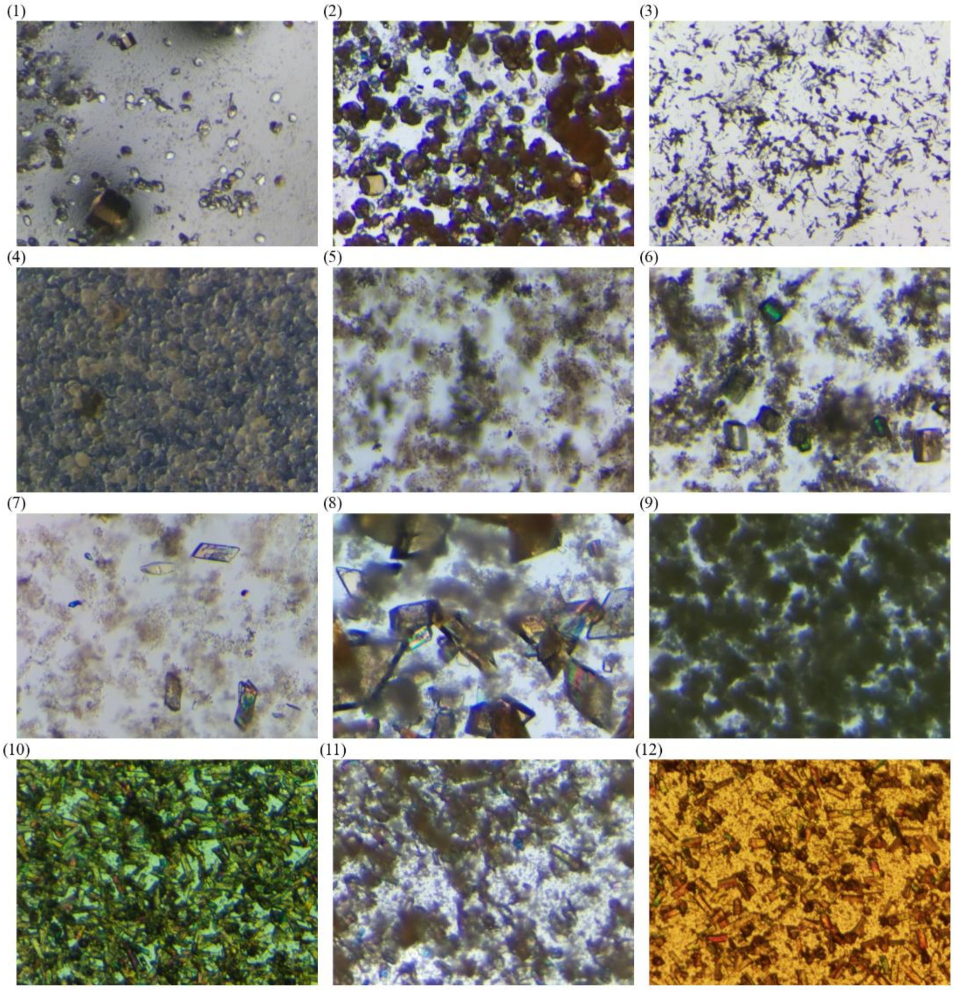

Figure S9: Microscope photos for the 4th synthetic screen.

Table S8: Summary of stock solutions in DMF prepared for the 5th synthetic screen.

**Reactant**	**MW (g/mol)**	**Stock solution concentration (mg/mL)**	**Stock solution concentration (mmol/ mL)**	**Total volume of stock solution made (mL)**	**Mass of reactant required for stock solution (mg)**
POM-$(NH_2)_2$	1882.26	28.24	0.015	25	706
L1	107.11	3.21	0.03	25	80.25
L2	107.11	3.7	0.03	25	92.5
L3	123.11	3.7	0.03	25	92.5
$Zn(NO_3)_2{\cdot}6H_2O$	297.49	6.7	0.0225	50	335

Table S9: Summary of reactant solution volumes used for the 5th synthetic screen.

**Reaction number**	**POM-$(NH_2)_2$ volume (mL)**	**L1 volume (mL)**	**L2 volume (mL)**	**L3 volume (mL)**	**$Zn(NO_3)_2{\cdot}6H_2O$ volume (mL)**	**Molar ratio/ POM:L1:L2:L3:Zn**	**Single crystal? (Y/N)**	**Phase**
1	1	0.5	0	0	1	1:1:0:0:1.5	N	-
2	1	1	0	0	1	1:2:0:0:1.5	N	-
3	1	2	0	0	1	1:4:0:0:1.5	Y	POMOF1-1
4	1	0	0	0.5	1	1:0:0:1:1.5	N	-
5	1	0	0	1	1	1:0:0:2:1.5	Y	POMOF3-1
6	1	0	0	2	1	1:0:0:4:1.5	N	-
7	1	0	0.5	0	1	1:0:1:0:1.5	N	-
8	1	0	1	0	1	1:0:2:0:1.5	N	-
9	1	0	2	0	1	1:0:4:0:1.5	N	-

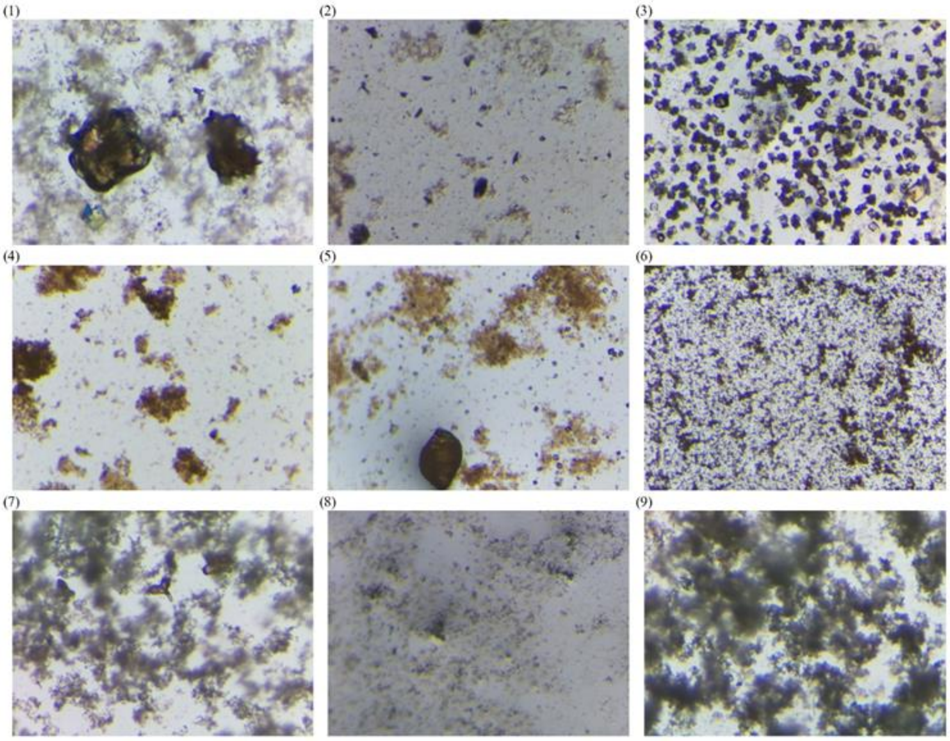

Figure S10: Microscope photos for the 5th synthetic screen.

Table S10: Summary of stock solutions in DMF prepared for the 6th synthetic screen.

Reactant	MW (g/mol)	Stock solution concentration (mg/mL)	Stock solution concentration (mmol/ mL)	Total volume of stock solution made (mL)	Mass of reactant required for stock solution (mg)
POM-$(NH_2)_2$	1882.26	37.64	0.02	50	1882
L1	107.11	4.28	0.04	50	214
L2	107.11	4.28	0.04	50	214
L3	123.11	4.92	0.04	50	246
$Zn(NO_3)_2{\cdot}6H_2O$	297.49	5.95	0.02	100	595

Table S11: Summary of reactant solution volumes used for the 6th synthetic screen.

Reaction number	POM-$(NH_2)_2$ volume (mL)	L1 volume (mL)	L2 volume (mL)	L3 volume (mL)	$Zn(NO_3)_2{\cdot}6H_2O$ volume (mL)	Molar ratio/ POM:L1:L2:L3:Zn	Single crystal? (Y/N)	Phase
1	1	1	0	0	1.5	1:2:0:0:1.5	Y	POMOF1-1
2	1	1.5	0	0	1.5	1:3:0:0:1.5	Y	POMOF1-1
3	1	2	0	0	1.5	1:4:0:0:1.5	Y	POMOF1-1
4	1	3	0	0	1.5	1:6:0:0:1.5	Y	POMOF1-1
5	1	0	1	0	1.5	1:0:2:0:1.5	N	-
6	1	0	1.5	0	1.5	1:0:3:0:1.5	N	-
7	1	0	2	0	1.5	1:0:4:0:1.5	N	-
8	1	0	3	0	1.5	1:0:6:0:1.5	N	-
9	1	0	0	1	1.5	1:0:0:2:1.5	Y	POMOF3-1
10	1	0	0	1.5	1.5	1:0:0:3:1.5	Y	POMOF3-1
11	1	0	0	2	1.5	1:0:0:4:1.5	Y	POMOF3-1
12	1	0	0	3	1.5	1:0:0:6:1.5	Y	POMOF3-1, POMOF3-2
13	1	0.5	0.5	0	1.5	1:1:1:0:1.5	Y	POM-Zn
14	1	0.5	1	0	1.5	1:1:2:0:1.5	N	-
15	1	0.5	1.5	0	1.5	1:1:3:0:1.5	N	-
16	1	1	0.5	0	1.5	1:2:1:0:1.5	Y	POMOF3-1
17	1	0.5	0	0.5	1.5	1:1:0:1:1.5	Y	POMOF3-1
18	1	0.5	0	1	1.5	1:1:0:2:1.5	Y	POMOF3-1
19	1	0.5	0	1.5	1.5	1:1:0:3:1.5	Y	POMOF3-1
20	1	1	0	0.5	1.5	1:2:0:1:1.5	Y	POMOF3-1
21	1	0	0.5	0.5	1.5	1:0:1:1:1.5	N	-
22	1	0	0.5	1	1.5	1:0:1:2:1.5	Y	POMOF3-1
23	1	0	1	0.5	1.5	1:0:2:1:1.5	N	-
24	1	0	1.5	0.5	1.5	1:0:3:1:1.5	N	-

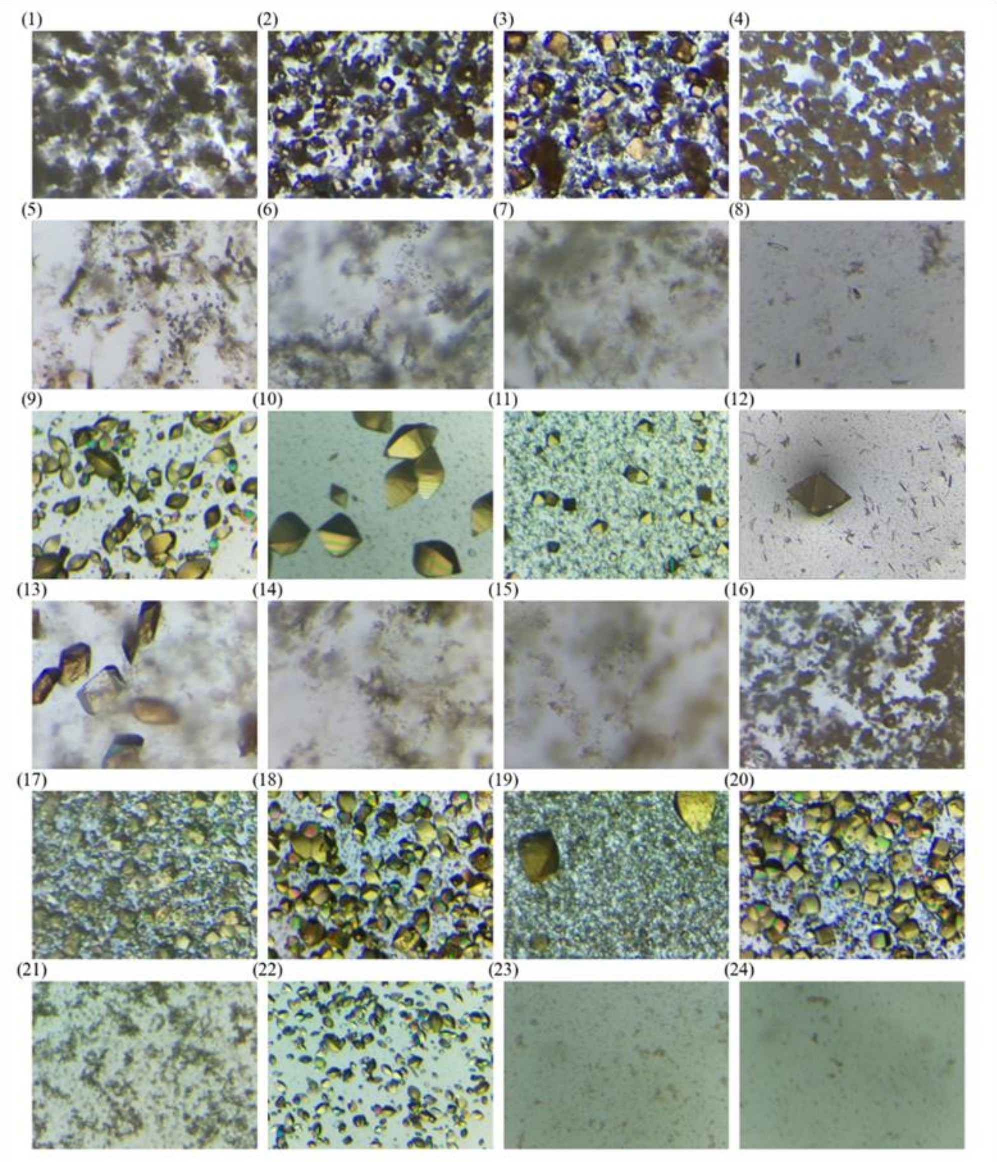

Figure S11: Microscope photos for the 6th synthetic screen.

2.2 Generate initial experiment design randomly

All the added reactant solution volumes should be from 0.1 to 2.0 mL.

Table S12: Summary of stock solutions in DMF prepared for the randomly synthesis.

Reactant	MW (g/mol)	Stock solution concentration (mg/mL)	Stock solution concentration (mmol/ mL)	Total volume of stock solution made (mL)	Mass of reactant required for stock solution (mg)
POM-$(NH_2)_2$	1882.26	56.47	0.03	20	1129.4
L1	107.11	12.85	0.12	25	321.25
L2	107.11	12.85	0.12	25	321.25
L3	123.11	14.77	0.12	25	369.25
$Zn(NO_3)_2{\cdot}6H_2O$	297.49	13.39	0.045	25	334.75

Table S13: Summary of reactant solution volumes used for the randomly synthesis.

Reaction number	POM-$(NH_2)_2$ volume (mL)	L1 volume (mL)	L2 volume (mL)	L3 volume (mL)	DMF volume (mL)	$Zn(NO_3)_2{\cdot}6H_2O$ volume (mL)	Molar ratio/ POM:L1:L2:L3:Zn	Single crystal? (Y/N)	Phase
1	0.81	1.91	1.49	1.24	0.4	0.4	1:9.43:7.36:6.12:0.74	N	-
2	0.21	1.75	1.24	1.45	0.14	1.94	1:33.33:23.62:27.62:13.86	N	-
3	1.68	0.5	0.45	0.45	0.68	1.1	1:1.19:1.07:1.07:0.98	Y	POMOF3-1
4	0.92	0.65	1.26	0.37	0.66	0.8	1:2.83:5.48:1.61:1.3	Y	POMOF3-1
5	0.97	1.59	0.48	1.08	1.23	0.19	1:6.56:1.98:4.45:0.29	N	-
6	1.25	0.42	0.22	1.9	1.93	1.64	1:1.34:0.7:6.08:1.97	Y	POMOF3-1
7	0.68	0.29	1.4	0.94	0.33	1.04	1:1.71:8.24:5.53:2.29	Y	POMOF3-1
8	0.17	1.83	0.59	1.36	0.69	1.09	1:43.06:13.88:32:9.62	N	-
9	1.14	0.45	1.94	1.57	1.89	1.8	1:1.58:6.81:5.51:2.37	Y	POMOF3-1
10	1.24	1.85	0.27	0.47	0.19	0.72	1:5.97:0.87:1.52:0.87	Y	POMOF1+3
11	0.84	0.62	1.67	0.78	0.63	1.13	1:2.95:7.95:3.71:2.02	Y	POMOF3-1
12	0.37	1.62	0.24	1.98	1.57	0.48	1:17.51:2.59:21.41:1.95	N	-
13	0.11	1.65	1.44	1.49	1.57	0.24	1:60:52.36:54.18:3.27	N	-
14	0.78	0.32	1.74	1.28	0.73	0.22	1:1.64:8.92:6.56:0.42	N	-
15	0.69	0.72	1.49	1.31	1.79	1	1:4.17:8.64:7.59:2.17	Y	POMOF3-1
16	0.33	1.46	1.55	1.17	1.56	1.04	1:17.7:18.79:14.18:4.73	Y	POMOF3-2
17	1.09	0.91	0.15	0.3	0.16	1.31	1:3.34:0.55:1.1:1.8	Y	POMOF1-1, POMOF1+3
18	0.7	1.07	1.82	0.57	0.88	1.54	1:6.11:10.4:3.26:3.3	Y	POMOF3-1
19	0.53	0.25	0.65	0.41	1.87	1.64	1:1.89:4.91:3.09:4.64	Y	POMOF3-1
20	1.3	1.76	1.63	0.45	1.8	1.12	1:5.42:5.02:1.38:1.29	Y	POMOF1-1, POMOF1+3

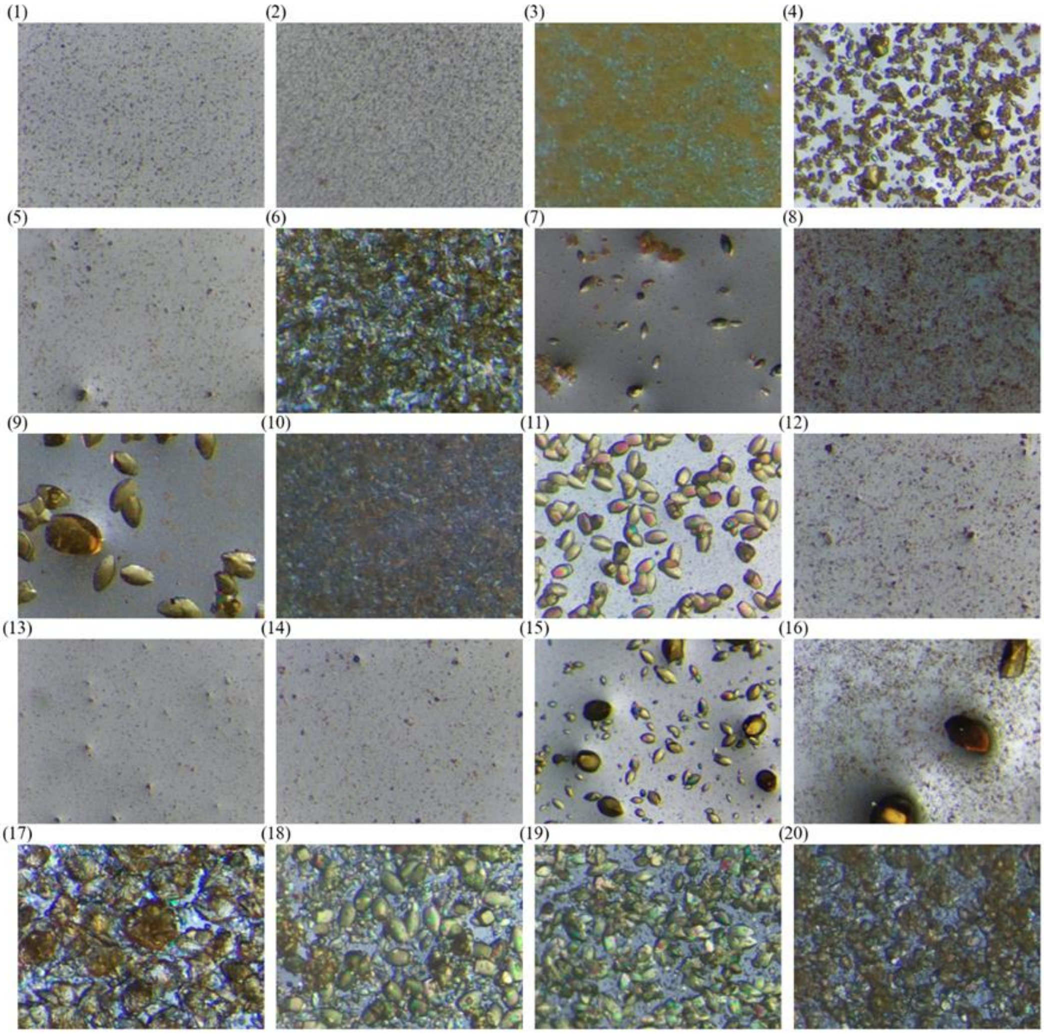

Figure S12: Microscope photos for the randomly synthesis.

2.3 Initial experiment design by chemists

Table S14: Summary of stock solutions in DMF prepared for the initial experiment.

Reactant	MW (g/mol)	Stock solution concentration (mg/mL)	Stock solution concentration (mmol/ mL)	Total volume of stock solution made (mL)	Mass of reactant required for stock solution (mg)
POM-$(NH_2)_2$	1882.26	56.47	0.03	15	847.05
L1	107.11	12.85	0.12	15	192.75
L2	107.11	12.85	0.12	15	192.75
L3	123.11	14.77	0.12	15	221.55
$Zn(NO_3)_2 \cdot 6H_2O$	297.49	13.39	0.045	15	200.85

Table S15: Summary of stock solutions in DMF prepared for the initial experiment.

**Reaction number**	**POM-$(NH_2)_2$ volume (mL)**	**L1 volume (mL)**	**L2 volume (mL)**	**L3 volume (mL)**	**DMF volume (mL)**	**$Zn(NO_3)_2\cdot 6H_2O$ volume (mL)**	**Molar ratio/ POM:L1:L2:L3:Zn**	**Single crystal? (Y/N)**	**Phase**
1	1	1	0	0	1	1	1:4:0:0:1.5	Y	POMOF1-1
2	1	1	1	0	1	1	1:4:4:0:1.5	Y	POMOF1-1
3	1	1	0	1	1	1	1:4:0:4:1.5	Y	POMOF3-2
4	1	1	1	1	1	1	1:4:4:4:1.5	Y	POMOF3-2
5	1	0	1	0	1	1	1:0:4:0:1.5	Y	POMOF2-1, POMOF2-3
6	1	0	1	1	1	1	1:0:4:4:1.5	Y	POMOF3-1, POMOF3-2
7	1	0	0	1	1	1	1:0:0:4:1.5	N	-
8	0.5	0.5	2	0	1	1	1:4:16:0:3	N	-
9	0.5	1	0	0.5	1	1	1:8:0:4:3	Y	POMOF1-1, POMOF3-1, POMOF1+3
10	0.5	0	2	0.5	1	1	1:0:16:4:3	N	-

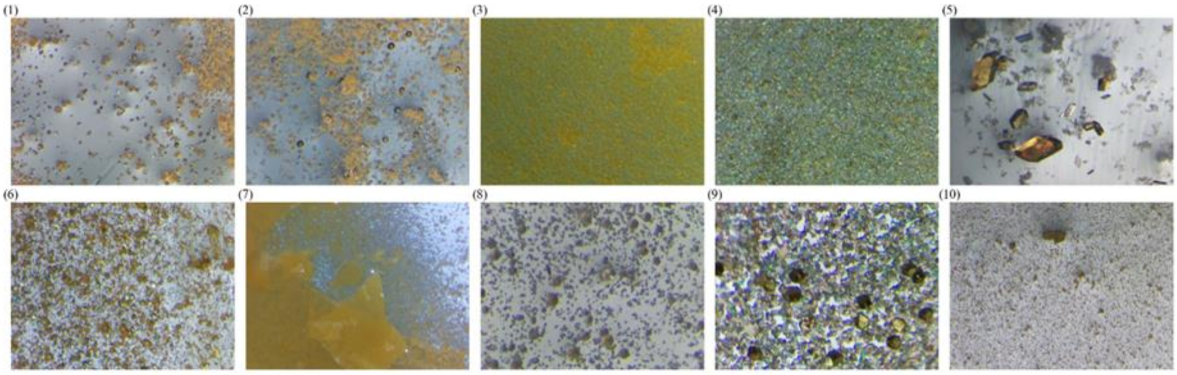

Figure S13: Microscope photos for the products of the initial experiment.

As for the Based on the basic chemistry knowledge, there will be no POMOFs formed if the POM-$(NH_2)_2$ or $Zn(NO_3)_2\cdot 6H_2O$ are not added in the reactions. The results of 65 reactions conditions generated by the random 0 1 matrix with the column for adding POM-$(NH_2)_2$ or $Zn(NO_3)_2\cdot 6H_2O$ as 0, were set up in Class “0” based on knowledge instead of real experiments as a part of initialization dataset.

2.4 Feedback optimization experiment

Table S16: Summary of stock solutions in DMF prepared for the 1st feedback optimization.

Reactant	MW (g/mol)	Stock solution concentration (mg/mL)	Stock solution concentration (mmol/ mL)	Total volume of stock solution made (mL)	Mass of reactant required for stock solution (mg)
POM-$(NH_2)_2$	1882.26	56.47	0.03	8	451.76
L1	107.11	12.85	0.12	20	257
L2	107.11	12.85	0.12	10	128.5
L3	123.11	14.77	0.12	0	0
$Zn(NO_3)_2·6H_2O$	297.49	13.39	0.045	15	200.85

Table S17: Summary of stock solutions in DMF prepared for the 1st feedback optimization.

Reaction number	POM-$(NH_2)_2$ volume (mL)	L1 volume (mL)	L2 volume (mL)	L3 volume (mL)	DMF volume (mL)	$Zn(NO_3)_2$ ·$6H_2O$ volume (mL)	Molar ratio/ POM:L1:L2:L3:Zn	Single crystal? (Y/N)	Phase
1	0.4	2	0.6	0	0.4	0.8	1:20:6:0:3	Y	POMOF1-1
2	0.4	0.4	0.6	0	0.4	1.4	1:4:6:0:5.25	Y	POMOF1-1
3	0.4	0.4	0.6	0	0.4	2	1:4:6:0:7.5	Y	POMOF1-1
4	0.4	0.4	0.6	0	0.6	0.6	1:4:6:0:2.25	Y	POMOF1-1
5	0.4	0.4	0.6	0	0.6	1	1:4:6:0:3.75	Y	POMOF1-1
6	0.4	0.4	0.6	0	0.6	1.6	1:4:6:0:6	Y	POMOF1-1
7	0.4	2	0	0	0.4	0.8	1:20:0:0:3	Y	POMOF1-1
8	0.4	2	0.2	0	0.4	1	1:20:2:0:3.75	Y	POMOF1-1, POMOF1-2
9	0.4	2	0.2	0	0.4	1.6	1:20:2:0:6	Y	POMOF1-1
10	0.4	1.6	0.4	0	0.6	0.8	1:16:4:0:3	Y	POMOF1-1

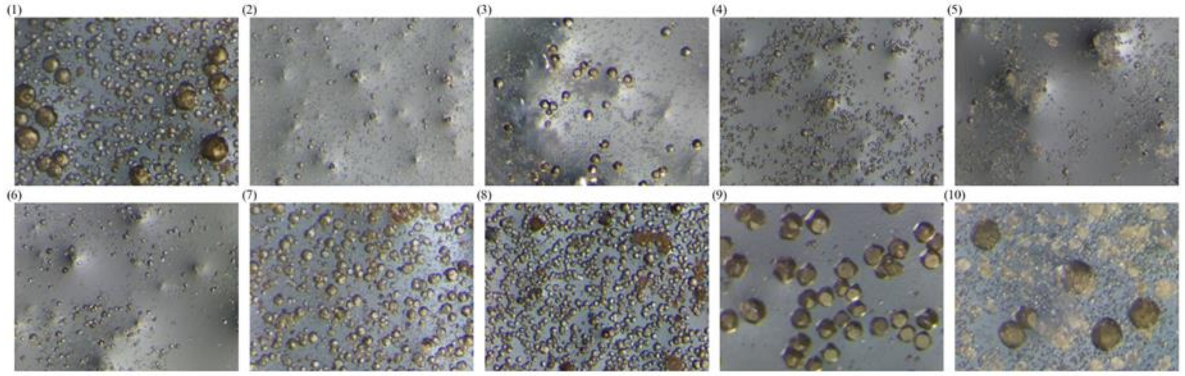

Figure S14: Microscope photos for the products from 1st feedback optimization.

Table S18: Summary of stock solutions in DMF prepared for the 2nd feedback optimization.

Reactant	MW (g/mol)	Stock solution concentration (mg/mL)	Stock solution concentration (mmol/ mL)	Total volume of stock solution made (mL)	Mass of reactant required for stock solution (mg)
POM-$(NH_2)_2$	1882.26	56.47	0.03	15	847.05
L1	107.11	12.85	0.12	0	0
L2	107.11	12.85	0.12	12	154.2
L3	123.11	14.77	0.12	7	103.39
$Zn(NO_3)_2{\cdot}6H_2O$	297.49	13.39	0.045	12	160.68

Table S19: Summary of stock solutions in DMF prepared for the 2nd feedback optimization.

Reaction number	POM-$(NH_2)_2$ volume (mL)	L1 volume (mL)	L2 volume (mL)	L3 volume (mL)	DMF volume (mL)	$Zn(NO_3)_2 \cdot 6H_2O$ volume (mL)	Molar ratio/ POM:L1:L2:L3:Zn	Single crystal? (Y/N)	Phase
1	1.60	0.00	1.60	0.00	0.60	1.00	1:0:4:0:0.94	N	-
2	1.00	0.00	0.60	0.00	1.00	1.00	1:0:2.4:0:1.5	N	-
3	1.00	0.00	0.60	0.00	1.00	0.60	1:0:2.4:0:0.9	N	-
4	1.00	0.00	0.60	0.00	0.60	1.00	1:0:2.4:0:1.5	N	-
5	1.00	0.00	0.60	0.00	0.60	0.60	1:0:2.4:0:0.9	N	-
6	1.60	0.00	1.00	1.60	1.60	1.00	1:0:2.5:4:0.94	Y	POMOF3-1
7	1.00	0.00	0.60	0.00	0.00	1.00	1:0:2.4:0:1.5	N	-
8	1.00	0.00	0.60	0.00	0.00	0.60	1:0:2.4:0:0.9	N	-
9	1.60	0.00	1.00	1.60	1.60	0.60	1:0:2.5:4:0.56	N	-
10	1.00	0.00	0.00	1.60	1.60	1.00	1:0:0:6.4:1.5	Y	POMOF3-1

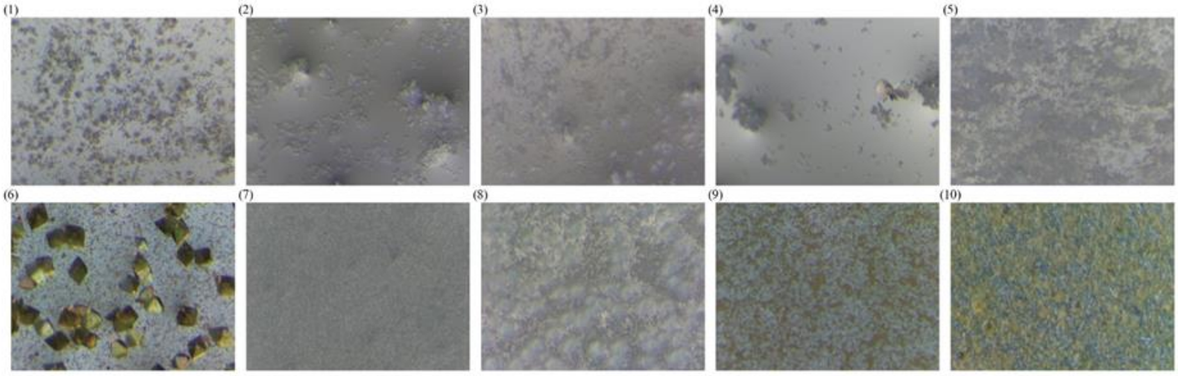

Figure S15: Microscope photos for the products from 2nd feedback optimization.

Table S20: Summary of stock solutions in DMF prepared for the 3rd feedback optimization.

**Reactant**	**MW (g/mol)**	**Stock solution concentration (mg/mL)**	**Stock solution concentration (mmol/ mL)**	**Total volume of stock solution made (mL)**	**Mass of reactant required for stock solution (mg)**
POM-$(NH_2)_2$	1882.26	56.47	0.03	17	959.99
L1	107.11	12.85	0.12	18	231.3
L2	107.11	12.85	0.12	0	0
L3	123.11	14.77	0.12	12	177.24
$Zn(NO_3)_2·6H_2O$	297.49	13.39	0.045	10	133.9

Table S21: Summary of stock solutions in DMF prepared for the 3rd feedback optimization.

**Reaction number**	**POM-$(NH_2)_2$ volume (mL)**	**L1 volume (mL)**	**L2 volume (mL)**	**L3 volume (mL)**	**DMF volume (mL)**	**$Zn(NO_3)_2$ ·$6H_2O$ volume (mL)**	**Molar ratio/ POM:L1:L2:L3:Zn**	**Single crystal? (Y/N)**	**Phase**
1	0.8	1.6	0	0.8	1	0.6	1:8:0:4:1.13	N	-
2	1.2	1.8	0	0.8	2	0.6	1:6:0:2.67:0.75	Y	POMOF1+3
3	1.6	1.4	0	1	1.8	0.6	1:3.5:0:2.5:0.56	Y	POMOF1+3
4	0.8	1.4	0	1	1.6	0.6	1:7:0:5:1.13	Y	POMOF1+3
5	1.4	1.6	0	0.8	1.2	0.6	1:4.57:0:2.29:0.64	Y	POMOF1+3
6	1.6	1.4	0	1	1.2	0.6	1:3.5:0:2.5:0.56	Y	POMOF1+3
7	1.4	1.8	0	0.8	0.8	0.6	1:5.14:0:2.29:0.64	Y	POMOF1+3
8	1.4	1.6	0	1	0.8	0.6	1:4.57:0:2.86:0.64	Y	POMOF1+3
9	1.8	1.4	0	1	0.8	0.6	1:3.11:0:2.22:0.5	Y	POMOF1+3
10	2	1.4	0	0.8	1	0.6	1:2.8:0:1.6:0.45	Y	POMOF1+3

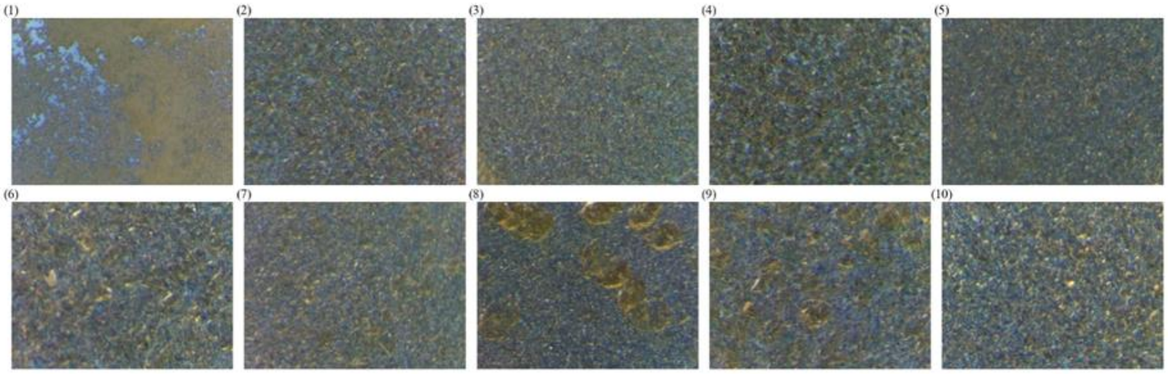

Figure S16: Microscope photos for the products from 3rd feedback optimization.

Table S22: Summary of stock solutions in DMF prepared for the 4th feedback optimization.

Reactant	MW (g/mol)	Stock solution concentration (mg/mL)	Stock solution concentration (mmol/ mL)	Total volume of stock solution made (mL)	Mass of reactant required for stock solution (mg)
POM-$(NH_2)_2$	1882.26	56.47	0.03	9	508.23
L1	107.11	12.85	0.12	10	128.5
L2	107.11	12.85	0.12	16	205.6
L3	123.11	14.77	0.12	0	0
$Zn(NO_3)_2·6H_2O$	297.49	13.39	0.045	15	200.85

Table S23: Summary of stock solutions in DMF prepared for the 4th feedback optimization.

Reaction number	POM-$(NH_2)_2$ volume (mL)	L1 volume (mL)	L2 volume (mL)	L3 volume (mL)	DMF volume (mL)	$Zn(NO_3)_2$ $·6H_2O$ volume (mL)	Molar ratio/ POM:L1:L2:L3:Zn	Single crystal? (Y/N)	Phase
1	0.60	1.00	0.60	0.00	0.00	0.60	1:6.67:4:0:1.5	Y	POMOF1-1
2	0.60	0.60	1.00	0.00	0.00	0.60	1:4:6.67:0:1.5	Y	POMOF1-1
3	0.60	0.60	1.00	0.00	0.00	1.00	1:4:6.67:0:2.5	Y	POMOF1-1
4	0.60	0.60	1.00	0.00	0.00	1.60	1:4:6.67:0:4	Y	POMOF1-1
5	0.60	1.00	1.60	0.00	0.00	1.60	1:6.67:10.67:0:4	Y	POMOF1-1
6	0.60	1.00	1.60	0.00	0.00	1.00	1:6.67:10.67:0:2.5	Y	POMOF1-1
7	0.60	1.00	1.60	0.00	0.00	0.60	1:6.67:10.67:0:1.5	Y	POMOF1-1
8	0.60	0.60	1.60	0.00	0.00	0.60	1:4:10.67:0:1.5	N	-
9	0.60	0.60	1.60	0.00	0.00	1.00	1:4:10.67:0:2.5	N	-
10	0.60	0.60	1.60	0.00	0.00	1.60	1:4:10.67:0:4	Y	POMOF1-1

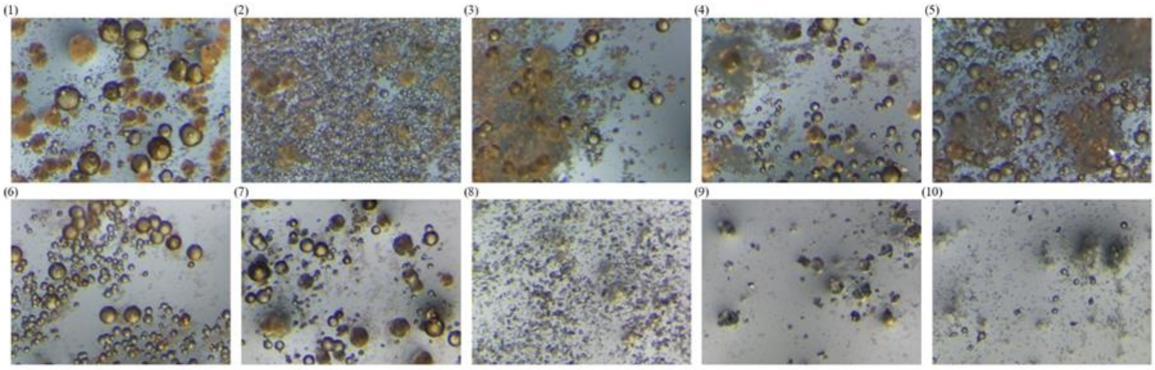

Figure S17: Microscope photos for the products from 4th feedback optimization.

Table S24: Summary of stock solutions in DMF prepared for the 5th feedback optimization.

Reactant	MW (g/mol)	Stock solution concentration (mg/mL)	Stock solution concentration (mmol/ mL)	Total volume of stock solution made (mL)	Mass of reactant required for stock solution (mg)
POM-$(NH_2)_2$	1882.26	56.47	0.03	9	508.23
L1	107.11	12.85	0.12	0	0
L2	107.11	12.85	0.12	15	192.75
L3	123.11	14.77	0.12	15	221.55
$Zn(NO_3)_2 \cdot 6H_2O$	297.49	13.39	0.045	12	160.68

Table S25: Summary of stock solutions in DMF prepared for the 5th feedback optimization.

Reaction number	POM-$(NH_2)_2$ volume (mL)	L1 volume (mL)	L2 volume (mL)	L3 volume (mL)	DMF volume (mL)	$Zn(NO_3)_2 \cdot 6H_2O$ volume (mL)	Molar ratio/ POM:L1:L2:L3:Zn	Single crystal? (Y/N)	Phase
1	0.4	0	1.2	1.4	1.4	0.6	1:0:12:14:2.25	N	-
2	0.6	0	0.2	1.8	1.4	1	1:0:1.33:12:2.5	N	-
3	0.4	0	1	0.6	1.6	0.8	1:0:10:6:3	Y	POMOF3-1
4	0.4	0	1.4	0.6	2	1	1:0:14:6:3.75	Y	POMOF3-1
5	1	0	0.4	0.8	1.4	1	1:0:1.6:3.2:1.5	Y	POMOF3-1
6	0.6	0	1	1.6	1.6	1	1:0:6.67:10.67:2.5	Y	POMOF3-1
7	0.6	0	1	1.6	1.6	0.6	1:0:6.67:10.67:1.5	N	-
8	1	0	0.4	0.8	1.4	0.8	1:0:1.6:3.2:1.2	Y	POMOF3-1
9	0.6	0	1.2	1.8	1.4	1	1:0:8:12:2.5	N	-
10	0.6	0	1.4	2	1.4	0.8	1:0:9.33:13.33:2	N	-

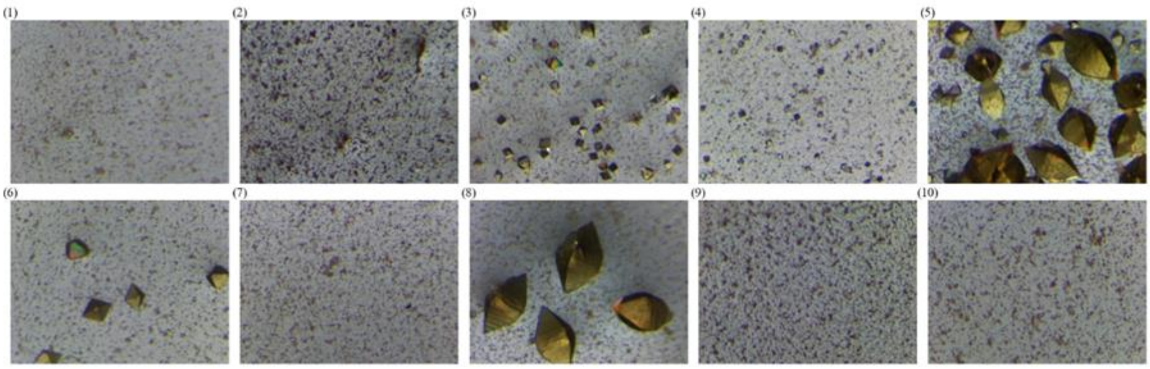

Figure S18: Microscope photos for the products from 5th feedback optimization.

Table S26: Summary of stock solutions in DMF prepared for the 6th feedback optimization.

Reactant	MW (g/mol)	Stock solution concentration (mg/mL)	Stock solution concentration (mmol/ mL)	Total volume of stock solution made (mL)	Mass of reactant required for stock solution (mg)
POM-$(NH_2)_2$	1882.26	56.47	0.03	9	508.23
L1	107.11	12.85	0.12	0	0
L2	107.11	12.85	0.12	15	192.75
L3	123.11	14.77	0.12	15	221.55
$Zn(NO_3)_2\cdot 6H_2O$	297.49	13.39	0.045	12	160.68

Table S27: Summary of stock solutions in DMF prepared for the 6th feedback optimization.

Reaction number	POM-$(NH_2)_2$ volume (mL)	L1 volume (mL)	L2 volume (mL)	L3 volume (mL)	DMF volume (mL)	$Zn(NO_3)_2$ $\cdot 6H_2O$ volume (mL)	Molar ratio/ POM:L1:L2:L3:Zn	Single crystal? (Y/N)	Phase
1	0.4	0	0.8	0	1	0.8	1:0:8:0:3	Y	POMOF2-1
2	0.4	0	1	0	1	0.8	1:0:10:0:3	N	-
3	1.2	0.2	0.2	1.2	1.2	1.2	1:0.67:0.67:4:1.5	Y	POMOF3-1
4	1.2	0.2	0.2	1.2	1.2	1.8	1:0.67:0.67:4:2.25	Y	POMOF3-1
5	1.8	0.2	0.2	1.2	1.2	1.8	1:0.44:0.44:2.67:1.5	Y	POMOF3-1
6	1.8	0.2	0.2	1.2	1.2	1.2	1:0.44:0.44:2.67:1	Y	POMOF3-1
7	0.4	0.2	0.4	0.8	2	0.6	1:2:4:8:2.25	Y	POMOF3-1
8	0.4	0.4	0.8	1.2	1	0.8	1:4:8:12:3	Y	POMOF3-1
9	0.4	0.4	1	1.2	1	0.8	1:4:10:12:3	Y	POMOF3-1
10	0.4	0	1.2	0	0.8	0.8	1:0:12:0:3	Y	POMOF2-1

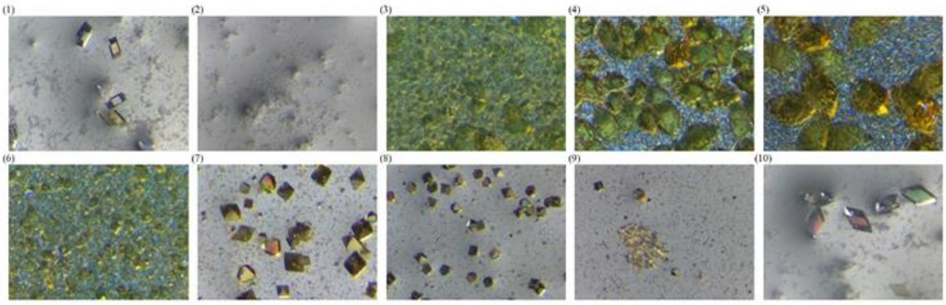

Figure S19: Microscope photos for the products from 6th feedback optimization.

Table S28: Summary of stock solutions in DMF prepared for the 7th feedback optimization.

Reactant	MW (g/mol)	Stock solution concentration (mg/mL)	Stock solution concentration (mmol/ mL)	Total volume of stock solution made (mL)	Mass of reactant required for stock solution (mg)
POM-$(NH_2)_2$	1882.26	56.47	0.03	14	790.58
L1	107.11	12.85	0.12	10	128.5
L2	107.11	12.85	0.12	26	334.1
L3	123.11	14.77	0.12	18	265.86
$Zn(NO_3)_2·6H_2O$	297.49	13.39	0.045	30	401.7

Table S29: Summary of stock solutions in DMF prepared for the 7th feedback optimization.

Reaction number	POM-$(NH_2)_2$ volume (mL)	L1 volume (mL)	L2 volume (mL)	L3 volume (mL)	DMF volume (mL)	$Zn(NO_3)_2$ $·6H_2O$ volume (mL)	Molar ratio/ POM:L1:L2:L3:Zn	Single crystal? (Y/N)	Phase
1	0.6	0	1.2	0.4	0	1	1:0:8:2.67:2.5	N	-
2	0.4	0	1.8	1.4	0	2	1:0:18:14:7.5	N	-
3	0.4	0	1.8	1.4	0	1.4	1:0:18:14:5.25	N	-
4	0.4	0	2	1.4	0.2	0.8	1:0:20:14:3	N	-
5	0.4	0	1.6	0.8	0.2	1.6	1:0:16:8:6	N	-
6	0.4	0	1.6	0.8	0.2	1	1:0:16:8:3.75	N	-
7	0.4	0	2	0.8	0.2	0.8	1:0:20:8:3	N	-
8	0.4	0	1.8	0.4	0	1.4	1:0:18:4:5.25	N	-
9	0.4	0	2	0.4	0.2	0.8	1:0:20:4:3	N	-
10	0.4	0	1.4	2	0.2	2	1:0:14:20:7.5	N	-

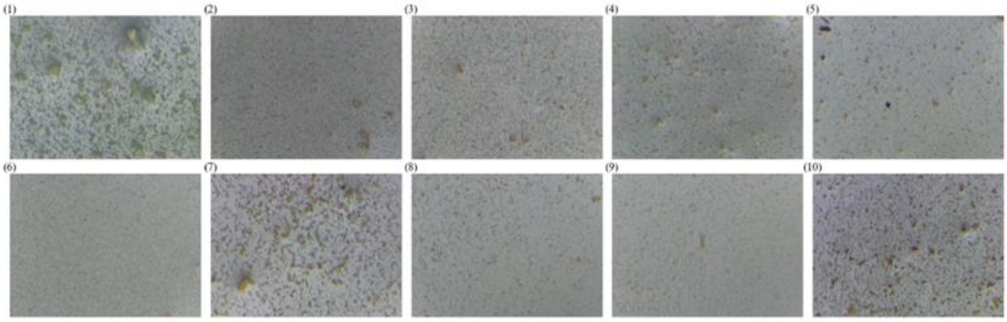

Figure S20: Microscope photos for the products from 7th feedback optimization.

Table S30: Summary of stock solutions in DMF prepared for the 8th feedback optimization.

Reactant	MW (g/mol)	Stock solution concentration (mg/mL)	Stock solution concentration (mmol/ mL)	Total volume of stock solution made (mL)	Mass of reactant required for stock solution (mg)
POM-$(NH_2)_2$	1882.26	56.47	0.03	24	1355.28
L1	107.11	12.85	0.12	12	154.2
L2	107.11	12.85	0.12	25	321.25
L3	123.11	14.77	0.12	21	310.17
$Zn(NO_3)_2{\cdot}6H_2O$	297.49	13.39	0.045	33	441.87

Table S31: Summary of stock solutions in DMF prepared for the 8th feedback optimization.

Reaction number	POM-$(NH_2)_2$ volume (mL)	L1 volume (mL)	L2 volume (mL)	L3 volume (mL)	DMF volume (mL)	$Zn(NO_3)_2$ $\cdot 6H_2O$ volume (mL)	Molar ratio/ POM:L1:L2:L3:Zn	Single crystal? (Y/N)	Phase
1	1.6	0.2	0.2	0.6	0	2	1:0.5:0.5:1.5:1.88	Y	POMOF3-1
2	1.8	0.2	0.2	0.6	0	1.4	1:0.44:0.44:1.33:1.17	Y	POMOF3-1
3	1	0.2	0.2	0.6	0	2	1:0.8:0.8:2.4:3	Y	POMOF3-1
4	1.2	0.2	0.2	0.6	0	1.4	1:0.67:0.67:2:1.75	Y	POMOF3-1
5	1	0	1.6	1.6	1.6	1	1:0:6.4:6.4:1.5	Y	POMOF3-1
6	1	0	1.6	1.6	1.6	1.6	1:0:6.4:6.4:2.4	Y	POMOF3-1
7	0.8	0	1.6	1.4	1.8	1.4	1:0:8:7:2.63	Y	POMOF3-1
8	0.8	0	2	2	2	0.8	1:0:10:10:1.5	N	-
9	0.8	0	1.6	2	2	1.4	1:0:8:10:2.63	Y	POMOF3-1
10	1	0	1.8	1.6	1.6	0.8	1:0:7.2:6.4:1.2	N	-

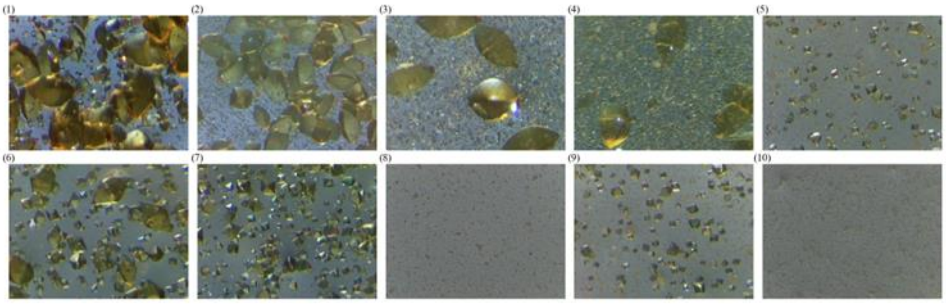

Figure S21: Microscope photos for the products from 8th feedback optimization.

Table S32: Summary of stock solutions in DMF prepared for the 9th feedback optimization.

Reactant	MW (g/mol)	Stock solution concentration (mg/mL)	Stock solution concentration (mmol/ mL)	Total volume of stock solution made (mL)	Mass of reactant required for stock solution (mg)
POM-$(NH_2)_2$	1882.26	56.47	0.03	21	1185.87
L1	107.11	12.85	0.12	8	102.8
L2	107.11	12.85	0.12	20	257
L3	123.11	14.77	0.12	8	118.16
$Zn(NO_3)_2·6H_2O$	297.49	13.39	0.045	20	267.8

Table S33: Summary of stock solutions in DMF prepared for the 9th feedback optimization.

Reaction number	POM-$(NH_2)_2$ volume (mL)	L1 volume (mL)	L2 volume (mL)	L3 volume (mL)	DMF volume (mL)	$Zn(NO_3)_2$ $·6H_2O$ volume (mL)	Molar ratio/ POM:L1:L2:L3:Zn	Single crystal? (Y/N)	Phase
1	2	0	1.6	0	0.8	0.8	1:0:3.2:0:0.6	Y	POMOF2-2
2	1.4	0	1	0	0.8	0.8	1:0:2.86:0:0.86	N	-
3	0.8	0	1.4	0	0.8	0.8	1:0:7:0:1.5	Y	POMOF2-3
4	0.8	0	0.8	0	0.8	0.8	1:0:4:0:1.5	Y	POMOF2-1
5	2	0	1	0	0.8	0.8	1:0:2:0:0.6	N	-
6	0.8	0	2	0	0.8	0.8	1:0:10:0:1.5	Y	POMOF2-3
7	2	0	0.8	0	0.8	0.8	1:0:1.6:0:0.6	N	-
8	1.4	0	1.6	0	0.8	0.8	1:0:4.57:0:0.86	Y	POMOF2-2
9	1.4	0	0.8	0	0.8	0.8	1:0:2.29:0:0.86	N	-
10	0.4	0	1	0	0.8	0.8	1:0:10:0:3	N	-

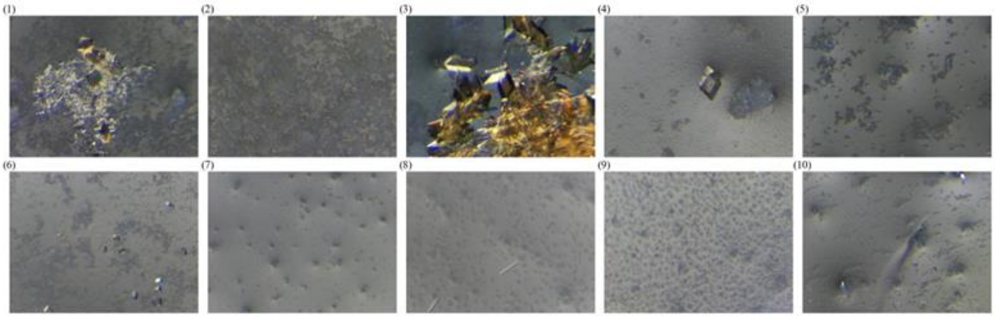

Figure S22: Microscope photos for the products from 9th feedback optimization.

Table S34: Summary of stock solutions in DMF prepared for the 10th feedback optimization.

Reactant	MW (g/mol)	Stock solution concentration (mg/mL)	Stock solution concentration (mmol/ mL)	Total volume of stock solution made (mL)	Mass of reactant required for stock solution (mg)
POM-$(NH_2)_2$	1882.26	56.47	0.03	21	1185.87
L1	107.11	12.85	0.12	11	141.35
L2	107.11	12.85	0.12	20	257
L3	123.11	14.77	0.12	5	73.85
$Zn(NO_3)_2 \cdot 6H_2O$	297.49	13.39	0.045	13	174.07

Table S35: Summary of stock solutions in DMF prepared for the 10th feedback optimization.

Reaction number	POM-$(NH_2)_2$ volume (mL)	L1 volume (mL)	L2 volume (mL)	L3 volume (mL)	DMF volume (mL)	$Zn(NO_3)_2 \cdot 6H_2O$ volume (mL)	Molar ratio/ POM:L1:L2:L3:Zn	Single crystal? (Y/N)	Phase
1	1.8	0	0.8	0	0.8	0.2	1:0:1.78:0:0.17	N	-
2	1.8	0	1.2	0	0.8	0.6	1:0:2.67:0:0.5	N	-
3	1.8	0.2	0.8	0	0.8	0.6	1:0.44:1.78:0:0.5	N	-
4	0.6	0.2	0.8	0	0.8	0.6	1:1.33:5.33:0:1.5	N	-
5	0.6	0.2	0.8	0	0.8	0.2	1:1.33:5.33:0:0.5	N	-
6	1	0.2	1.4	0	0.8	0.4	1:0.8:5.6:0:0.6	N	-
7	0.6	0.2	1.4	0	0.8	0	1:1.33:9.33:0:0	N	-
8	1.2	0	1.2	0	0.8	0.6	1:0:4:0:0.75	Y	POMOF2-2
9	1.6	0.2	1.4	0	0.8	0.4	1:0.5:3.5:0:0.38	N	-
10	1.2	0.2	0.8	0	0.8	0.6	1:0.67:2.67:0:0.75	N	-

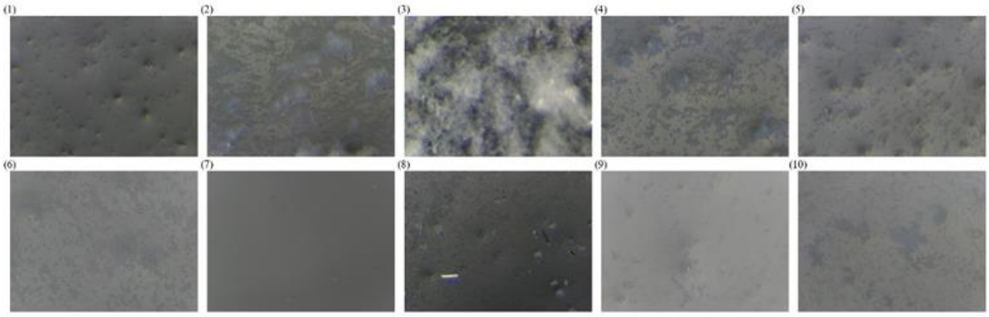

Figure S23: Microscope photos for the products from 10th feedback optimization.

Table S36: Summary of stock solutions in DMF prepared for the 11th feedback optimization.

Reactant	MW (g/mol)	Stock solution concentration (mg/mL)	Stock solution concentration (mmol/ mL)	Total volume of stock solution made (mL)	Mass of reactant required for stock solution (mg)
POM-$(NH_2)_2$	1882.26	56.47	0.03	14	790.58
L1	107.11	12.85	0.12	0	0
L2	107.11	12.85	0.12	15	192.75
L3	123.11	14.77	0.12	0	0
$Zn(NO_3)_2{\cdot}6H_2O$	297.49	13.39	0.045	18	241.02

Table S37: Summary of stock solutions in DMF prepared for the 11th feedback optimization.

Reaction number	POM-$(NH_2)_2$ volume (mL)	L1 volume (mL)	L2 volume (mL)	L3 volume (mL)	DMF volume (mL)	$Zn(NO_3)_2$ $\cdot 6H_2O$ volume (mL)	Molar ratio/ POM:L1:L2:L3:Zn	Single crystal? (Y/N)	Phase
1	1.4	0	1.6	0	0.6	1.6	1:0:4.57:0:1.71	Y	POMOF2-1
2	1.4	0	1.8	0	0.4	1.8	1:0:5.14:0:1.93	Y	POMOF2-1
3	1.4	0	1.8	0	0.4	1.2	1:0:5.14:0:1.29	Y	POMOF2-1<br>POMOF2-2
4	1.4	0	1.6	0	0.6	1	1:0:4.57:0:1.07	Y	POMOF2-2
5	1	0	1	0	1	1.6	1:0:4:0:2.4	Y	POMOF2-1
6	1	0	0.8	0	1.8	2	1:0:3.2:0:3	N	-
7	1	0	1	0	1	1	1:0:4:0:1.5	N	-
8	1	0	0.8	0	1.8	1.4	1:0:3.2:0:2.1	N	-
9	1	0	1	0	1.6	1.6	1:0:4:0:2.4	N	-
10	1	0	0.8	0	1.2	2	1:0:3.2:0:3	Y	POMOF2-2

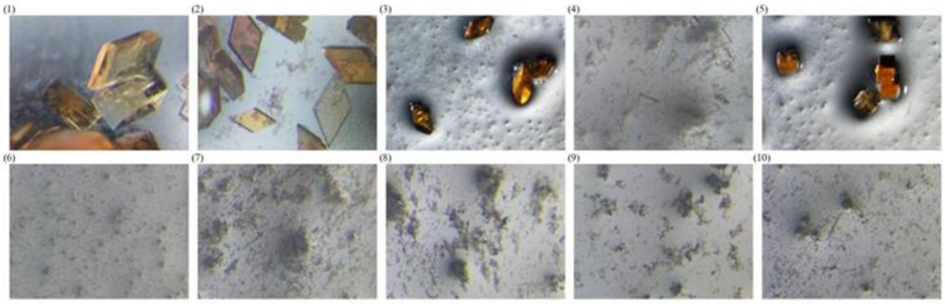

Figure S24: Microscope photos for the products from 11th feedback optimization.

Table S38: Summary of stock solutions in DMF prepared for the 12th feedback optimization.

Reactant	MW (g/mol)	Stock solution concentration (mg/mL)	Stock solution concentration (mmol/ mL)	Total volume of stock solution made (mL)	Mass of reactant required for stock solution (mg)
POM-$(NH_2)_2$	1882.26	56.47	0.03	10	564.7
L1	107.11	12.85	0.12	0	0
L2	107.11	12.85	0.12	17	218.45
L3	123.11	14.77	0.12	0	0
$Zn(NO_3)_2 \cdot 6H_2O$	297.49	13.39	0.045	19	254.41

Table S39: Summary of stock solutions in DMF prepared for the 12th feedback optimization.

Reaction number	POM-$(NH_2)_2$ volume (mL)	L1 volume (mL)	L2 volume (mL)	L3 volume (mL)	DMF volume (mL)	$Zn(NO_3)_2 \cdot 6H_2O$ volume (mL)	Molar ratio/ POM:L1:L2:L3:Zn	Single crystal? (Y/N)	Phase
1	0.6	0	1	0	1	1.6	1:0:6.67:0:4	N	-
2	0.6	0	1.4	0	1.8	1.6	1:0:9.33:0:4	N	-
3	0.6	0	1.6	0	1.6	1.6	1:0:10.67:0:4	N	-
4	0.8	0	1.6	0	1.4	2	1:0:8:0:3.75	N	-
5	1	0	1.6	0	1.6	1.6	1:0:6.4:0:2.4	Y	POMOF2-1
6	1.2	0	1.2	0	2	2	1:0:4:0:2.5	Y	POMOF2-1
7	1	0	2	0	1.4	1.8	1:0:8:0:2.7	N	-
8	0	0	1.2	0	1	1	-	Y	Nicotinic acid
9	0	0	1.6	0	1	1	-	Y	Nicotinic acid
10	1.4	0	1	0	0.8	2	1:0:2.86:0:2.14	Y	POMOF2-1

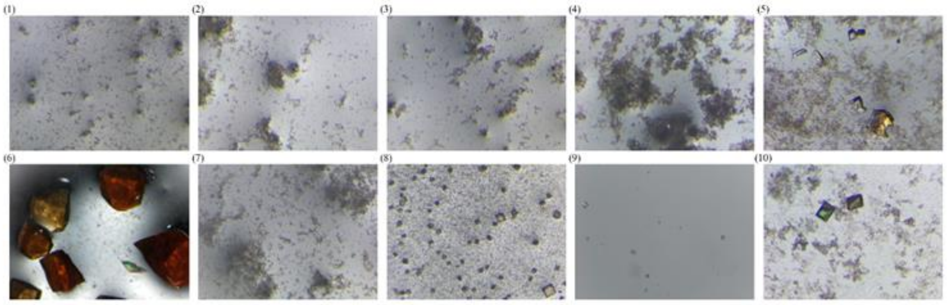

Figure S25: Microscope photos for the products from 12th feedback optimization.

Table S40: Summary of stock solutions in DMF prepared for the 13th feedback optimization.

Reactant	MW (g/mol)	Stock solution concentration (mg/mL)	Stock solution concentration (mmol/ mL)	Total volume of stock solution made (mL)	Mass of reactant required for stock solution (mg)
POM-$(NH_2)_2$	1882.26	56.47	0.03	12	677.64
L1	107.11	12.85	0.12	0	0
L2	107.11	12.85	0.12	15	192.75
L3	123.11	14.77	0.12	0	0
$Zn(NO_3)_2·6H_2O$	297.49	13.39	0.045	13	174.07

Table S41: Summary of stock solutions in DMF prepared for the 13th feedback optimization.

Reaction number	POM-$(NH_2)_2$ volume (mL)	L1 volume (mL)	L2 volume (mL)	L3 volume (mL)	DMF volume (mL)	$Zn(NO_3)_2$ $·6H_2O$ volume (mL)	Molar ratio/ POM:L1:L2:L3:Zn	Single crystal? (Y/N)	Phase
1	1.2	0	0.8	0	2	1.2	1:0:2.67:0:1.5	Y	POM-Zn
2	1.2	0	0.8	0	1.4	1.2	1:0:2.67:0:1.5	Y	POM-Zn
3	1	0	1	0	0.6	1	1:0:4:0:1.5	Y	POMOF2-1
4	1	0	1	0	0	1	1:0:4:0:1.5	Y	POMOF2-3
5	1.2	0	1.4	0	1.2	1	1:0:4.67:0:1.25	Y	POMOF2-2
6	1.2	0	1.8	0	1.6	1	1:0:6:0:1.25	Y	POMOF2-2
7	1.2	0	1.6	0	1.4	1	1:0:5.33:0:1.25	Y	POMOF2-2
8	1.2	0	1.4	0	2	1	1:0:4.67:0:1.25	N	-
9	0.4	0	1.8	0	0.6	1	1:0:18:0:3.75	Y	POMOF2-3
10	0.2	0	1.2	0	0.6	1	1:0:24:0:7.5	Y	POMOF2-3

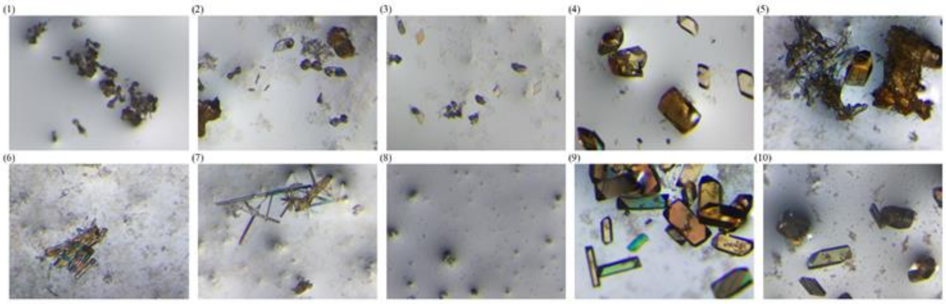

Figure S26: Microscope photos for the products from 13th feedback optimization.

Table S42: Summary of stock solutions in DMF prepared for the 14th feedback optimization.

Reactant	MW (g/mol)	Stock solution concentration (mg/mL)	Stock solution concentration (mmol/ mL)	Total volume of stock solution made (mL)	Mass of reactant required for stock solution (mg)
POM-$(NH_2)_2$	1882.26	56.47	0.03	15	847.05
L1	107.11	12.85	0.12	0	0
L2	107.11	12.85	0.12	17	218.45
L3	123.11	14.77	0.12	0	0
$Zn(NO_3)_2 \cdot 6H_2O$	297.49	13.39	0.045	12	160.68

Table S43: Summary of stock solutions in DMF prepared for the 14th feedback optimization.

Reaction number	POM-$(NH_2)_2$ volume (mL)	L1 volume (mL)	L2 volume (mL)	L3 volume (mL)	DMF volume (mL)	$Zn(NO_3)_2 \cdot 6H_2O$ volume (mL)	Molar ratio/ POM:L1:L2:L3:Zn	Single crystal? (Y/N)	Phase
1	1	0	1.6	0	1.8	0.6	1:0:6.4:0:0.9	Y	POMOF2-2
2	1	0	1.4	0	1.6	0.6	1:0:5.6:0:0.9	Y	POMOF2-2
3	1	0	1.2	0	1.4	0.6	1:0:4.8:0:0.9	Y	POMOF2-2
4	2	0	2	0	1	2	1:0:4:0:1.5	Y	POMOF2-1
5	1.8	0	1.8	0	1	1.2	1:0:4:0:1	Y	POMOF2-2
6	2	0	1.8	0	1	0.8	1:0:3.6:0:0.6	N	-
7	0.8	0	0.8	0	1	1	1:0:4:0:1.88	Y	POM-Zn
8	1	0	1	0	1.4	1.2	1:0:4:0:1.8	Y	POMOF2-1
9	1	0	0.8	0	1.4	0.8	1:0:3.2:0:1.2	Y	POM-Zn
10	0.6	0	1.6	0	0	1	1:0:10.67:0:2.5	Y	POMOF2-3

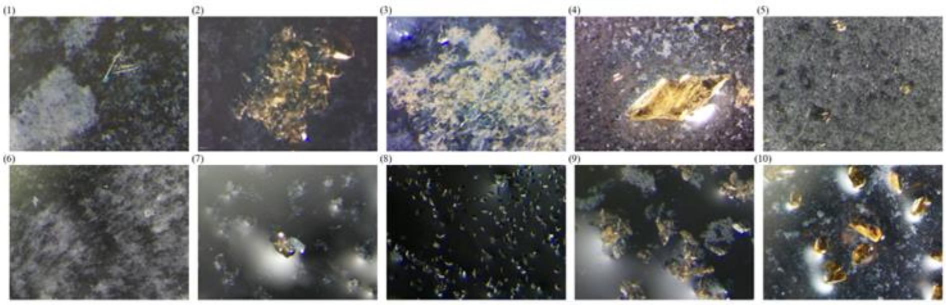

Figure S27: Microscope photos for the products from 14th feedback optimization.

Table S44: Summary of stock solutions in DMF prepared for the 15th feedback optimization.

Reactant	MW (g/mol)	Stock solution concentration (mg/mL)	Stock solution concentration (mmol/ mL)	Total volume of stock solution made (mL)	Mass of reactant required for stock solution (mg)
POM-$(NH_2)_2$	1882.26	56.47	0.03	18	1016.46
L1	107.11	12.85	0.12	10	128.5
L2	107.11	12.85	0.12	17	218.45
L3	123.11	14.77	0.12	9	132.93
$Zn(NO_3)_2{\cdot}6H_2O$	297.49	13.39	0.045	19	254.41

Table S45: Summary of stock solutions in DMF prepared for the 15th feedback optimization.

Reaction number	POM-$(NH_2)_2$ volume (mL)	L1 volume (mL)	L2 volume (mL)	L3 volume (mL)	DMF volume (mL)	$Zn(NO_3)_2{\cdot}6H_2O$ volume (mL)	Molar ratio/ POM:L1:L2:L3:Zn	Single crystal? (Y/N)	Phase
1	1.2	0	1	0	0	1.4	1:0:3.33:0:1.75	Y	POMOF2-1
2	1.2	0	1	0	0	1.8	1:0:3.33:0:2.25	Y	POMOF2-1
3	1	0.4	1.2	0	0	0.8	1:1.6:4.8:0:1.2	N	-
4	1	0.6	1.2	0	0	0.8	1:2.4:4.8:0:1.2	N	-
5	1	1	1.2	0	0	0.8	1:4:4.8:0:1.2	N	-
6	1	1.6	1.2	0	0	0.8	1:6.4:4.8:0:1.2	Y	POMOF1-1
7	1.4	0	1.4	0.8	0.8	1.4	1:0:4:2.29:1.5	Y	POMOF3-3
8	1.4	0	1.4	1.6	0.8	2	1:0:4:4.57:2.14	Y	POMOF3-1<br>POMOF3-3
9	1.4	0	1.4	2	0.8	1.4	1:0:4:5.71:1.5	N	-
10	1.4	0	1.4	0.4	0.8	1.4	1:0:4:1.14:1.5	N	-

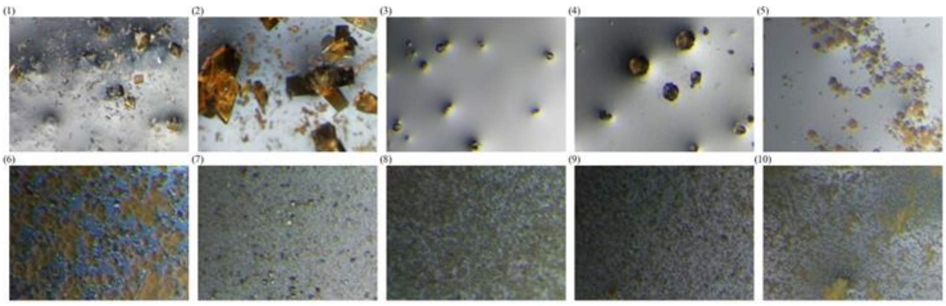

Figure S28: Microscope photos for the products from 15th feedback optimization.

Table S46: Summary of stock solutions in DMF prepared for the 16th feedback optimization.

Reactant	MW (g/mol)	Stock solution concentration (mg/mL)	Stock solution concentration (mmol/ mL)	Total volume of stock solution made (mL)	Mass of reactant required for stock solution (mg)
POM-$(NH_2)_2$	1882.26	56.47	0.03	12	677.64
L1	107.11	12.85	0.12	0	0
L2	107.11	12.85	0.12	15	192.75
L3	123.11	14.77	0.12	0	0
$Zn(NO_3)_2{\cdot}6H_2O$	297.49	13.39	0.045	14	187.46

Table S47: Summary of stock solutions in DMF prepared for the 16th feedback optimization.

Reaction number	POM-$(NH_2)_2$ volume (mL)	L1 volume (mL)	L2 volume (mL)	L3 volume (mL)	DMF volume (mL)	$Zn(NO_3)_2$ $\cdot6H_2O$ volume (mL)	Molar ratio/ POM:L1:L2:L3:Zn	Single crystal? (Y/N)	Phase
1	1.4	0	1.2	0	1	1.4	1:0:3.43:0:1.5	Y	POMOF2-2, POMOF2-1
2	1.8	0	1	0	1	1.2	1:0:2.22:0:1	N	-
3	1	0	1	0	1	0.4	1:0:4:0:0.6	N	-
4	0.8	0	1	0	1	0.4	1:0:5:0:0.75	Y	POMOF2-2
5	0.2	0	1	0	0.2	0.6	1:0:20:0:4.5	Y	POMOF2-3
6	0.2	0	1.8	0	0.8	0.4	1:0:36:0:3	Y	POMOF2-3
7	0.2	0	1.4	0	0.8	0.4	1:0:28:0:3	Y	POMOF2-3
8	1	0	1.2	0	1.2	2	1:0:4.8:0:3	Y	POM-Zn
9	1	0	1	0	1.6	1.6	1:0:4:0:2.4	Y	POM-Zn
10	0.8	0	1	0	1.4	1.6	1:0:5:0:3	N	-
11	1	0	1	0	1	1	1:0:4:0:1.5	Y	POMOF2-1

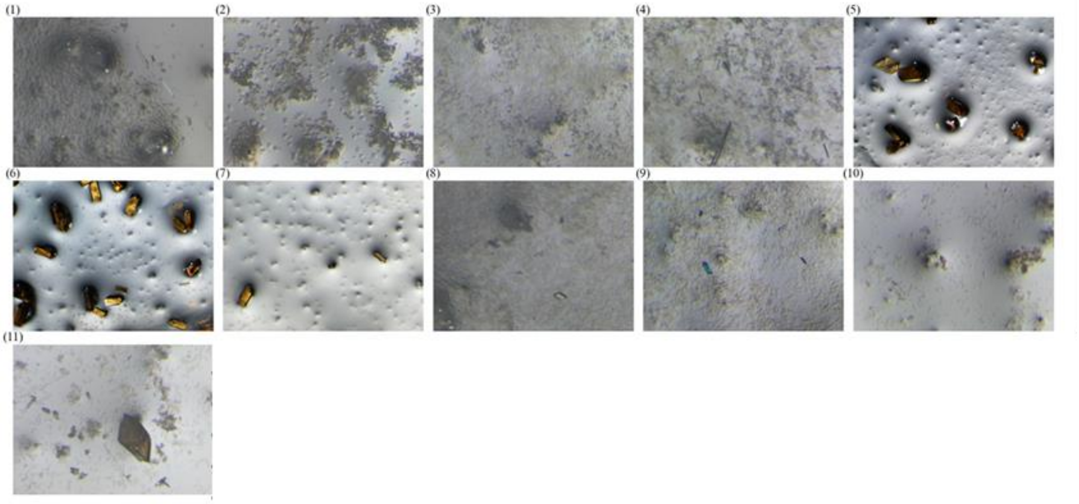

Figure S29: Microscope photos for the products from 16th feedback optimization.

Table S48: Summary of stock solutions in DMF prepared for the 17th feedback optimization.

Reactant	MW (g/mol)	Stock solution concentration (mg/mL)	Stock solution concentration (mmol/ mL)	Total volume of stock solution made (mL)	Mass of reactant required for stock solution (mg)
POM-$(NH_2)_2$	1882.26	56.47	0.03	9	508.23
L1	107.11	12.85	0.12	0	0
L2	107.11	12.85	0.12	15	192.75
L3	123.11	14.77	0.12	0	0
$Zn(NO_3)_2·6H_2O$	297.49	13.39	0.045	12	160.68

Table S49: Summary of stock solutions in DMF prepared for the 17th feedback optimization.

Reaction number	POM-$(NH_2)_2$ volume (mL)	L1 volume (mL)	L2 volume (mL)	L3 volume (mL)	DMF volume (mL)	$Zn(NO_3)_2$ ·$6H_2O$ volume (mL)	Molar ratio/ POM:L1:L2:L3:Zn	Single crystal? (Y/N)	Phase
1	0.8	0	1.8	0	2	1.6	1:0:9:0:3	Y	POM-Zn POMOF2-1
2	0.8	0	1.8	0	1.4	1.6	1:0:9:0:3	Y	POMOF2-1
3	0.2	0	1.6	0	1	0.2	1:0:32:0:1.5	Y	POMOF2-3
4	1	0	0.8	0	0.8	1.2	1:0:3.2:0:1.8	Y	POM-Zn
5	0.8	0	0.8	0	0.8	1.2	1:0:4:0:2.25	Y	POMOF2-2, POMOF2-1
6	0.6	0	1	0	0.6	0.6	1:0:6.67:0:1.5	Y	POMOF2-2
7	0.6	0	1	0	0.6	1	1:0:6.67:0:2.5	Y	POMOF2-3, POMOF2-2
8	0.6	0	1	0	0	0.6	1:0:6.67:0:1.5	Y	POMOF2-3, POMOF2-2
9	0.6	0	1	0	0	1	1:0:6.67:0:2.5	Y	POMOF2-3, POMOF2-2
10	0.6	0	1.6	0	1.4	0.4	1:0:10.67:0:1	N	-

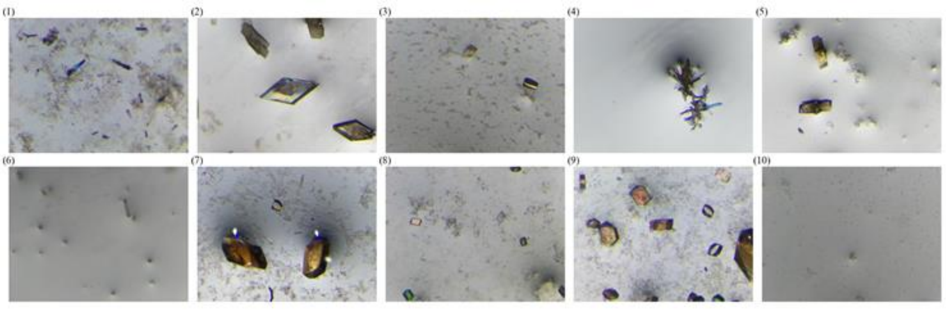

Figure S30: Microscope photos for the products from 17th feedback optimization.

Table S50: Summary of stock solutions in DMF prepared for the 18th feedback optimization.

Reactant	MW (g/mol)	Stock solution concentration (mg/mL)	Stock solution concentration (mmol/ mL)	Total volume of stock solution made (mL)	Mass of reactant required for stock solution (mg)
POM-$(NH_2)_2$	1882.26	56.47	0.03	14	790.58
L1	107.11	12.85	0.12	0	0
L2	107.11	12.85	0.12	14	179.9
L3	123.11	14.77	0.12	0	0
$Zn(NO_3)_2·6H_2O$	297.49	13.39	0.045	13	174.07

Table S51: Summary of stock solutions in DMF prepared for the 18th feedback optimization.

Reaction number	POM-$(NH_2)_2$ volume (mL)	L1 volume (mL)	L2 volume (mL)	L3 volume (mL)	DMF volume (mL)	$Zn(NO_3)_2$ $·6H_2O$ volume (mL)	Molar ratio/ POM:L1:L2:L3:Zn	Single crystal? (Y/N)	Phase
1	0.6	0	1.2	0	1.2	0.6	1:0:8:0:1.5	Y	POMOF2-3, POMOF2-2
2	0.6	0	1.2	0	1.2	0.4	1:0:8:0:1	Y	POMOF2-3, POMOF2-2
3	1.4	0	1	0	1.2	0.6	1:0:2.86:0:0.64	Y	POMOF2-2
4	1.4	0	1	0	1.2	0.4	1:0:2.86:0:0.43	Y	POMOF2-2
5	1	0	1.4	0	1.2	1.6	1:0:5.6:0:2.4	Y	POMOF2-1
6	1	0	0.8	0	1.2	0.4	1:0:3.2:0:0.6	Y	POMOF2-2
7	1	0	0.8	0	1.2	0.8	1:0:3.2:0:1.2	Y	POMOF2-2
8	1	0	1.8	0	1.4	2	1:0:7.2:0:3	Y	POMOF2-1
9	0.4	0	1.8	0	1	0.2	1:0:18:0:0.75	Y	POMOF2-2
10	1.4	0	0.8	0	2	1.6	1:0:2.29:0:1.71	Y	POM-Zn

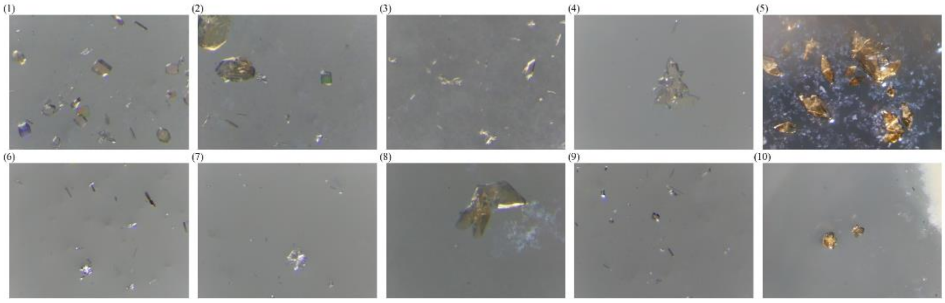

Figure S31: Microscope photos for the products from 18th feedback optimization.

Table S52: Summary of stock solutions in DMF prepared for experiments used for evaluating model accuracy.

**Reactant**	**MW (g/mol)**	**Stock solution concentration (mg/mL)**	**Stock solution concentration (mmol/ mL)**	**Total volume of stock solution made (mL)**	**Mass of reactant required for stock solution (mg)**
POM-$(NH_2)_2$	1882.26	56.47	0.03	27	1524.69
L1	107.11	12.85	0.12	14	179.9
L2	107.11	12.85	0.12	14	179.9
L3	123.11	14.77	0.12	14	206.78
$Zn(NO_3)_2{\cdot}6H_2O$	297.49	13.39	0.045	29	388.31

Table S53: Summary of stock solutions in DMF prepared for experiments used for evaluating model accuracy.

Reaction number	POM-$(NH_2)_2$ volume (mL)	L1 volume (mL)	L2 volume (mL)	L3 volume (mL)	DMF volume (mL)	$Zn(NO_3)_2 \cdot 6H_2O$ volume (mL)	Molar ratio/ POM:L1:L2:L3:Zn	Single crystal? (Y/N)	Phase
1	0.6	1.2	0.3	0	1	1	1:8:2:0:2.5	Y	POMOF1-1, POMOF1-2
2	0.9	0.9	0.6	0	1	1	1:4:2.67:0:1.67	Y	POMOF1-1
3	1.2	0.6	0.9	0	1.2	1.2	1:2:3:0:1.5	N	-
4	1.5	0.3	1.2	0	1.2	1.5	1:0.8:3.2:0:1.5	N	-
5	1.8	0	1.8	0	1.2	1.8	1:0:4:0:1.5	Y	POMOF2-1, POMOF2-2
6	0.6	1.2	0	0.3	1	1	1:8:0:2:2.5	Y	POMOF1-1, POMOF1+3
7	0.9	0.9	0	0.6	1	1	1:4:0:2.67:1.67	Y	POMOF3-3
8	1.2	0.6	0	0.9	1.2	1.2	1:2:0:3:1.5	Y	POMOF3-3
9	1.5	0.3	0	1.2	1.2	1.5	1:0.8:0:3.2:1.5	Y	POMOF3-3
10	1.8	0	0	1.8	1.2	1.8	1:0:0:4:1.5	Y	POMOF3-3
11	0.6	0	1.2	0.3	1	1	1:0:8:2:2.5	N	-
12	0.9	0	0.9	0.6	1	1	1:0:4:2.67:1.67	Y	POMOF3-1
13	1.2	0	0.6	0.9	1.2	1.2	1:0:2:3:1.5	Y	POMOF3-1
14	1.5	0	0.3	1.2	1.2	1.5	1:0:0.8:3.2:1.5	Y	POMOF3-1
15	1.8	1.8	0	0	1.2	1.8	1:4:0:0:1.5	Y	POMOF1-1
16	0.6	0.6	0.3	0.3	1	1	1:4:2:2:2.5	Y	POMOF3-1, POMOF3-3
17	0.9	0.3	0.9	0.3	1	1	1:1.33:4:1.33:1.67	Y	POMOF3-1
18	1.2	0.9	0.3	1.2	1.2	1.2	1:3:1:4:1.5	Y	POMOF3-3
19	1.5	1.2	0.6	0.9	1.2	1.5	1:3.2:1.6:2.4:1.5	Y	POMOF3-1, POMOF3-3
20	1.8	0.6	1.2	0.6	1.2	1.8	1:1.33:2.67:1.33:1.5	Y	POMOF3-1

Table S54: Comparison of out-of-sample prediction results made by the XGBoost model with the actual reaction outcomes.

**Reaction number**	**Prediction (8th cycle)**	**Prediction results (Final models)**				**Experimental results**			
	**POMOFx**	**POMOFx**	**POMOF1**	**POMOF2**	**POMOF3**	**POMOFx**	**POMOF1**	**POMOF2**	**POMOF3**
1	1	1	1	0	0	1	1	0	0
2	1	1	1	0	0	1	1	0	0
3	1	1	0	0	0	0	0	0	0
4	1	1	0	0	0	0	0	0	0
5	0	0	0	1	0	1	0	1	0
6	1	1	1	0	1	1	1	0	0
7	1	1	1	0	1	1	0	0	1
8	1	1	1	0	1	1	0	0	1
9	1	1	1	0	1	1	0	0	1
10	1	1	0	0	1	1	0	0	1
11	0	1	0	0	1	0	0	0	0
12	0	1	0	0	1	1	0	0	1
13	1	1	0	0	1	1	0	0	1
14	1	1	0	0	1	1	0	0	1
15	1	1	1	0	0	1	1	0	0
16	1	1	1	0	1	1	0	0	1
17	1	1	0	0	1	1	0	0	1
18	1	1	0	0	1	1	0	0	1
19	1	1	0	0	1	1	0	0	1
20	1	1	0	0	1	1	0	0	1

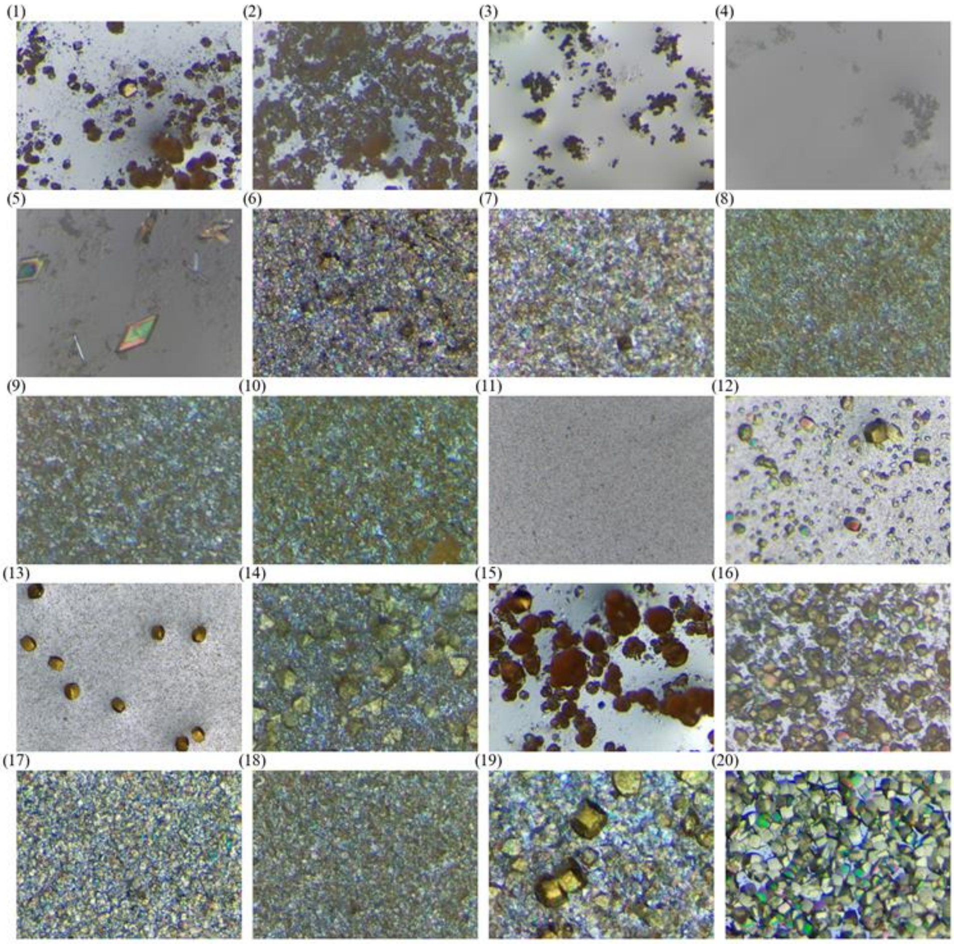

Figure S32: Microscope photos for the products from experiments used for evaluating model accuracy.

2.5 The unique digital signature for the synthesis of POMOFs

In order to describe the synthetic procedure in a unique and standard way, the universal chemical description language (χDL)[3, 4] was implemented to create unique digital signatures for the synthesis of POMOFs. The POM used for the reaction was represented by the standard full name: "δ-Tris-based Mn-Anderson". All other chemicals were represented using their CAS numbers, with corresponding concentrations in the unit of M (molar per litre).

The code for χDL is available at https://croningroup.gitlab.io/chemputer/xdl/standard/index.html and https://gitlab.com/croningroup/chemputer/xdl.git.

```
<Synthesis>
  <Hardware>
    <Component
      id="vial 0"
      type="reactor"
    />
...
  <Reagents>
    <Reagent
      name="10196-18-6 (0.045 M in 68-12-2)"
    />
    <Reagent
      name="delta-tris-based Mn-Anderson (0.03 M in 68-12-2)"
    />
    <Reagent
      name="872-85-5 (0.12 M in 68-12-2)"
    />
    <Reagent
      name="500-22-1 (0.12 M in 68-12-2)"
    />
    <Reagent
      name="1849-54-3 (0.12 M in 68-12-2)"
    />
    <Reagent
      name="68-12-2"
    />
  </Reagents>

  <Procedure>
...

    <Add
      vessel="vial 0"
      reagent="delta-tris-based Mn-Anderson (0.03 M in 68-12-2)“
      volume="0.4 mL"
      speed="5000"
      stir="False"
    />
...
    <Wait
      time="3600"
    />

    <Add
      vessel="vial 0"
      reagent="10196-18-6 (0.045 M in 68-12-2) “
      volume=“1.6 mL"
      speed="5000"
      stir="False"
    />
...
    <HeatChillToTemp
      vessel="vial 0"
      temp="80"
    />
...

    <Wait
      time="172800"
    />

    <HeatChillToTemp
      vessel="vial 0"
      temp=“30"
...
  </Procedure>

</Synthesis>
```


Figure S33: The χDL snippet representing the unique digital signatures of the synthesis POMOFs.

Table S55: Summary of stock solutions in DMF prepared for the synthesis driven by the χDL.

Reactant	MW (g/mol)	Stock solution concentration (mg/mL)	Stock solution concentration (mmol/ mL)	Total volume of stock solution made (mL)	Mass of reactant required for stock solution (mg)
POM-$(NH_2)_2$	1882.26	56.47	0.03	13	734.11
L1	107.11	12.85	0.12	14	179.9
L2	107.11	12.85	0.12	9	115.65
L3	123.11	14.77	0.12	7	103.39
$Zn(NO_3)_2·6H_2O$	297.49	13.39	0.045	15	200.85

Table S56: Summary of stock solutions in DMF prepared for the synthesis driven by the χDL. The amount of the product obtained from the reaction in this table was weighted after the product washed by the DMF and dried in the vacuum at RT for 12 h.

Reaction number	POM-$(NH_2)_2$ volume (mL)	L1 volume (mL)	L2 volume (mL)	L3 volume (mL)	DMF volume (mL)	$Zn(NO_3)_2$ $·6H_2O$ volume (mL)	Molar ratio/ POM:L1: L2:L3:Zn	Product obtained (mg)	Sample standard deviation for yield (mg)	Phase
1	0.4	2	0.2	0	0.4	1.6	1:20:2:0:6	21.0	0.15	POMOF1-1
2	0.4	2	0.2	0	0.4	1.6	1:20:2:0:6	20.7		POMOF1-1
3	0.4	2	0.2	0	0.4	1.6	1:20:2:0:6	20.8		POMOF1-1
4	0.8	0	1.4	0	0.8	0.8	1:0:7:0:1.5	22.5	3.77	POMOF2-3
5	0.8	0	1.4	0	0.8	0.8	1:0:7:0:1.5	19.5		POMOF2-3
6	0.8	0	1.4	0	0.8	0.8	1:0:7:0:1.5	27.0		POMOF2-3
7	1	0	0.4	0.8	1.4	1	1:0:1.6:3.2: 1.5	25.2	0.40	POMOF3-1
8	1	0	0.4	0.8	1.4	1	1:0:1.6:3.2: 1.5	24.9		POMOF3-1
9	1	0	0.4	0.8	1.4	1	1:0:1.6:3.2: 1.5	25.7		POMOF3-1
10	1.4	1.8	0	0.8	0.8	0.6	1:5.14:0:2. 29:0.64	39.7	2.17	POMOF1+3
11	1.4	1.8	0	0.8	0.8	0.6	1:5.14:0:2. 29:0.64	36.0		POMOF1+3
12	1.4	1.8	0	0.8	0.8	0.6	1:5.14:0:2. 29:0.64	39.8		POMOF1+3

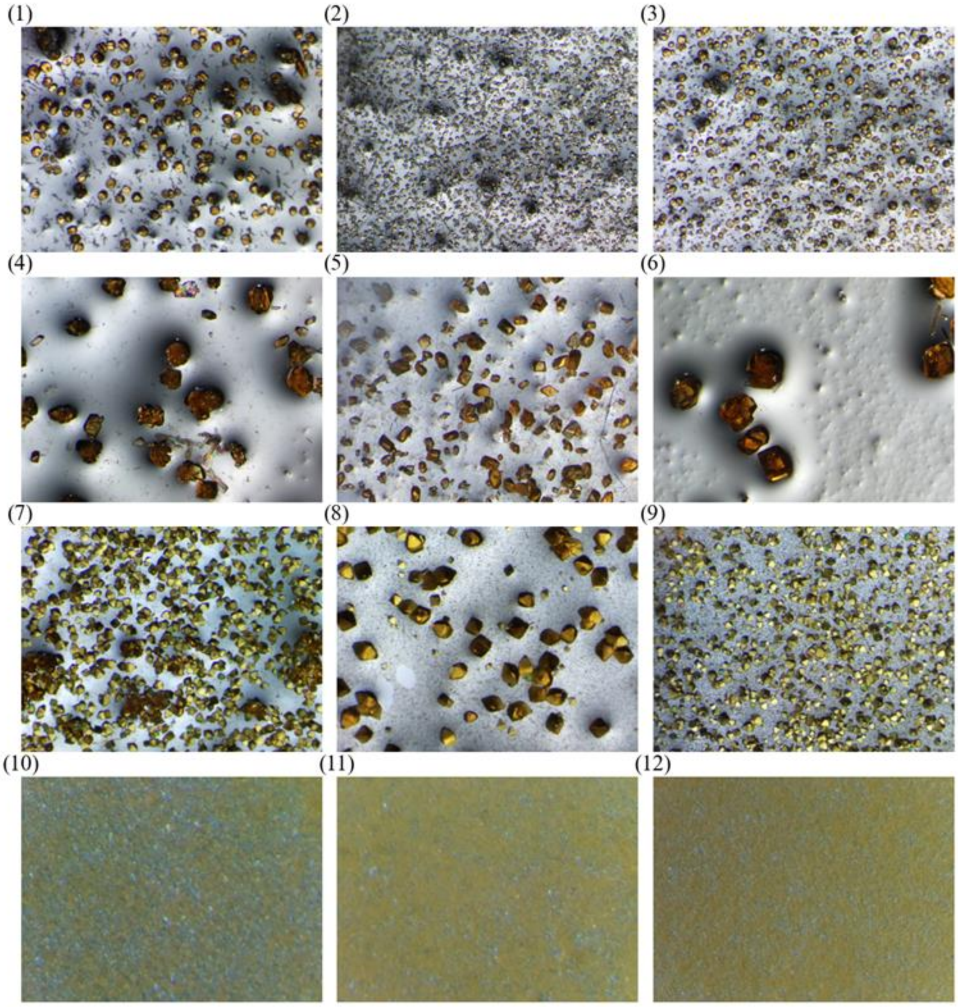

Figure S34: Microscope photos for the products from the synthesis driven by the χDL.

3. XGBoost accuracy

After data re-processing for training each independent model, the F1 of the POMOFx, POMOF1, and POMOF3 models was around 0.8 after 8 cycles of the optimization. (Figure S36, S38 and S39). Despite this, none of the models are effective at predicting the outcome of reaction conditions with L2 adding; only 3, 5, and 5 out of 10 reaction outcomes were correctly predicted by POMOFx, POMOF1, and POMOF3 models respectively (cycle 9, table S33). To further improve the accuracy of the models, the new data set used for the POMOF2 model was also added for training the other models. It should be noted that the result for the same reactions can be classified into “0” or “1” differently for the different POMOF systems. As shown in Figure S36, S38 and S39, the accuracy of the POMOFx, POMOF1 and POMOF3 models did not have a purely linear relationship with the iterative refinement process, in fact, we observed that the accuracy of the models began to decrease in the last few cycles. As the number of positive POMOF2 results in the dataset increased, we observed more positive results in the POMOFx system but less positive results for POMOF1 and POMOF3 systems. An overabundance of either positive or negative results reduces the accuracy of the model. Therefore, the most appropriate dataset was selected based on it having an F1 > 0.8, and the relative highest PD. The final models for POMOFx, POMOF1, POMOF2 and POMOF3 were trained based on the data of 12 cycles, 12 cycles, 18 cycles and 14 cycles respectively.

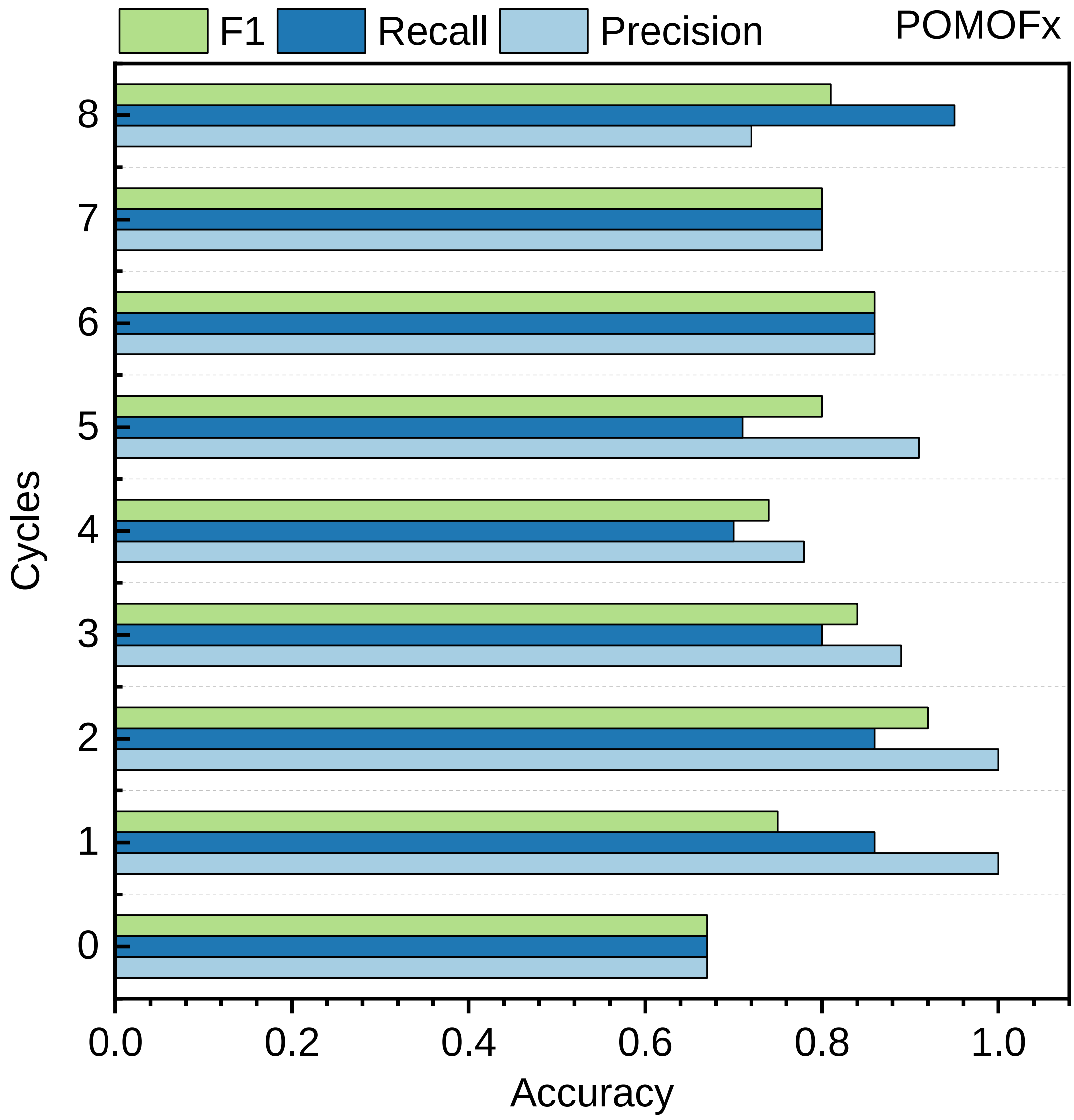

Figure S35: Accuracy of the model for POMOFx (all type of POMOFs) from initialization to the eighth optimization cycle.

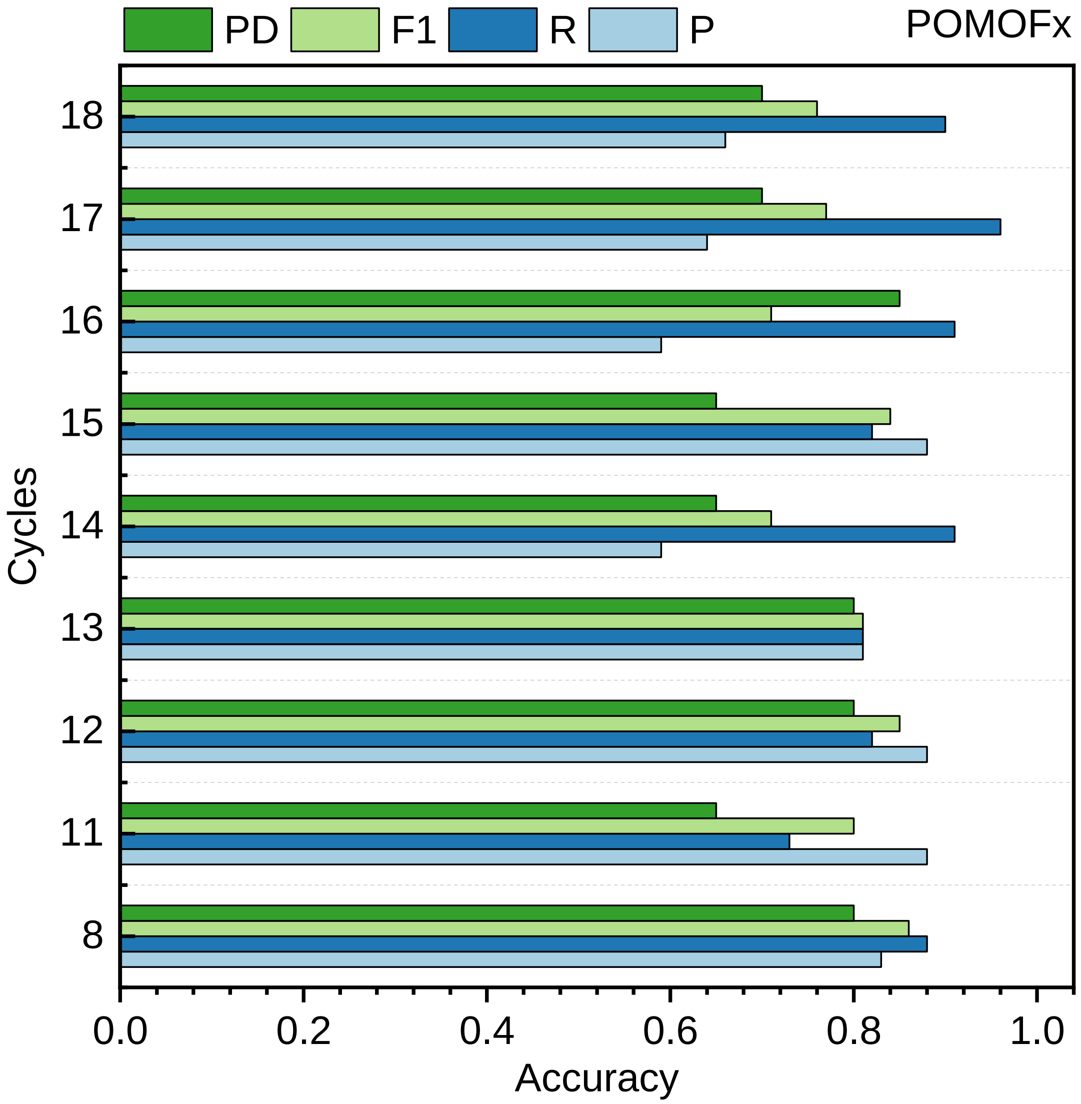

Figure S36: Accuracy of the model for POMOFx (all type of POMOFs) based on the data collected by the optimization of POMOF2 model from 8th to the 18th optimization cycle.

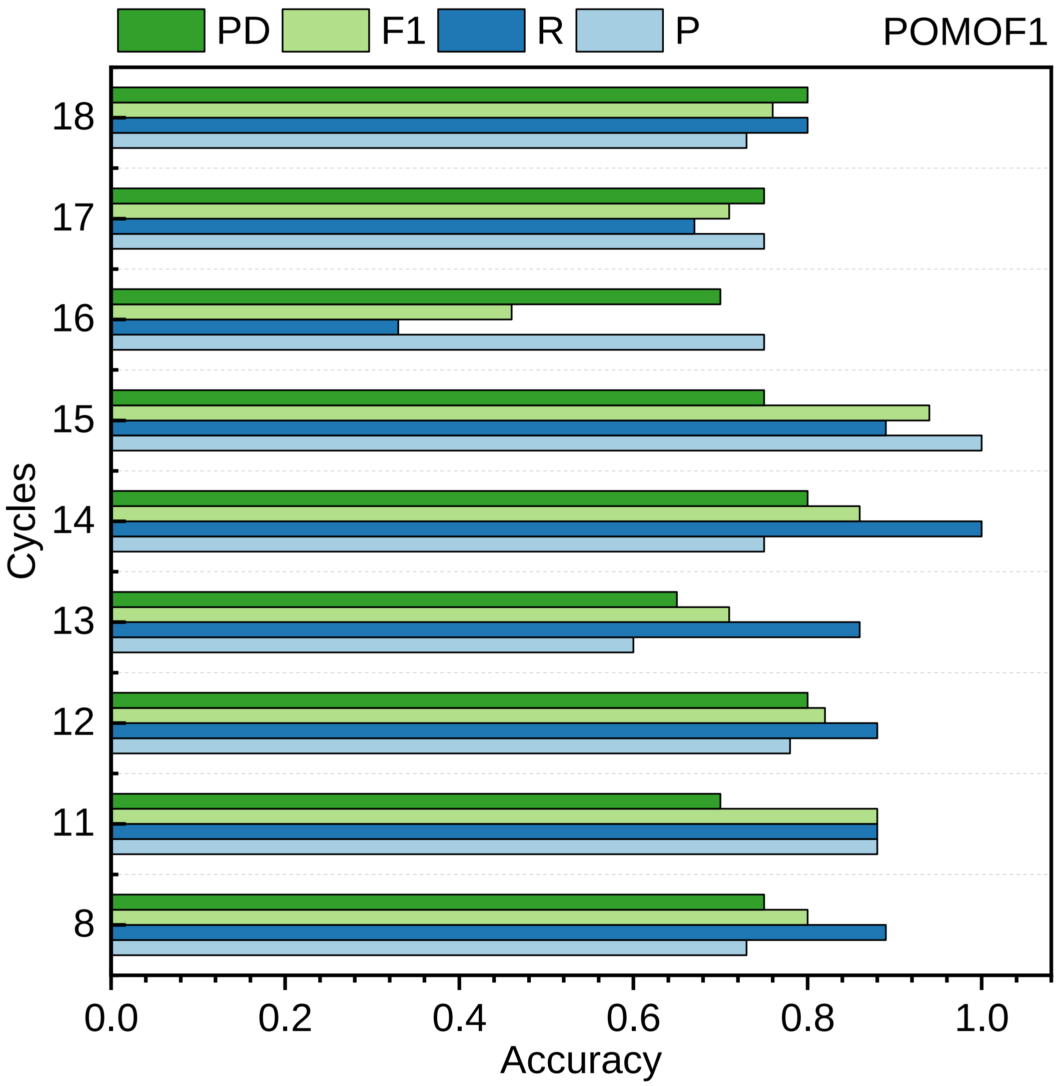

Figure S37: Accuracy of the model for POMOF1 based on the data collected by the optimization of POMOF2 model from 8th to the 18th optimization cycle.

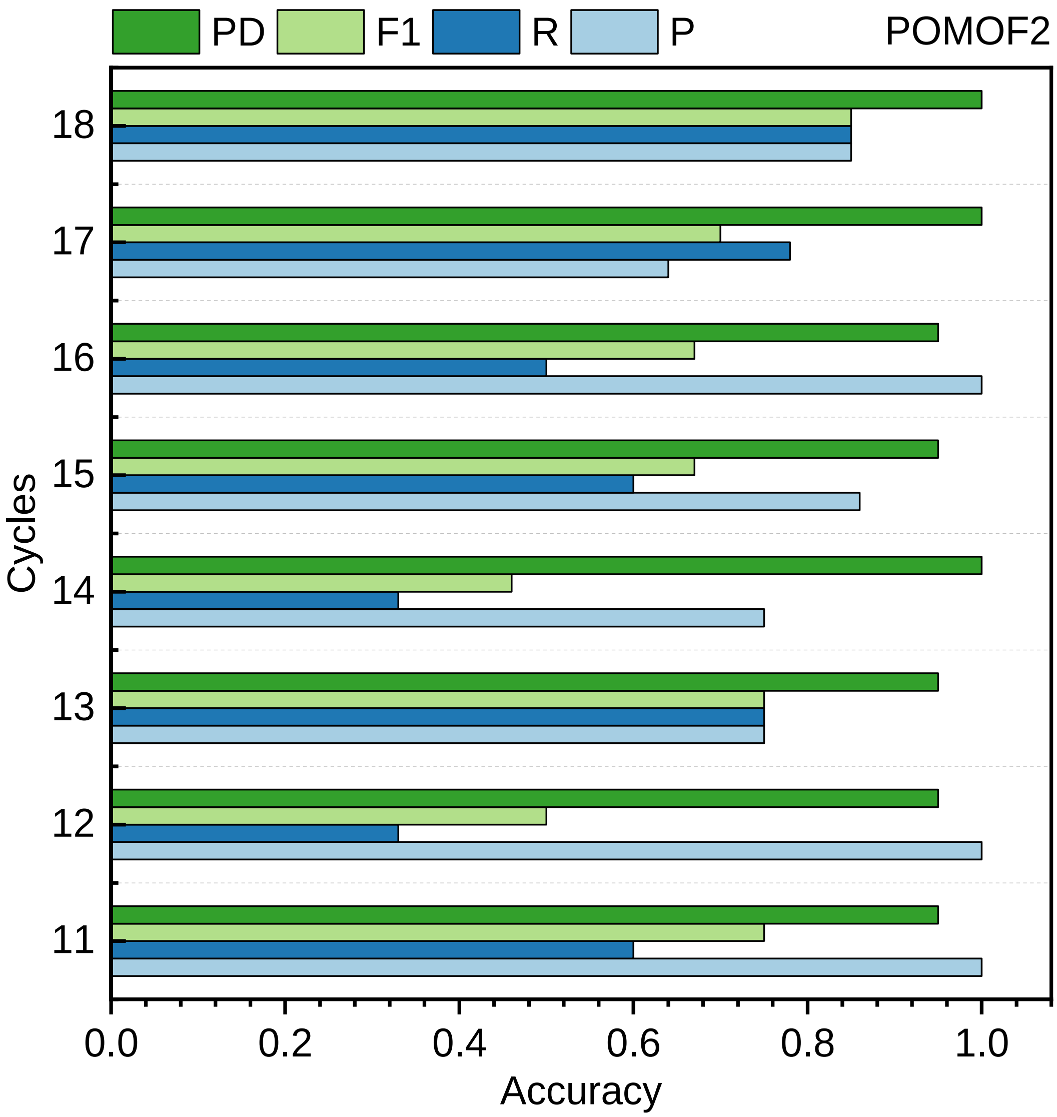

Figure S38: Accuracy of the model for POMOF2 from 11th the 18th optimization cycle after data reprocessed.

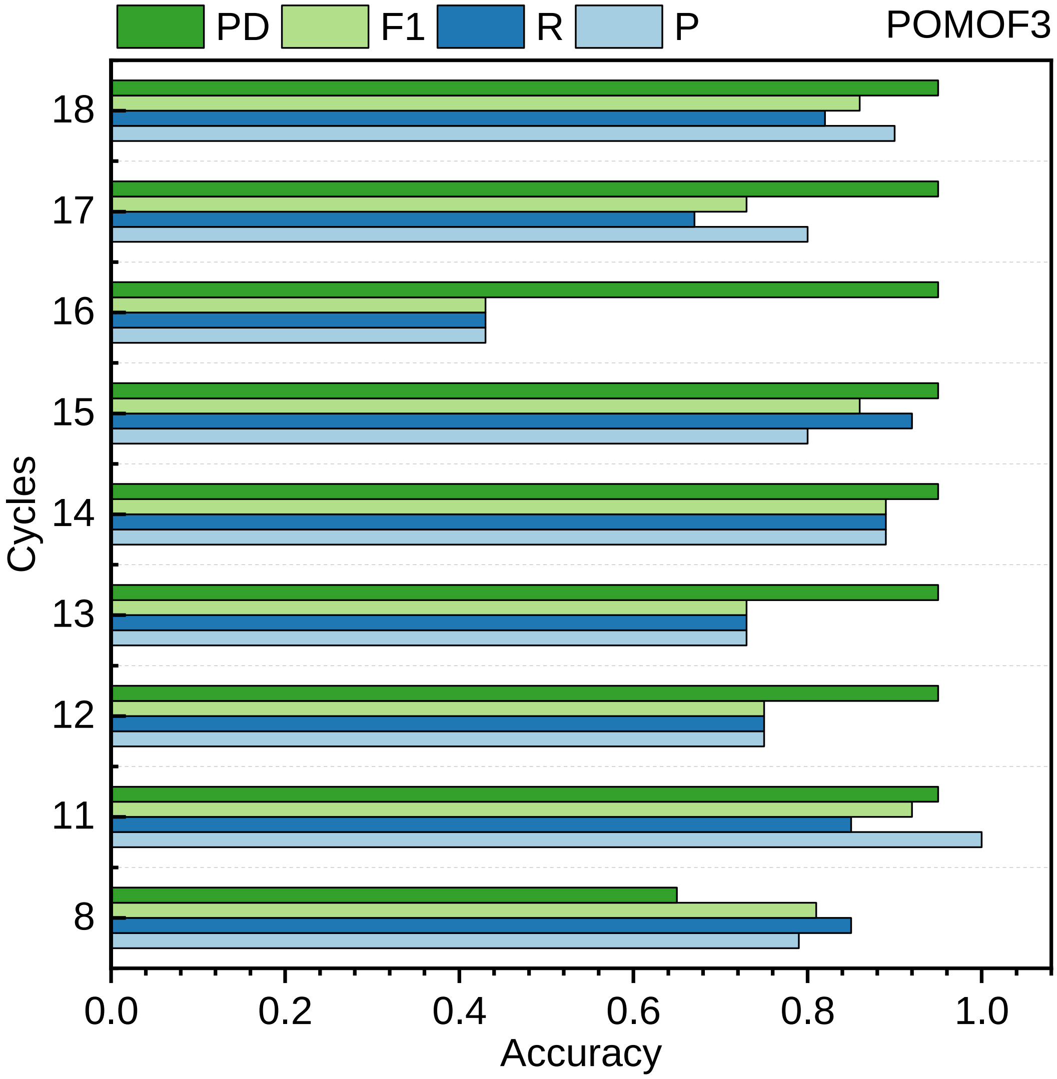

Figure S39: Accuracy of the model for POMOF3 based on the data collected by the optimization of POMOF2 model from 8th to the 18th optimization cycle.

4. Characterization

4.1 SC-XRD data

Table S57: SC-XRD data for POMOF1-1, POMOF1-2 and POMOF1+3.

Identification code	POMOF1-1	POMOF1-2	POMOF1+3
Empirical formula	$C_{82}H_{142}Mn_2Mo_{12}N_{22}O_{62}Zn_3$	$C_{100}H_{184}Mn_2Mo_{12}N_{28}O_{68}Zn_3$	$C_{48}H_{86}MnMo_6N_9O_{29}Zn$
Formula weight	3885.44	4324.01	1949.20
Temperature	100(2) K	150(2) K	150(2) K
Crystal system	Cubic	Monoclinic	Triclinic
Space group	*I*432	*C*2/c	*P*-1
Unit cell dimensions	a = 36.91700(10) Å<br>b = 36.91700(10) Å<br>c = 36.91700(10) Å	a = 44.0728(5) Å<br>b = 13.24040(10) Å<br>c = 28.5222(3) Å<br>Β = 101.7260(10)°,	a = 13.5088(4) Å<br>b = 13.5310(4) Å<br>c = 22.8105(5) Å<br>α = 73.231(2)°<br>β = 82.078(2)°<br>γ = 60.252(3)°
Volume	50312.9(4) $Å^3$	16296.5(3) $Å^3$	3466.00(19) $Å^3$
Z	12	4	2
Density (calculated)	1.539 Mg/m^3	1.762 Mg/m^3	1.868 Mg/m^3
Absorption coefficient	1.283 mm^{-1}	1.562 mm^{-1}	1.651 mm^{-1}
F(000)	23136	8672	1952
Crystal size	0.080 x 0.080 x 0.080 mm^3	0.120 x 0.050 x 0.020 mm^3	0.150 x 0.050 x 0.030 mm^3
Theta range	0.756 to 25.991°.	2.379 to 26.000°.	2.232 to 25.999°.
Index ranges	-46<=h<=46,<br>-46<=k<=46,<br>-46<=l<=46	-54<=h<=54,<br>-16<=k<=16,<br>-33<=l<=35	-16<=h<=16,<br>-16<=k<=16,<br>-28<=l<=28
Reflections collected	357834	252515	60286
Independent reflections	9081 [R(int) = 0.0489]	16012 [R(int) = 0.0729]	13593 [R(int) = 0.0775]
Completeness to theta	100.0% (theta = 24.415°)	99.9% (theta = 25.242°)	99.8% (theta = 25.242°)
Data / restraints / parameters	9081 / 35 / 326	16012 / 18 / 912	13593 / 3 / 735
Goodness-of-fit on F^2	1.077	1.025	1.021
Final R indices [I>2sigma(I)]	R1 = 0.0527,<br>wR2 = 0.1674	R1 = 0.0308,<br>wR2 = 0.0795	R1 = 0.0603,<br>wR2 = 0.1543
R indices (all data)	R1 = 0.0549,<br>wR2 = 0.1699	R1 = 0.0375,<br>wR2 = 0.0823	R1 = 0.0926,<br>wR2 = 0.1725
Absolute structure parameter	0.08(3)	-	-
Largest diff. peak and hole	1.459 and -0.593 e.$Å^{-3}$	0.918 and -0.767 e.$Å^{-3}$	1.993 and -1.045 e.$Å^{-3}$

Table S58: SC-XRD data for POMOF2-1, POMOF2-2 and POMOF2-3.

Identification code	POMOF2-1	POMOF2-2	POMOF2-3
Empirical formula	$C_{62}H_{121}MnMo_6N_{18}O_{39}Zn_2$	$C_{51}H_{93}MnMo_6N_{10}O_{29}Zn$	$C_{100}H_{166}Mn_2Mo_{12}N_{26}O_{66}Zn_3$
Formula weight	2504.08	2006.30	4245.85
Temperature	150(2) K	100(2) K	100(2) K
Crystal system	Monoclinic	Monoclinic	Triclinic
Space group	*I*2/*a*	*P*2_1/*c*	*P*-1
Unit cell dimensions	a = 28.1277(3) Å<br>b = 14.16340(10) Å<br>c = 24.6397(3) Å<br>β= 95.9660(10)°	a = 13.12570(10) Å<br>b = 24.70400(10) Å<br>c = 22.28820(10) Å<br>β= 95.8080(10)°	a = 13.40080(10) Å<br>b = 13.52120(10) Å<br>c = 23.1182(2) Å<br>α = 84.1320(10)°<br>β = 80.7930(10)°<br>γ = 64.5370(10)°
Volume	9762.89(17) $Å^3$	7190.01(7) $Å^3$	3730.65(6) $Å^3$
Z	4	4	1
Density (calculated)	1.704 Mg/m^3	1.853 Mg/m^3	1.890 Mg/m^3
Absorption coefficient	1.440 mm^{-1}	1.457 mm^{-1}	1.448 mm^{-1}
F(000)	5072	4032	2120
Crystal size	0.110 x 0.050 x 0.020 mm^3	0.067 x 0.018 x 0.015 mm^3	0.067 x 0.018 x 0.015 mm^3
Theta range	2.369 to 26.000°.	1.512 to 25.999°.	0.865 to 25.999°.
Index ranges	-34<=h<=34,<br>-17<=k<=17,<br>-30<=l<=30	-16<=h<=16,<br>-31<=k<=31,<br>-28<=l<=28	-17<=h<=17, -<br>17<=k<=17,<br>-29<=l<=29
Reflections collected	155651	99276	50648
Independent reflections	9587 [R(int) = 0.0530]	15531 [R(int) = 0.0555]	15864 [R(int) = 0.0389]
Completeness to theta	99.8% (theta = 25.242°)	99.9% (theta = 24.415°)	98.7% (theta = 24.415°)
Data / restraints / parameters	9587 / 35 / 457	15531 / 6 / 907	15864 / 82 / 968
Goodness-of-fit on F^2	1.065	1.055	1.054
Final R indices [I>2sigma(I)]	R1 = 0.0432,<br>wR2 = 0.1356	R1 = 0.0334,<br>wR2 = 0.0856	R1 = 0.0349,<br>wR2 = 0.0834
R indices (all data)	R1 = 0.0485,<br>wR2 = 0.1397	R1 = 0.0384,<br>wR2 = 0.0877	R1 = 0.0391,<br>wR2 = 0.0849
Largest diff. peak and hole	1.237 and -0.700 e.$Å^{-3}$	1.400 and -0.541 e.$Å^{-3}$	1.838 and -1.015 e.$Å^{-3}$

Table S59: SC-XRD data for POMOF3-1, POMOF3-2 and POMOF3-3.

Identification code	POMOF3-1	POMOF3-2	POMOF3-3
Empirical formula	$C_{44}H_{77}MnMo_6N_{12}O_{34}Zn_2$	$C_{100}H_{183.60}Mn_2Mo_{12}N_{28}O_{72}Zn_{3.20}$	$C_{57}H_{106.80}MnMo_6N_{12}O_{33}Zn_{1.10}$
Formula weight	2079.49	4400.68	2190.82
Temperature	100(2) K	150(2) K	150(2) K
Crystal system	Tetragonal	Monoclinic	Monoclinic
Space group	*I*4₁/*acd*	*C*2/*c*	*C*2/*c*
Unit cell dimensions	a = 26.68020(10) Å	a = 44.0703(3) Å	a = 23.1850(11) Å
	b = 26.68020(10) Å	b = 13.32030(10) Å	b = 13.6394(9) Å
	c = 39.4222(2) Å	c = 28.66850(10) Å	c = 24.501(2) Å
		β = 102.82°	β = 117.400(5)°
Volume	28062.0(3) $Å^3$	16409.54(18) $Å^3$	6878.7(9) $Å^3$
Z	16	4	4
Density (calculated)	1.969 Mg/m^3	1.781 Mg/m^3	2.116 Mg/m^3
Absorption coefficient	1.689 mm^{-1}	1.583 mm^{-1}	1.715 mm^{-1}
F(000)	16544	8822	4427
Crystal size	0.080 x 0.050 x 0.040 mm^3	0.390 x 0.270 x 0.160 mm^3	0.090 x 0.040 x 0.030 mm^3
Theta range	1.448 to 25.997°.	2.313 to 26.000°.	2.902 to 24.749°.
Index ranges	-33<=h<=33,	-54<=h<=54,	-27<=h<=27,
	-33<=k<=33,	-16<=k<=16,	-16<=k<=16,
	-50<=l<=50	-35<=l<=35	-28<=l<=28
Reflections collected	187902	257539	52047
Independent reflections	7575 [R(int) = 0.0804]	16123 [R(int) = 0.0447]	5864 [R(int) = 0.0880]
Completeness to theta	99.9% (theta = 24.415°)	99.8% (theta = 25.242°)	99.8% (theta = 24.749°)
Data / restraints / parameters	7575 / 26 / 382	16123 / 23 / 927	5864 / 19 / 320
Goodness-of-fit on F^2	1.114	1.072	1.045
Final R indices [I>2sigma(I)]	R1 = 0.0584, wR2 = 0.1907	R1 = 0.0306, wR2 = 0.0839	R1 = 0.0962, wR2 = 0.2300
R indices (all data)	R1 = 0.0683, wR2 = 0.1990	R1 = 0.0327, wR2 = 0.0851	R1 = 0.1157, wR2 = 0.2414
Largest diff. peak and hole	1.301 and -2.400 e.$Å^{-3}$	1.426 and -0.821 e.$Å^{-3}$	1.97 and -1.50 e.$Å^{-3}$

Table S60: SC-XRD data for POM-Zn and POM-Zn'.

Identification code	POM-Zn	POM-Zn'
Empirical formula	$C_{70}H_{158}Mn_2Mo_{12}N_{22}O_{66}$ Zn_3	$C_{76}H_{172}Mn_2Mo_{12}N_{24}O_{68}Zn_3$
Formula weight	3821.44	3967.64
Temperature	150(2) K	150(2) K
Crystal system	Triclinic	Monoclinic
Space group	*P*-1	*I*2/*a*
Unit cell dimensions	a = 13.7825(2) Å<br>b = 13.8222(2) Å<br>c = 18.5764(2) Å<br>α = 110.8870(10)°<br>β = 102.3300(10)°<br>γ = 90.3430(10)°	a = 20.0700(3) Å<br>b = 23.0444(3) Å<br>c = 26.8828(3) Å<br>β = 96.5070(10)°
Volume	3216.96(8) $Å^3$	12353.2(3) $Å^3$
Z	1	4
Density (calculated)	1.973 Mg/m^3	2.133 Mg/m^3
Absorption coefficient	1.904 mm^{-1}	2.029 mm^{-1}
F(000)	1904	7936
Crystal size	0.210 x 0.150 x 0.080 mm^3	0.080 x 0.070 x 0.020 mm^3
Theta range	2.291 to 25.999°.	2.407 to 25.999°.
Index ranges	-17<=h<=17,<br>-17<=k<=17,<br>-22<=l<=22	-24<=h<=24,<br>-28<=k<=28,<br>-33<=l<=33
Reflections collected	80905	42018
Independent reflections	12600 [R(int) = 0.0318]	12084 [R(int) = 0.0235]s
Completeness to theta	99.9% (theta = 25.242°)	99.5% (theta = 25.242°)
Data / restraints / parameters	12600 / 0 / 790	12084 / 20 / 734
Goodness-of-fit on F^2	1.034	1.132
Final R indices [I>2sigma(I)]	R1 = 0.0248,<br>wR2 = 0.0640	R1 = 0.0341,<br>wR2 = 0.0727
R indices (all data)	R1 = 0.0280,<br>wR2 = 0.0654	R1 = 0.0406,<br>wR2 = 0.0746
Largest diff. peak and hole	0.699 and -0.723 e.$Å^{-3}$	0.989 and -0.697s e.$Å^{-3}$

Table S61: Summary of structures from SC-XRD data.†

Identification code	Anderson: L1: L2: L3	Mn:Zn total	Mn:Zn on network	Notes for Mn:Zn difference other than 2:3	Mn nodes n-connected	Zn nodes n-connected	Network type
POMOF1-1	2:4:0:0	2:3	1:1	Remaining Zn in solvent area	4	4	3-D cubic
POMOF1-2	2:4:0:0	2:3	2:3		4	3 + 2	3-D
POMOF2-1	1:0:2:0	1:2	1:2	OH- anion as ligand to balance charge	4	2	2-D layer
POMOF2-2	1:0:2:0	1:1	1:1	additional TBA cation to balance charge	4 + 2	3	3-D
POMOF2-3	2:0:4:0	2:3	2:3		4	3 + 2	3-D
POMOF3-1	1:0:0:2	1:2	1:1	Remaining Zn bind to imine/deprotonated phenol	4	4	3-D
POMOF3-2	2:0:0:4	2:3	2:3		4	3 + 2	3-D
POMOF3-3	1:0:0:2	1:1	1:1	additional TBA cation to balance charge	4	4	3-D
POMOF1+3	1:1:0:1	1:1	1:1	additional TBA cation to balance charge	4	4	3-D

Notes: † Mn node represents the Mn-Anderson cluster connection.

4.2 ICP and elemental analysis data

Table S62: Chemical composition summary.

Identification code	Experiment found						Calculated based on SC-XRD data					
	C	H	Mn	Mo	N	Zn	C	H	Mn	Mo	N	Zn
POMOF1-1	22.94	3.36	2.69	27.47	7.74	4.37	25.35	3.68	2.83	29.63	7.93	5.05
POMOF1-2	-	-	-	-	-	-	27.78	4.29	2.54	26.63	9.07	4.54
POMOF2-1	27.51	4.60	1.82	18.37	10.72	3.97	29.74	4.87	2.19	22.99	10.07	5.22
POMOF2-2	-	-	-	-	-	-	30.53	4.67	2.74	28.69	6.98	3.26
POMOF2-3	25.53	3.34	3.07	30.14	7.37	5.03	28.29	3.94	2.59	27.12	8.58	4.62
POMOF3-1	23.27	3.14	2.64	26.87	6.84	5.56	25.41	3.73	2.64	27.68	8.08	6.29
POMOF3-2	27.31	4.11	2.73	28.21	7.14	5.42	27.29	4.21	2.50	26.16	8.91	4.75
POMOF3-3	-	-	-	-	-	-	31.25	4.91	2.51	26.28	7.67	3.28
POMOF1+3	27.26	4.03	2.94	30.01	6.92	3.33	29.58	4.45	2.82	29.53	6.47	3.35
POM-Zn	18.03	3.30	2.63	27.22	6.34	4.72	22.00	4.17	2.88	30.13	8.06	5.13
POM-$(NH_2)_2$	34.46	6.52	3.12	31.85	4.81	-	35.74	6.64	2.92	30.58	3.72	-

The ICP and elemental analysis data are not available for POMOF1-2, POMOF2-2 and POMOF3-3 because the current synthesis of these POMOFs cannot obtain pure and enough products for the characterization.

The difference between the experimental data and calculated data from SC-XRD structure, can be attributed to the solvent loss after dried and the ultra-fast water absorption in the air for some samples.

4.3 PXRD

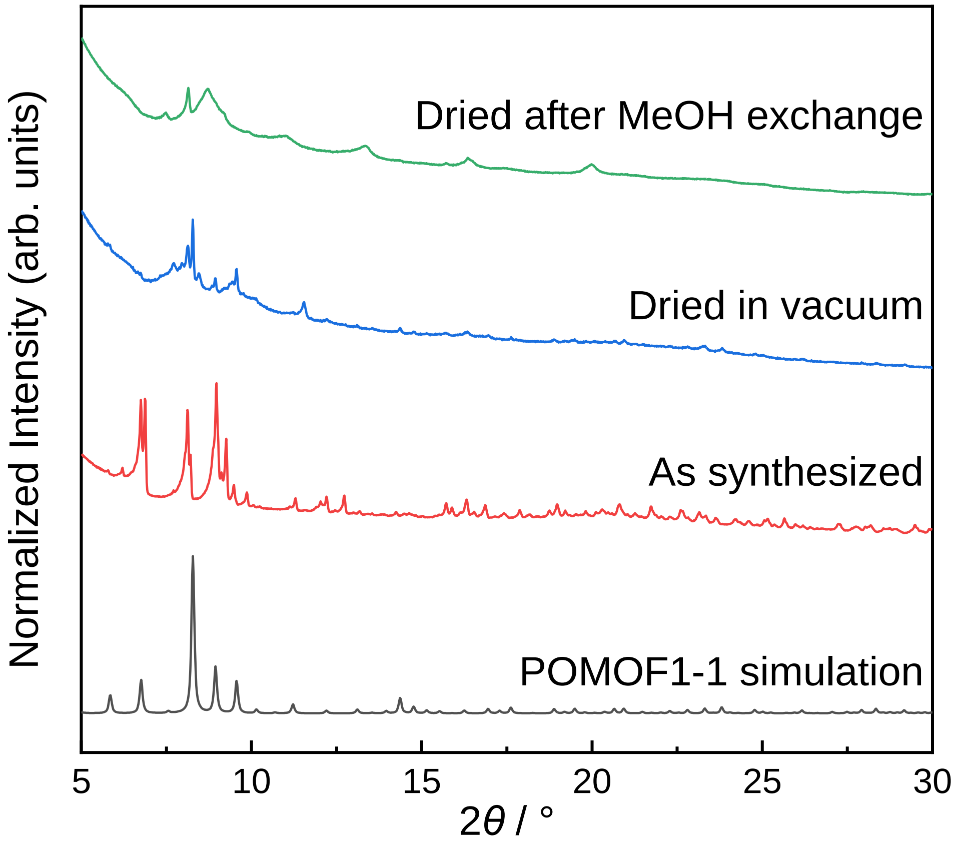

Figure S40: PXRD patterns for POMOF1-1 from simulation, as synthesized, after dried in vacuum at RT for 12 h, and after desolvated at 120 °C by solvent exchange.

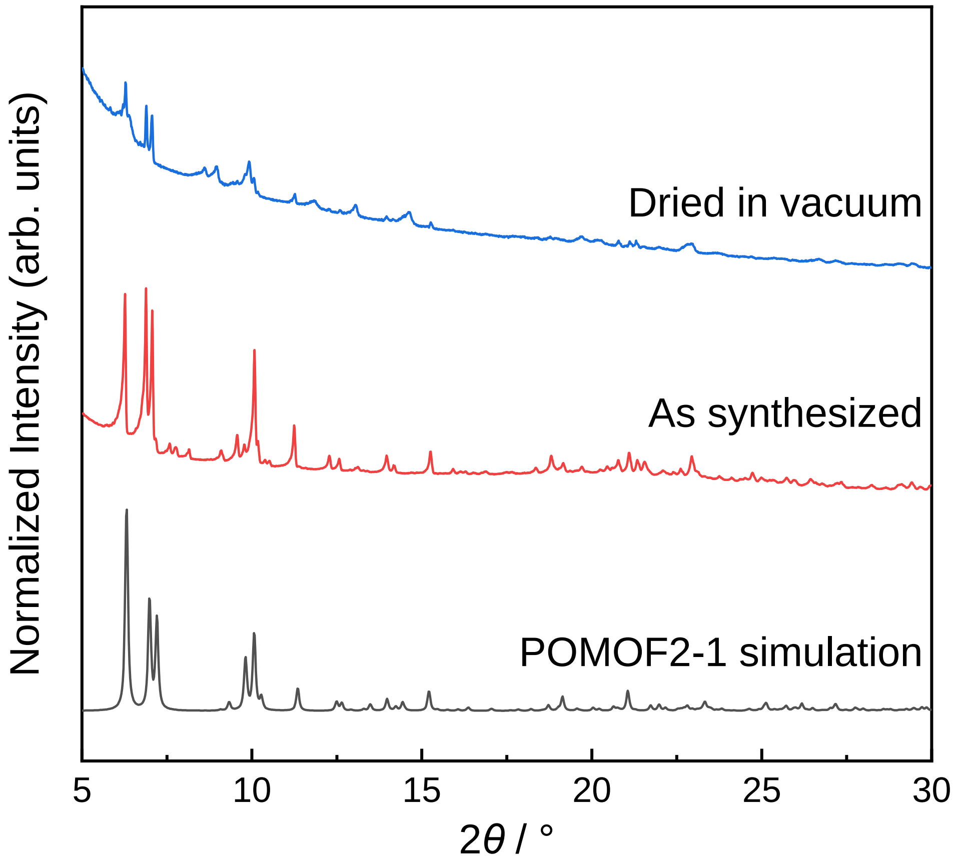

Figure S41: PXRD patterns for POMOF2-1 from simulation, as synthesized, and after dried in vacuum at RT for 12 h.

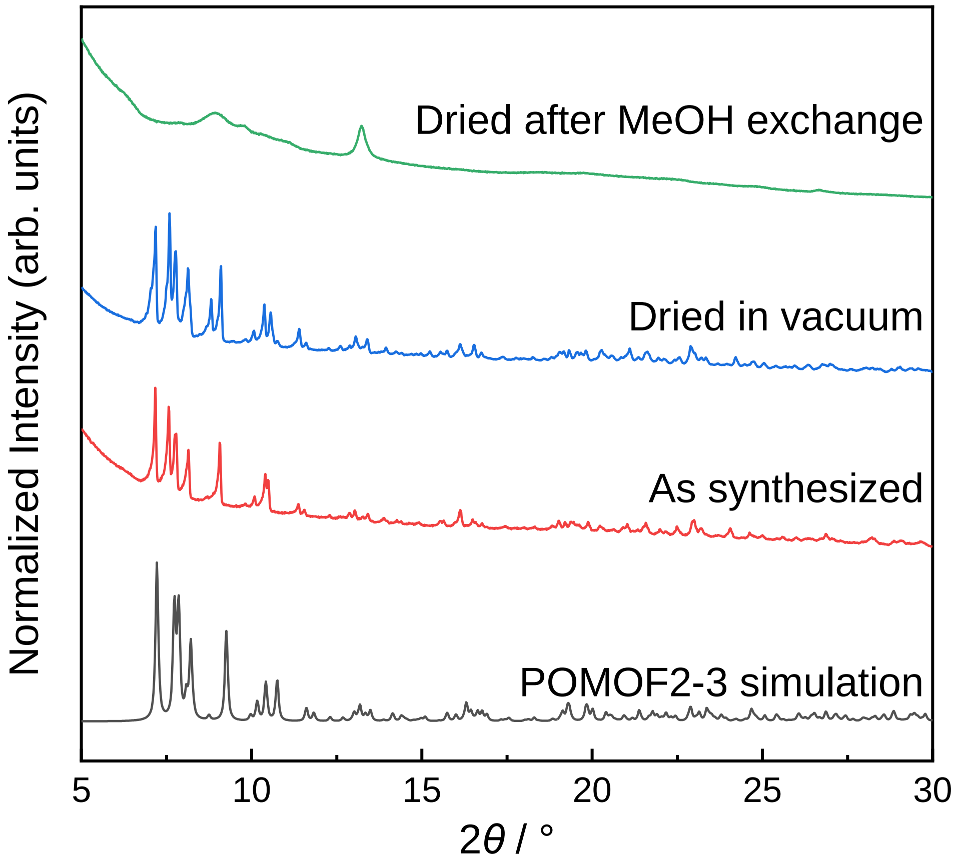

Figure S42: PXRD patterns for POMOF2-3 from simulation, as synthesized, after dried in vacuum at RT for 12 h, and desolvated at 120 °C for 12 h after solvent exchange.

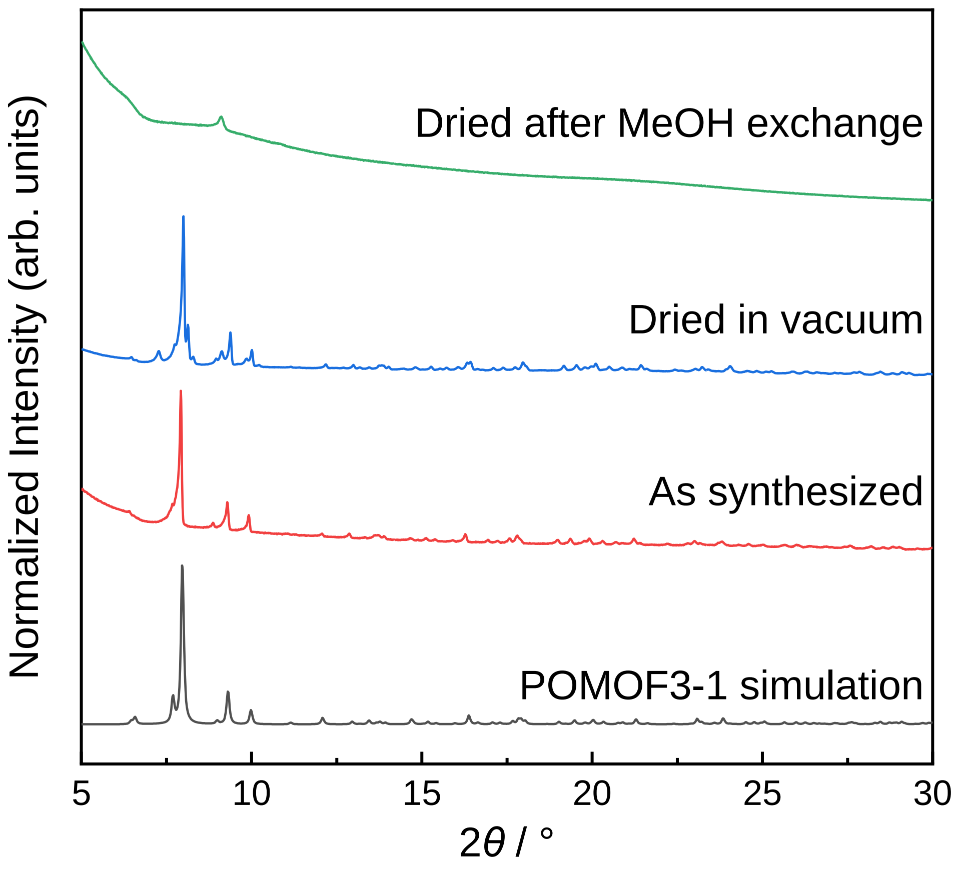

Figure S43: PXRD patterns for POMOF3-1 from simulation, as synthesized, after dried in vacuum at RT for 12 h, and desolvated at 120 °C for 12 h after solvent exchange.

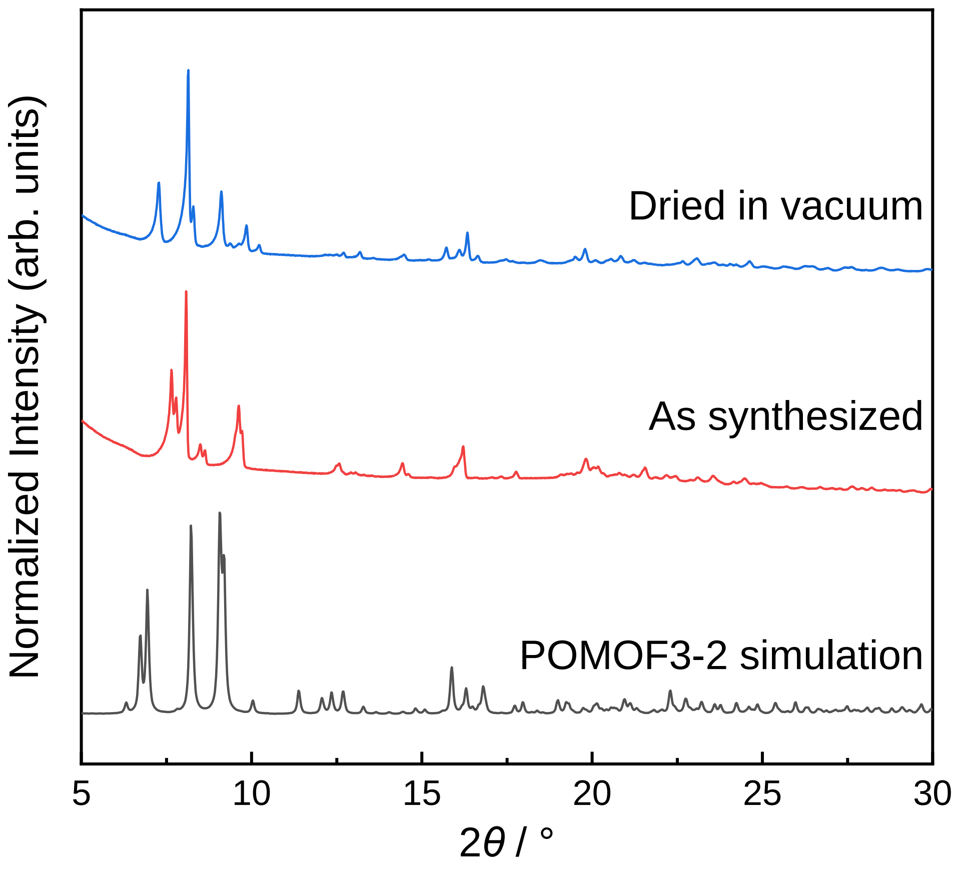

Figure S44: PXRD patterns for POMOF3-2 from simulation, as synthesized, and after dried in vacuum at RT for 12 h.

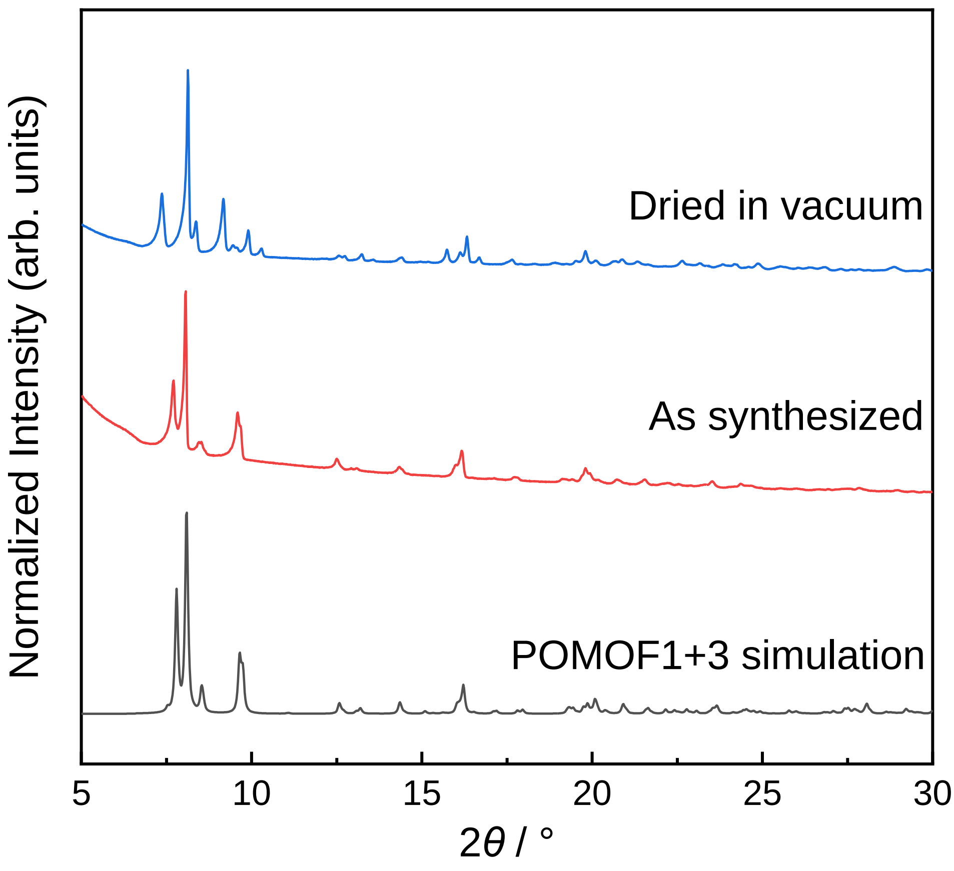

Figure S45: PXRD patterns for POMOF1+3 from simulation, as synthesized, and after dried in vacuum at RT for 12 h.

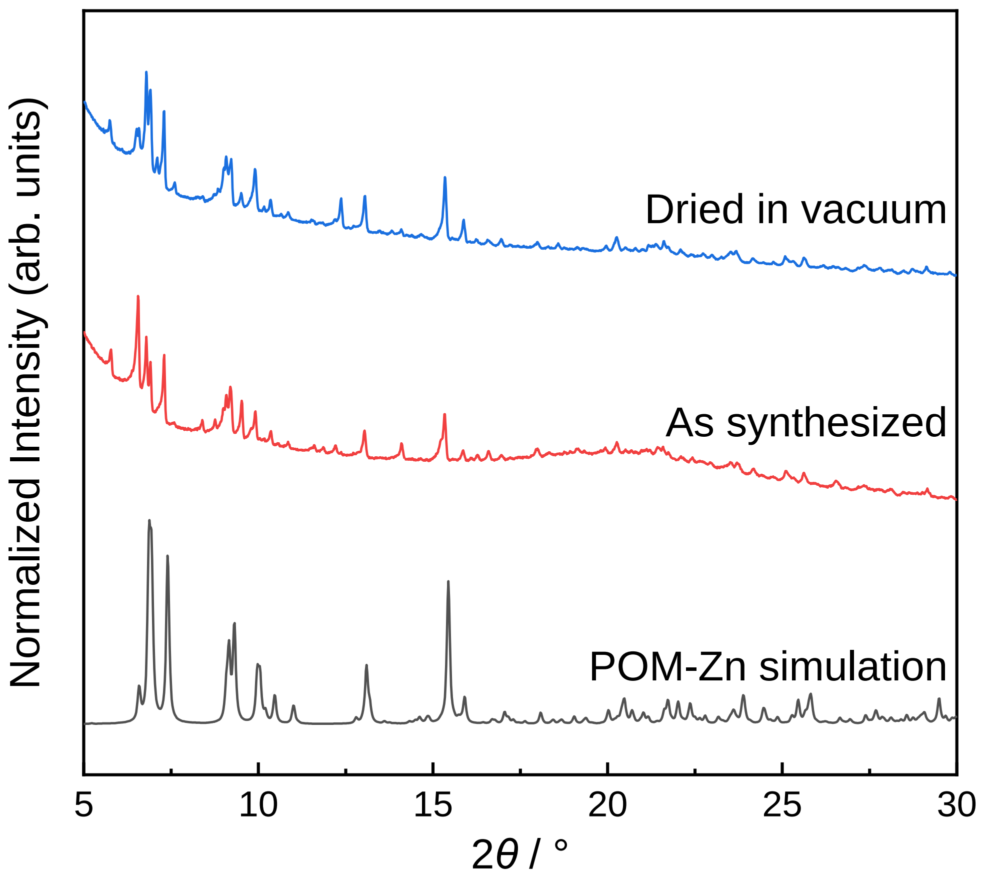

Figure S46: PXRD patterns for POMO-Zn from simulation, as synthesized, and after dried in vacuum at RT for 12 h.

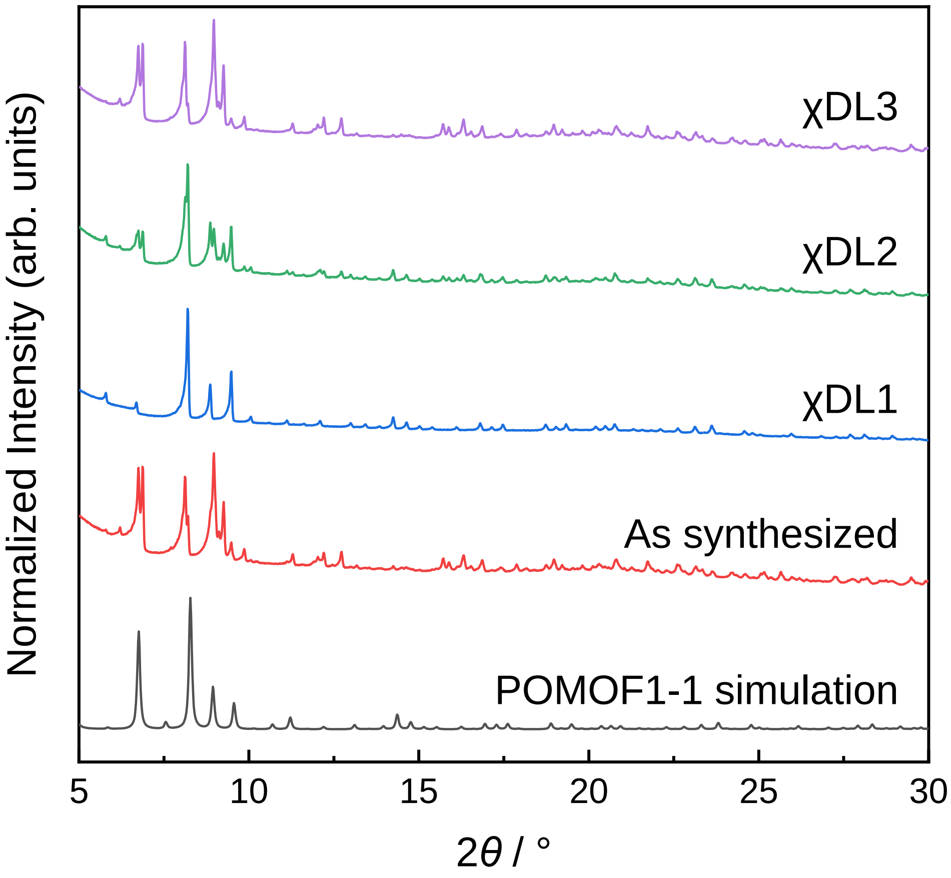

Figure S47: PXRD patterns for POMOF1-1 from simulation, as synthesized, and synthesized driven by χDL in three times.

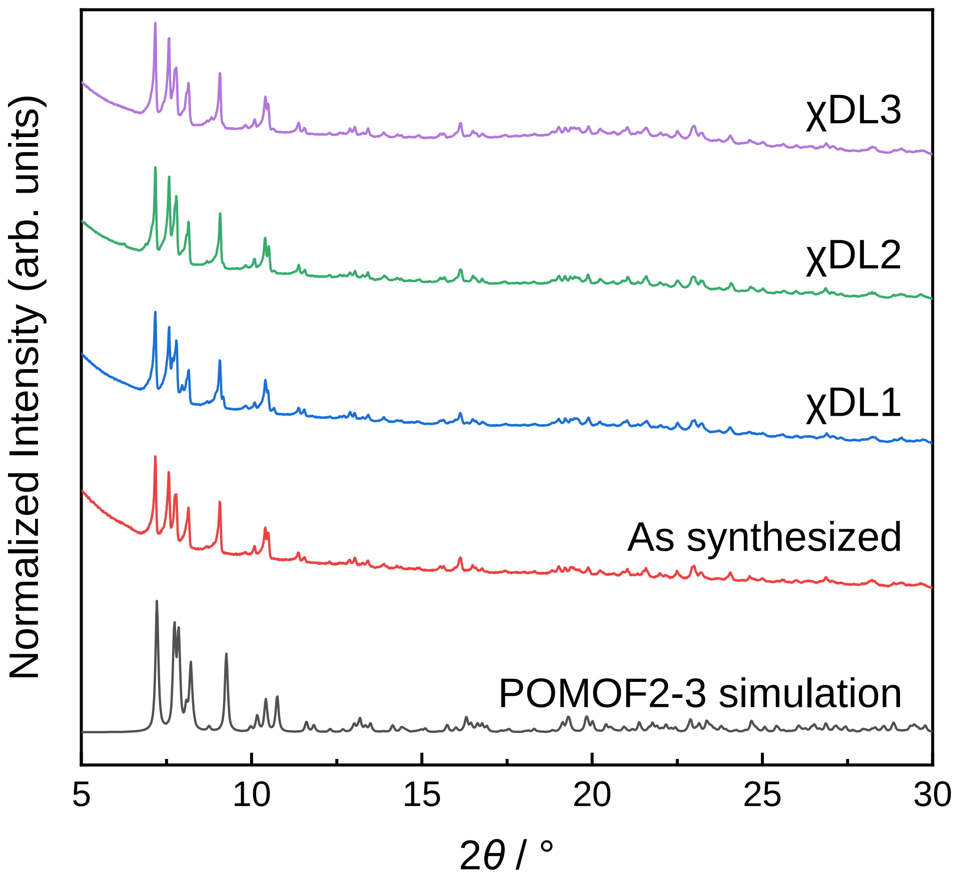

Figure S48: PXRD patterns for POMOF2-3 from simulation, as synthesized, and synthesized driven by χDL in three times.

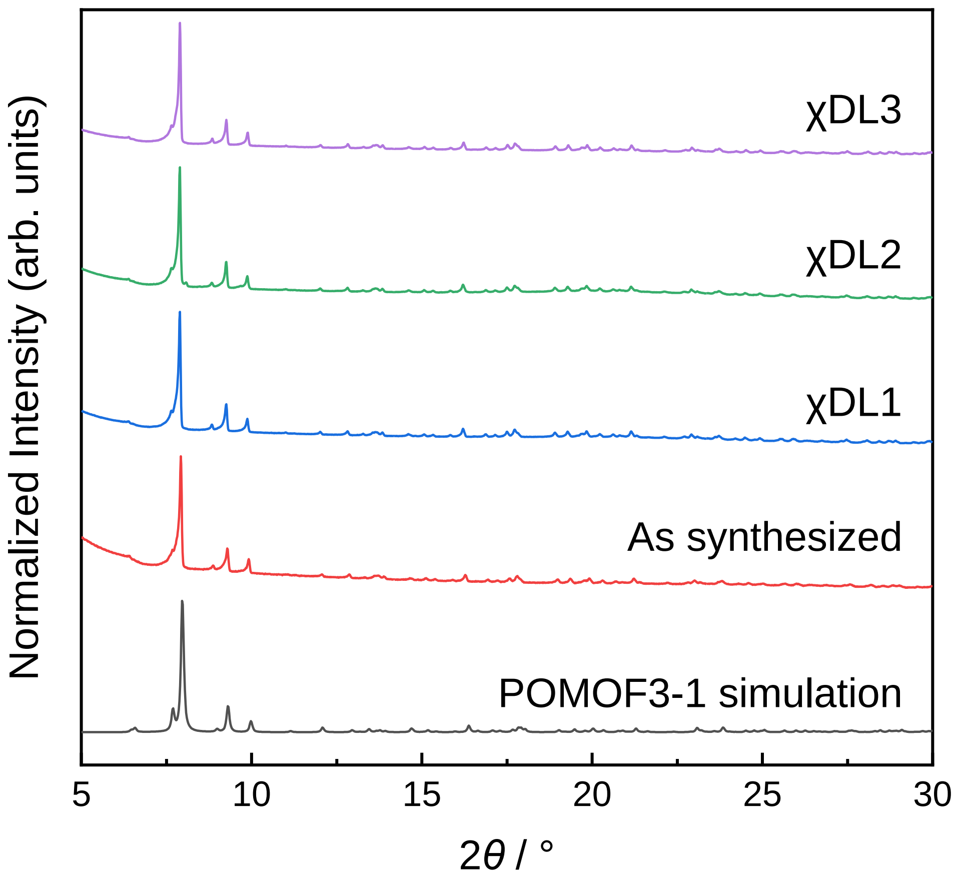

Figure S49: PXRD patterns for POMOF3-1 from simulation, as synthesized, and synthesized driven by χDL in three times.

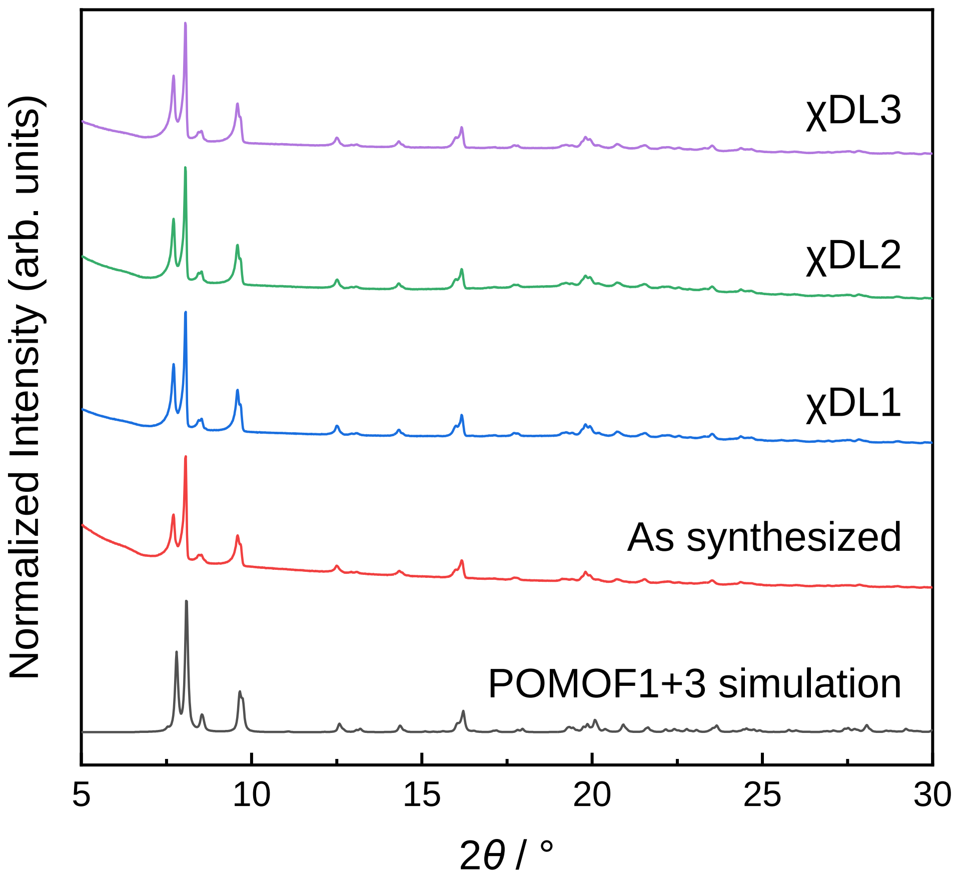

Figure S50: PXRD patterns for POMOF1+3 from simulation, as synthesized, and synthesized driven by χDL in three times.

4.4 IR spectra

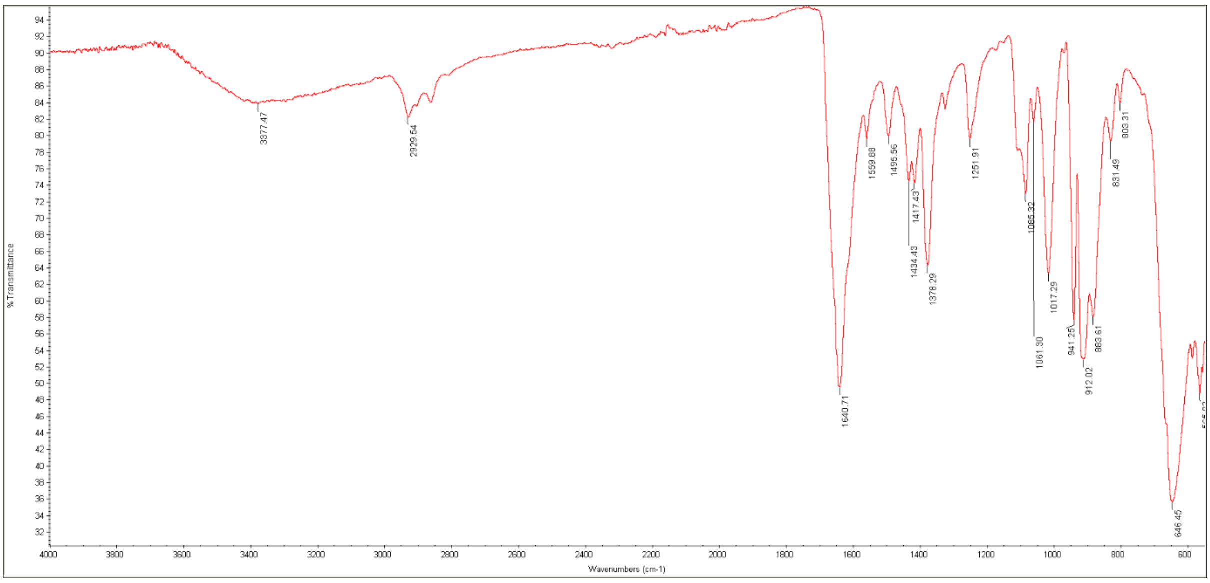

Figure S51: FT-IR spectra of POMOF1-1@DMF.

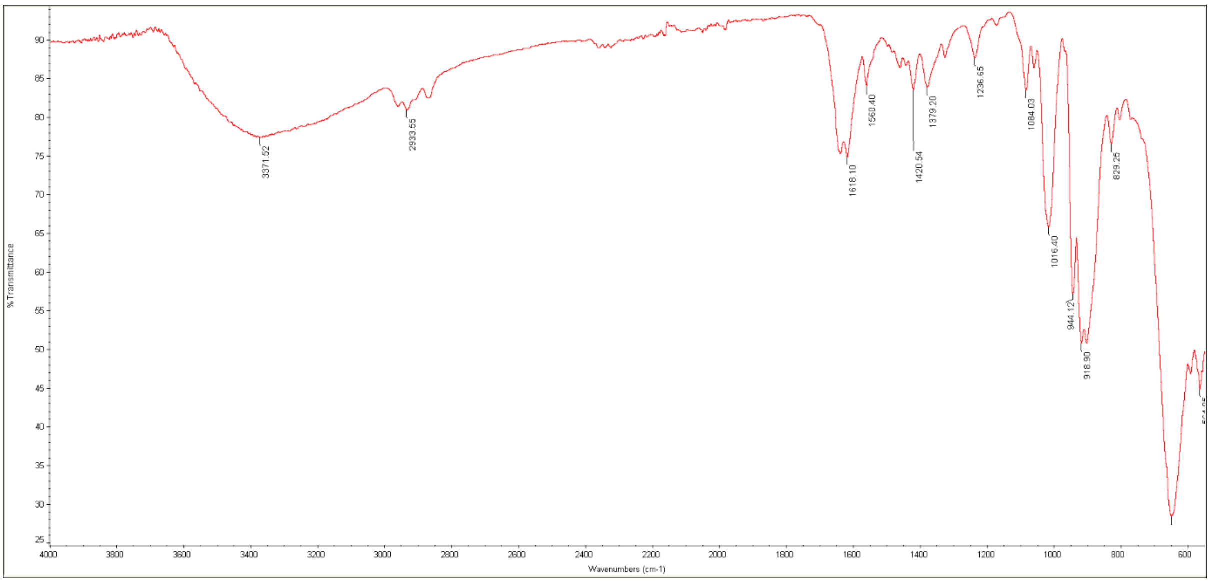

Figure S52: FT-IR spectra of POMOF1-1- desolvated.

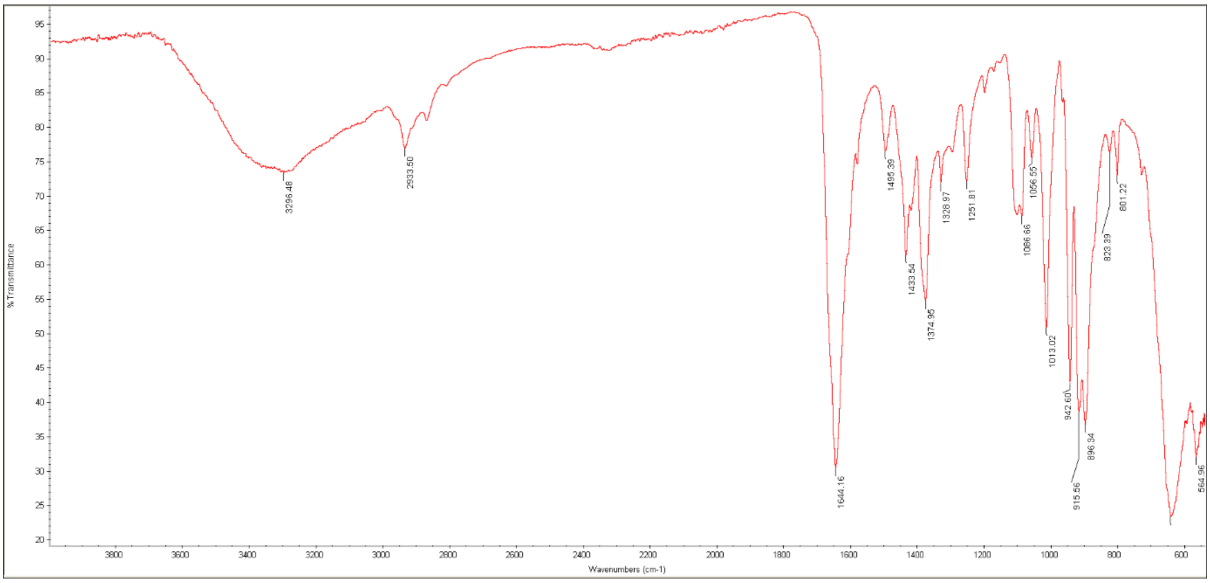

Figure S53: FT-IR spectra of POMOF2-1@DMF.

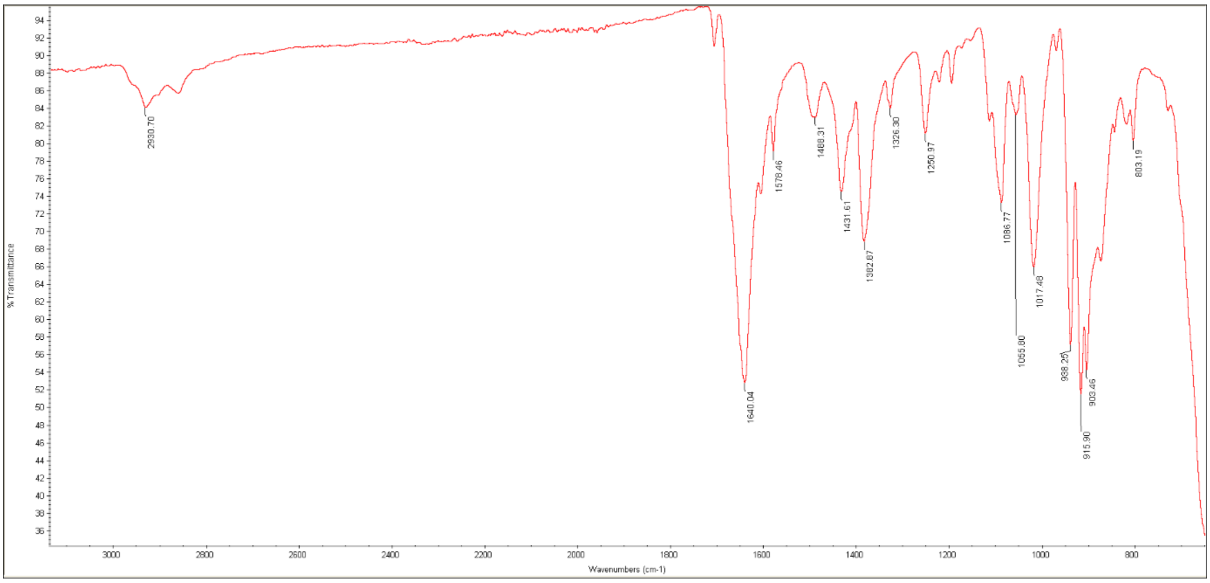

Figure S54: FT-IR spectra of POMOF2-2@DMF.

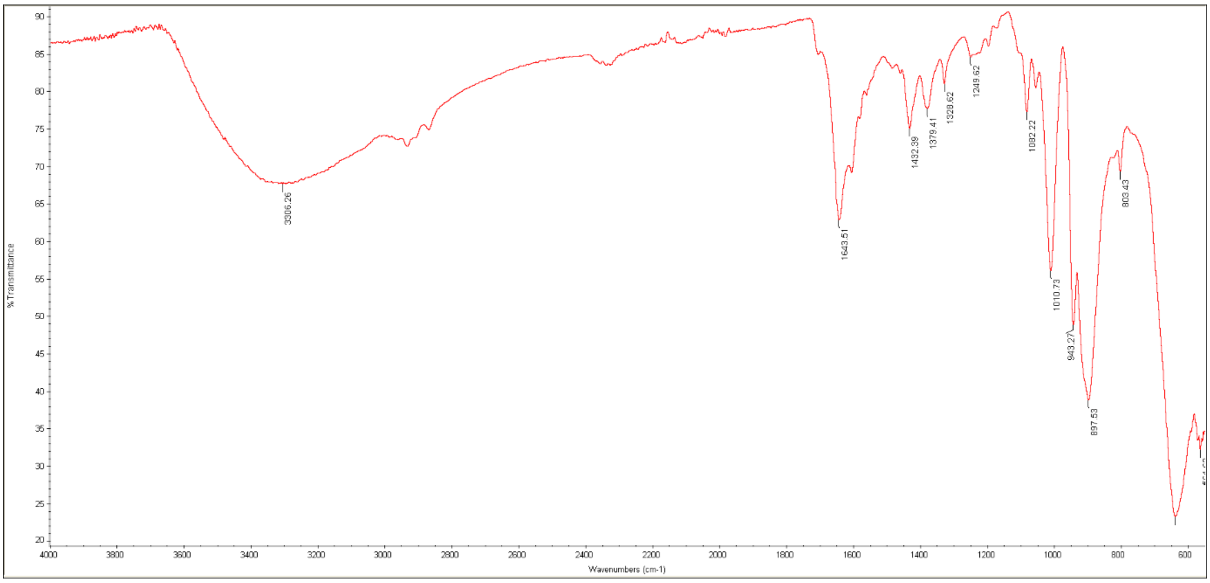

Figure S55: FT-IR spectra of POMOF2-2- desolvated.

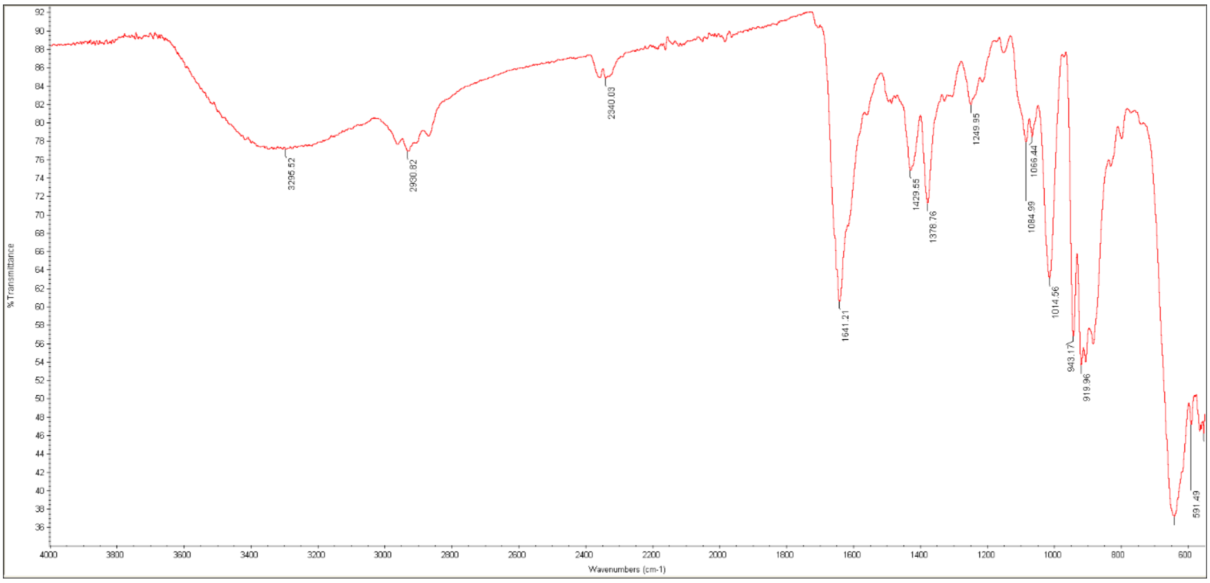

Figure S56: FT-IR spectra of POMOF3-1@DMF.

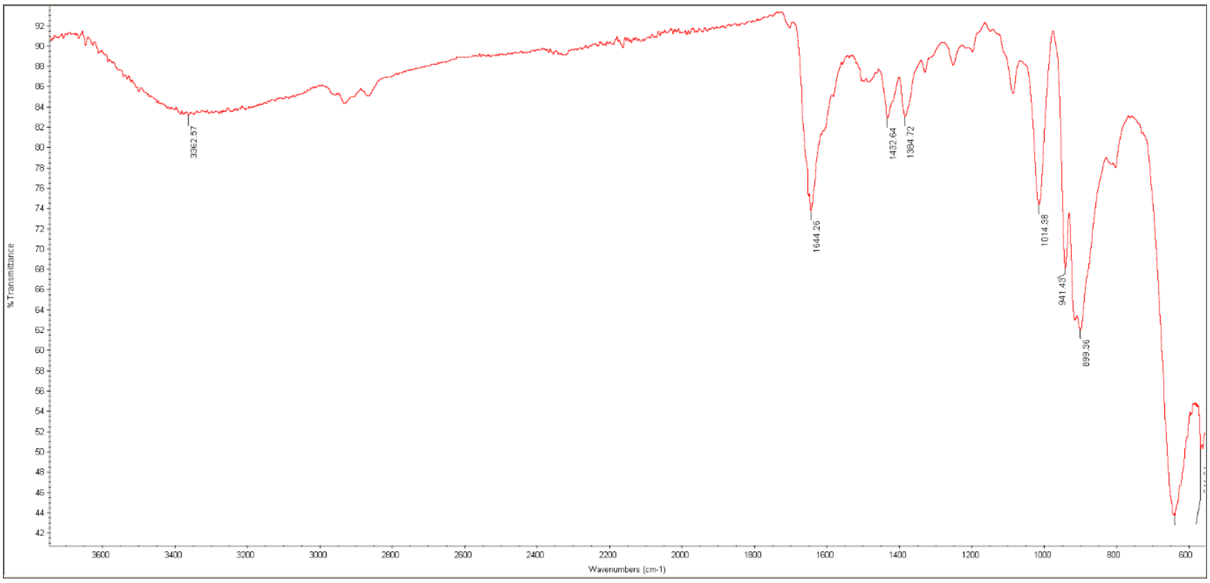

Figure S57: FT-IR spectra of POMOF3-1- desolvated.

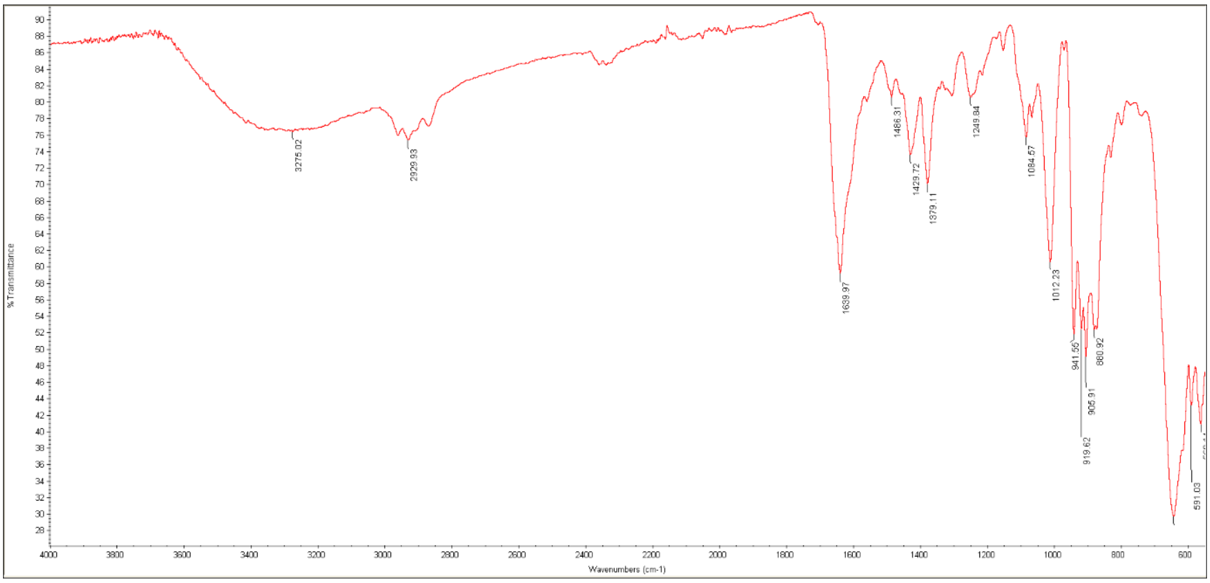

Figure S58: FT-IR spectra of POMOF3-2@DMF.

4.5 TGA data

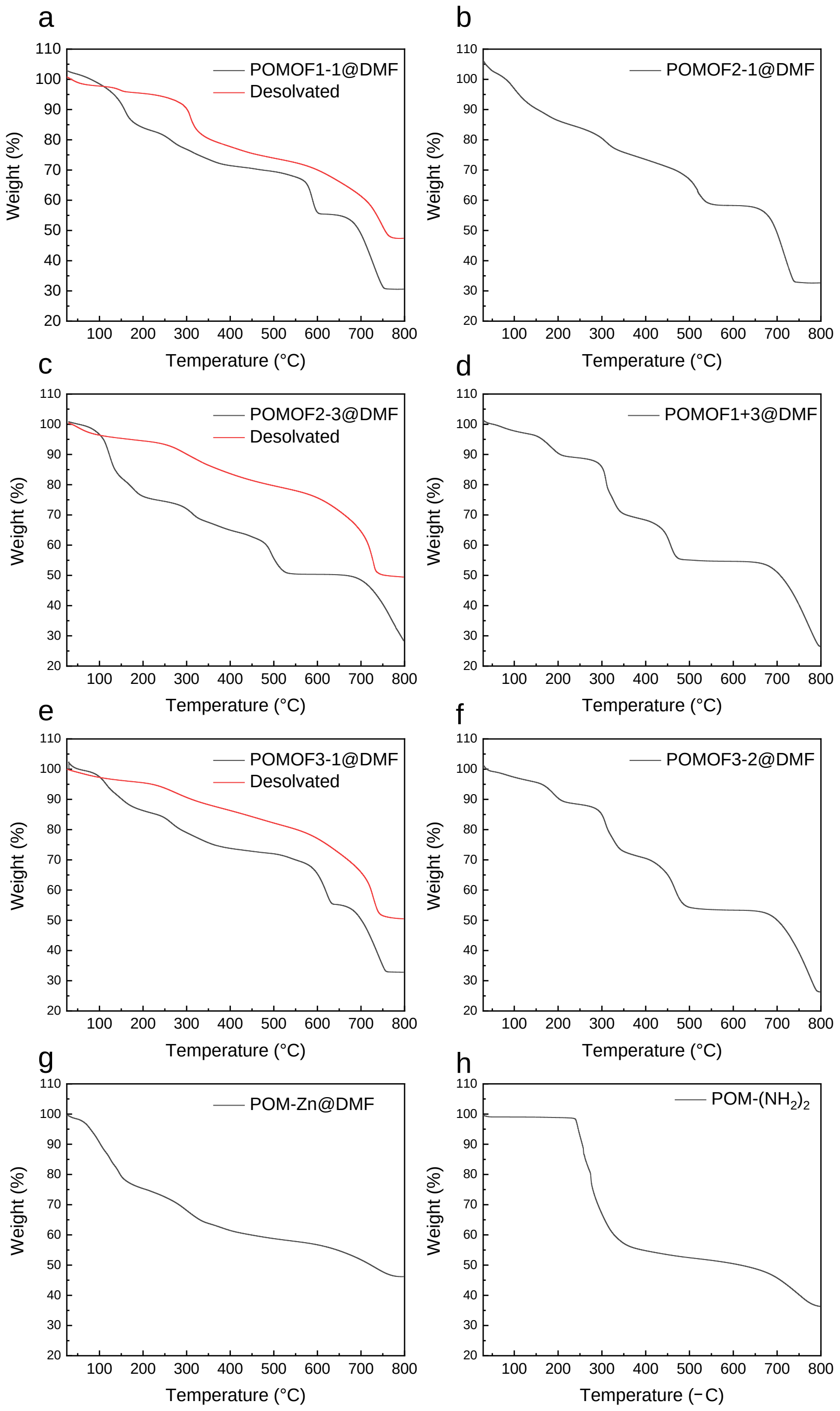

Figure S59: Thermogravimetric analysis of (a) POMOF1-1, (b) POMOF2-1, (c) POMOF2-3, (d) POMOF1+3, (e) POMOF3-1, (f) POMOF3-2, (g) POM-Zn, and (h) POM-$(NH_2)_2$.

4.6 Gas sorption

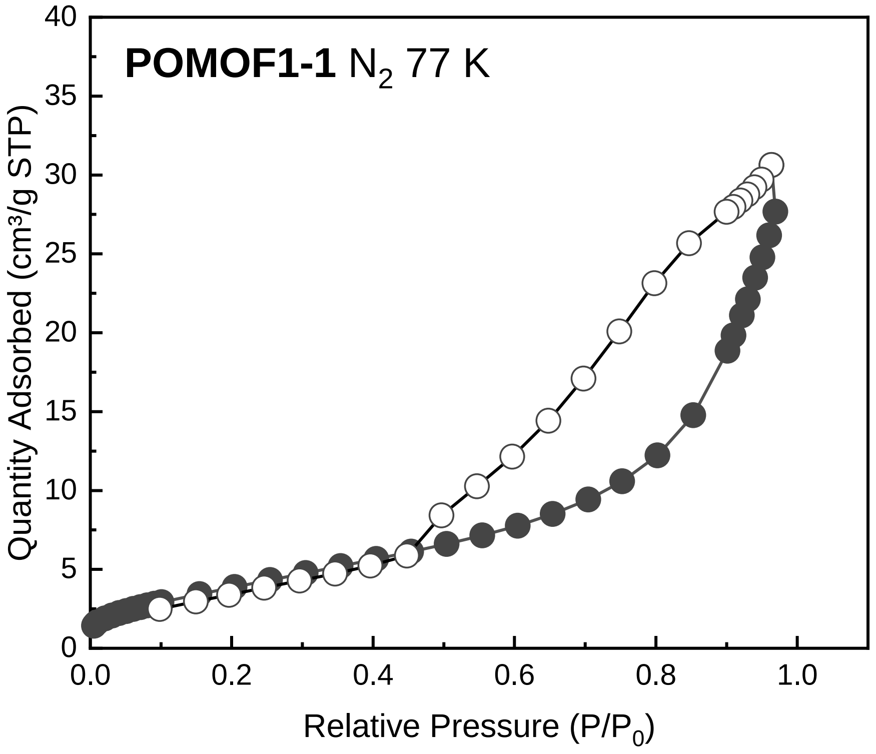

Figure S60: N_2 sorption isotherms (77 K) of POMOF1-1 exchanged with MeOH, dried at 120 °C in vacuum for 12 h.

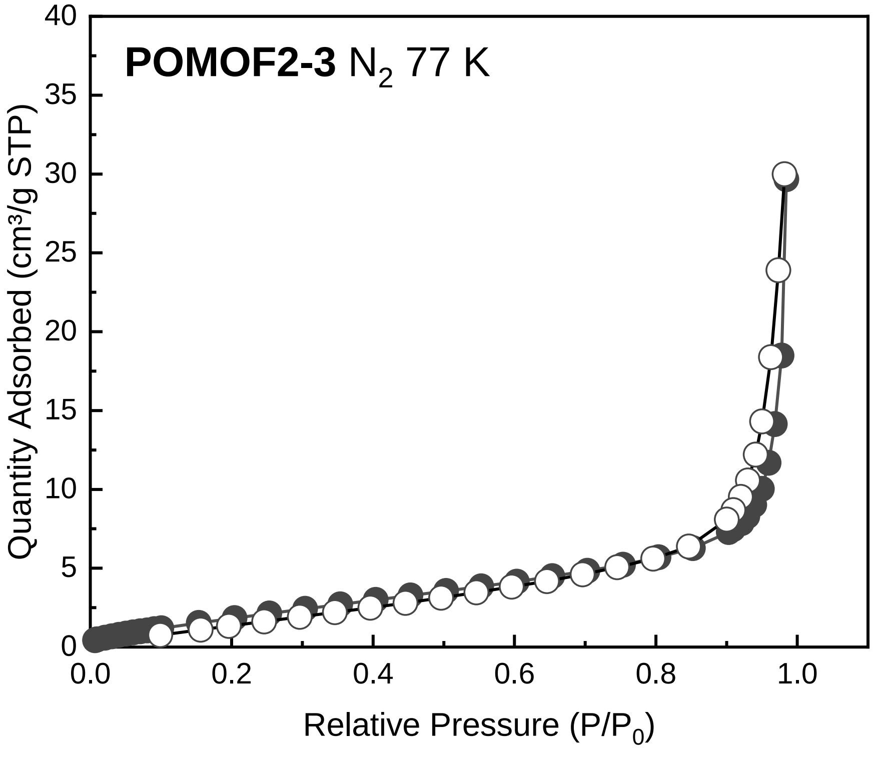

Figure S61: N_2 sorption isotherms (77 K) of POMOF2-3 exchanged with MeOH, dried at 120 °C in vacuum for 12 h.

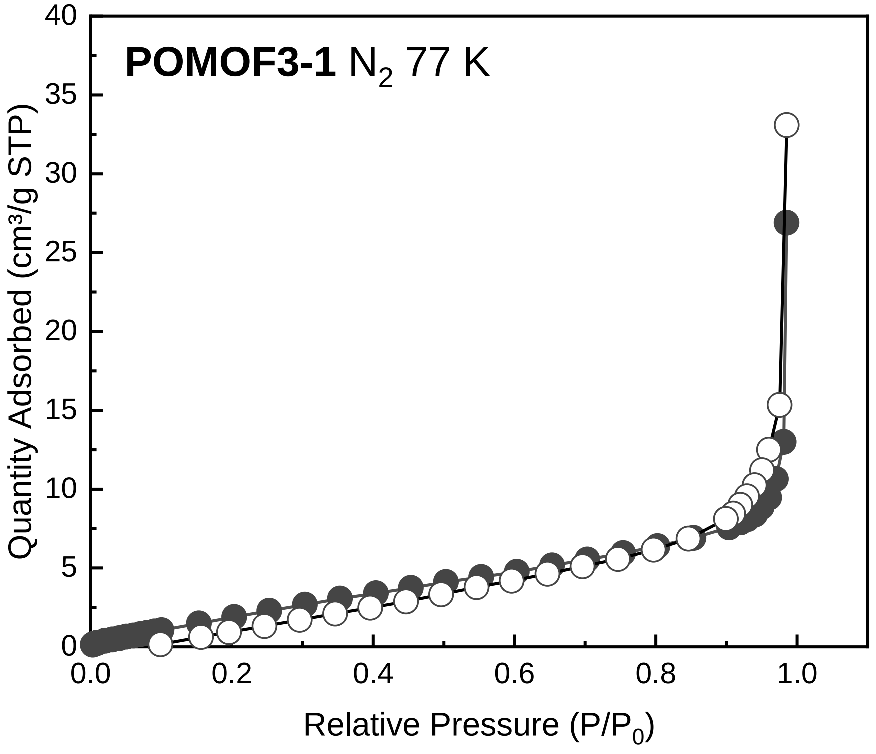

Figure S62: N_2 sorption isotherms (77 K) of POMOF3-1 exchanged with MeOH, dried at 120 °C in vacuum for 12 h.

4.7 Electrochemical properties

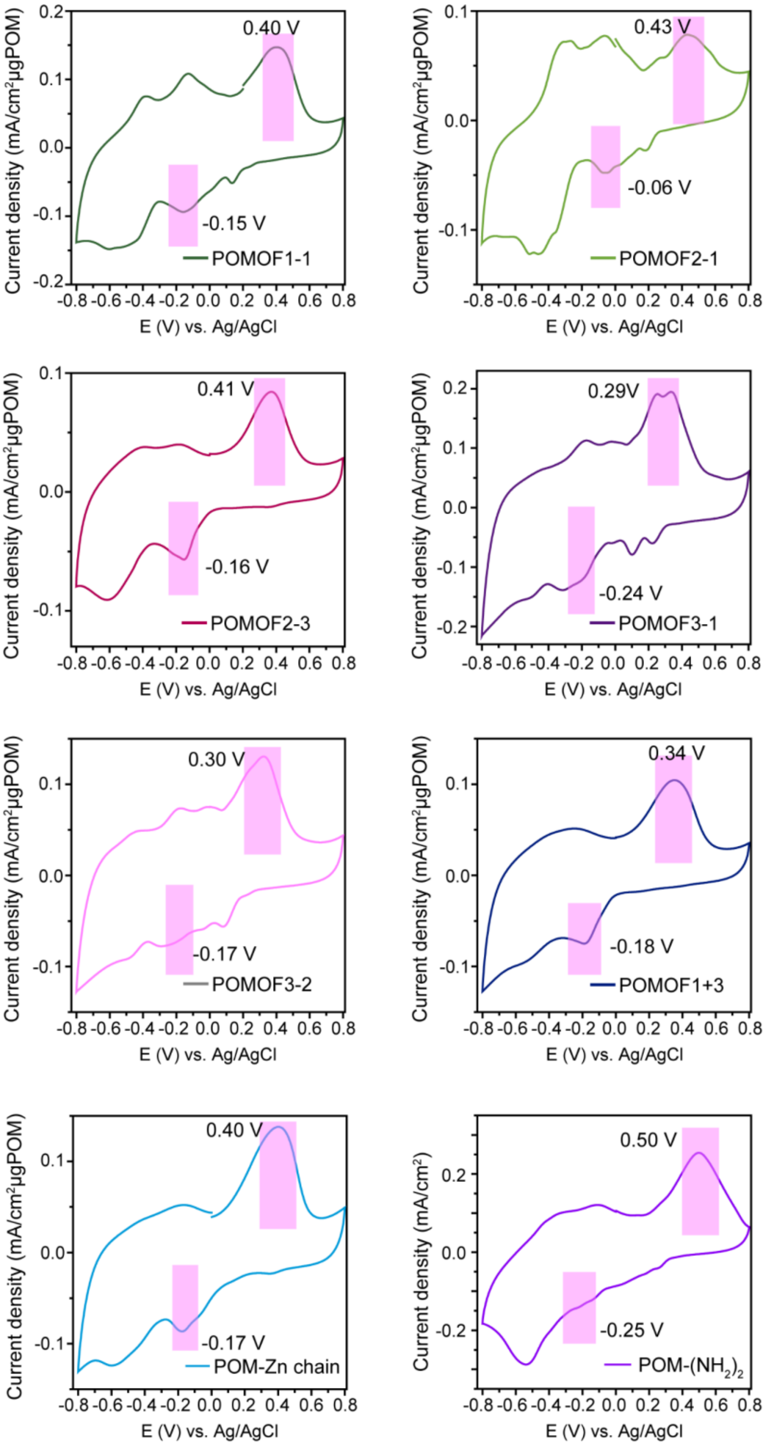

Figure S63: Cyclic voltammetry measurements for each of the POMOFs in N_2-saturated 0.5 mM H_2SO_4 + 2.5 mM Na_2SO_4 aqueous solution with the scan rate of 50 mV s^{-1}. POM-$(NH_2)_2$ and POM-Zn were also measured under the same conditions as controls. The pink bars highlight the positions of the anodic and cathodic peaks that were used to calculate the cathodic-anodic peak separation (ΔEp).

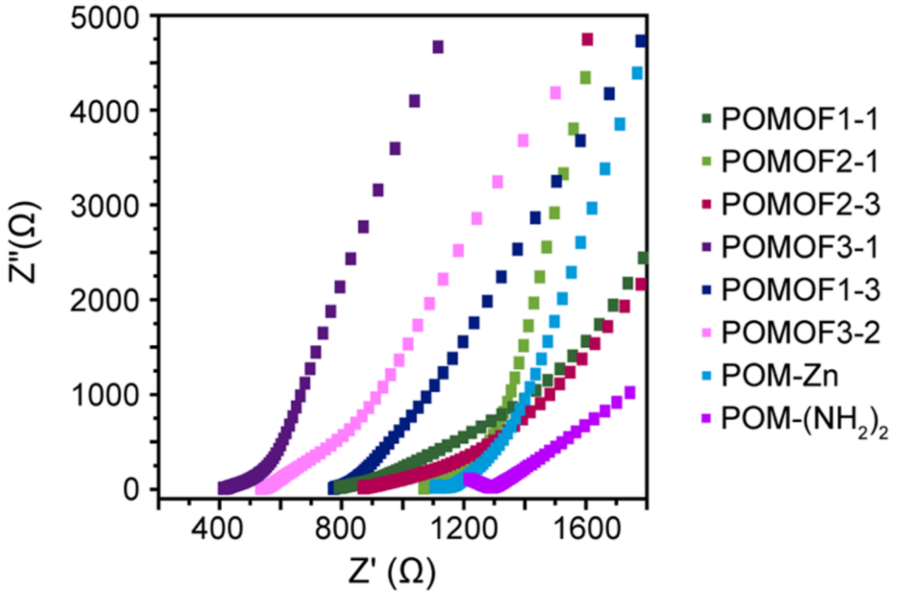

Figure S64: Electrochemical impedance spectroscopy (ESI) Nyquist plots for all the POMOFs taking POM-$(NH_2)_2$ and POM-Zn as control.

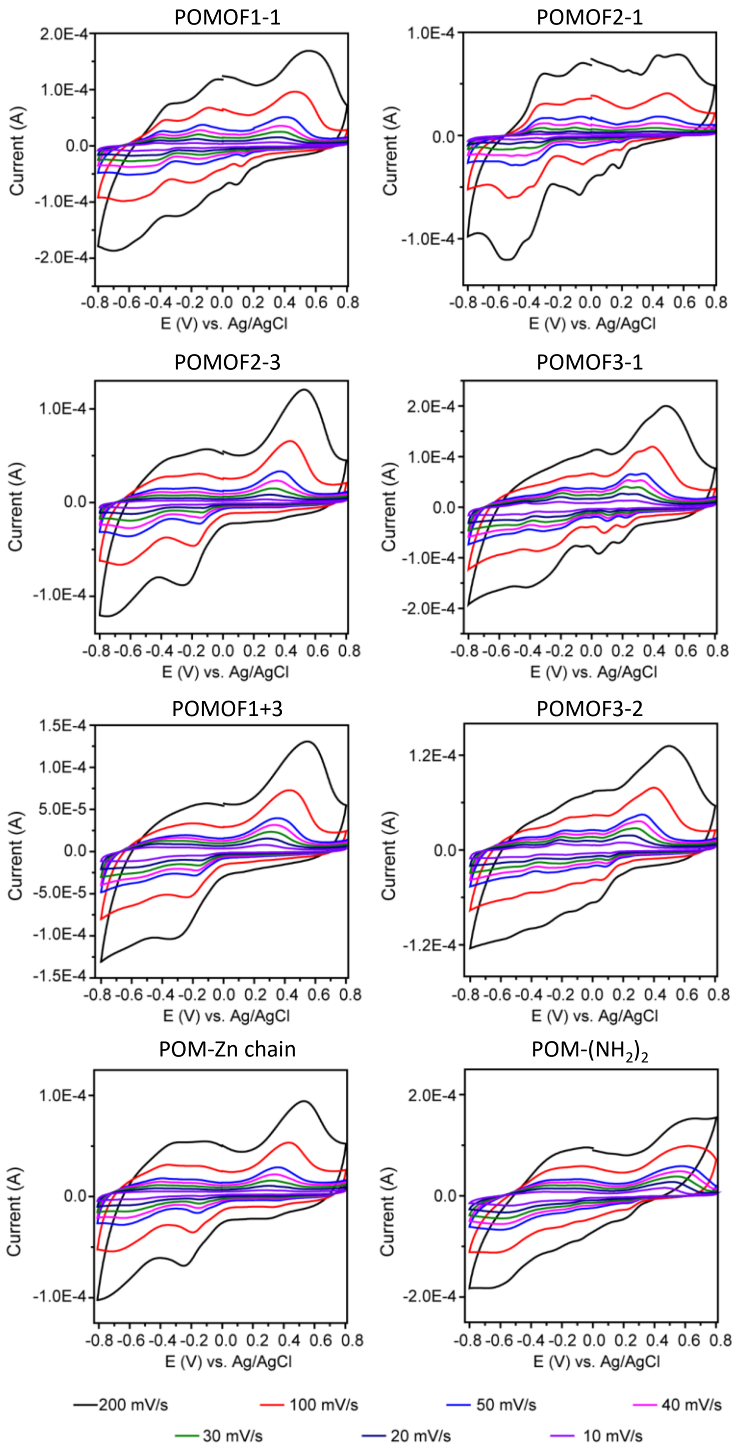

Figure S65: CV curves at various scan rates for all the POMOFs taking POM-$(NH_2)_2$ and POM-Zn as control.

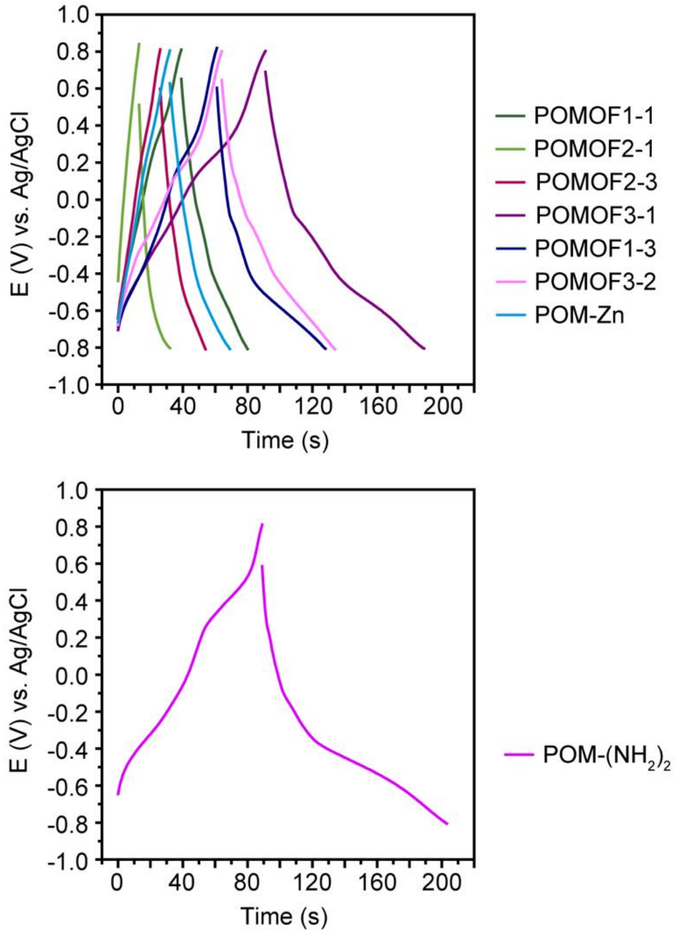

Figure S66: Charge-discharge (CD) curves for a) the POMOFs and b) POM-$(NH_2)_2$.

References

(1) Hasenknopf, B.; Delmont, R.; Herson, P.; Gouzerh, P., Anderson-Type Heteropolymolybdates Containing Tris(alkoxo) Ligands: Synthesis and Structural Characterization. *Eur. J. Inorg. Chem.* **2002,** *2002* (5), 1081-1087.

(2) Salley, D. S.; Keenan, G. A.; Long, D.-L.; Bell, N. L.; Cronin, L., A Modular Programmable Inorganic Cluster Discovery Robot for the Discovery and Synthesis of Polyoxometalates. *ACS Cent. Sci.* **2020,** *6* (9), 1587-1593.

(3) Mehr, S. H. M.; Craven, M.; Leonov, A. I.; Keenan, G.; Cronin, L., A Universal System for Digitization and Automatic Execution of the Chemical Synthesis Literature. *Science* **2020,** *370* (6512), 101-108.

(4) Jiang, Y.; Salley, D.; Sharma, A.; Keenan, G.; Mullin, M.; Cronin, L., An Artificial Intelligence Enabled Chemical Synthesis Robot for Exploration and Optimization of Nanomaterials. *Sci. Adv.* **2022,** *8* (40), eabo2626.
